# Prevalence of preserved ratio impaired spirometry and restrictive spirometry pattern in the general population: a systematic review and multi-level meta-analysis of studies from multiple countries

**DOI:** 10.7189/jogh.16.04072

**Published:** 2026-03-27

**Authors:** Wujian Xu, Sohail Ferdous, Bo Peng, Ting Shi

**Affiliations:** 1 Department of Pulmonary and Critical Care Medicine, Shanghai East Hospital, School of Medicine, Tongji University, Shanghai, China; 2 Usher Institute, University of Edinburgh, Edinburgh, UK; 3 Institute of Health Informatics, University College London, London, UK

## Abstract

**Background:**

Both preserved ratio impaired spirometry (PRISm) (defined as a forced expiratory volume in one second (FEV1) <80% of predicted, while the ratio of FEV1 to forced vital capacity (FVC) is ≥0.7) and restrictive spirometry pattern (RSP) (defined as FVC<80% of predicted, while the FEV1/FVC ratio ≥0.7) are associated with an increased risk of mortality. The global prevalence of PRISm and RSP in the general population remains unclear. Therefore, we aimed to estimate the prevalence and identify risk factors of PRISm and RSP in the general population, and to examine variations across subgroups defined by gender, smoking status, WHO regions, and World Bank income levels.

**Methods:**

We searched three databases for studies that reported the prevalence of PRISm and RSP, and their associated risk factors in the general population. We conducted a multi-level meta-analysis, along with standard random-effects modelling, to estimate the pooled prevalence and identify key risk factors, and performed meta-regression and sensitivity analyses to assess the robustness of the results.

**Results:**

We identified a total of 57 studies reporting population-based data from 31 countries. We included 48 studies for meta-analysis using the Global Initiative for Chronic Obstructive Lung Disease (GOLD) definition, resulting in a pooled sample of 1 129 807 participants. The pooled prevalence of GOLD-PRISm was 10.60% (19 studies; 95% confidence interval (CI) = 8.12, 13.73), while the prevalence of GOLD-RSP was 12.09% (23 studies; 95% CI = 7.90, 18.04). The simultaneous combined prevalence of GOLD-PRISm and RSP was 11.79% (38 studies; 95% CI = 9.11, 15.12). Subgroup analysis showed that current smokers (13.37% *vs.* 10.18% in ex-smokers and 10.87% in non-smokers), and Western Pacific Region populations (11.26%) had higher prevalence rates of GOLD-PRISm. Significant risk factors for GOLD-PRISm include older adults, current and former smoking, extreme body mass index, and a history of comorbidities, such as asthma, diabetes, hypertension, and stroke.

**Conclusions:**

We provide a pooled estimate of PRISm and RSP prevalence based on studies from multiple regions, highlighting significant regional and demographic variations. Key risk factors, particularly smoking and comorbidities, should be considered when developing early management strategies.

Preserved ratio impaired spirometry (PRISm) is an unstable pulmonary condition characterised by a forced expiratory volume in one second (FEV1) of less than 80% predicted, while the ratio of FEV1 to forced vital capacity (FVC) remains above 0.7 [1]. Before the term PRISm was introduced in 2014, most prior studies focused on abnormalities in FVC, commonly referred to as the restrictive spirometry pattern (RSP), defined as an FVC below 80% along with a normal FEV1/FVC ratio [2]. Alternatively, the lower limit of normal (LLN) criterion defines PRISm as an FEV1 below the LLN with an FEV1/FVC ratio above the LLN, and RSP as an FVC below the LLN, while the FEV1/FVC ratio remains above the LLN.

Moreover, PRISm is associated with an increased risk of all-cause mortality, including respiratory-related hospitalisation and mortality [3]. Individuals with PRISm are at high risk for several adverse health outcomes, including cardiovascular disease, metabolic syndrome, and all-cause hospitalisation [3,4]. Recent studies indicate that individuals with PRISm are more likely to develop fixed airway obstruction compared to those with normal spirometry [5]. Moreover, PRISm plays a major role in impairing patients' quality of life, as heightened exertional dyspnoea, reduced exercise capacity, and accelerated progression of frailty have been observed among these individuals [6,7]. Despite the importance of PRISm/RSP as an early indicator of potential pulmonary disease, their global prevalence in the general population remains uncertain due to widely varying estimates across published studies.

The prevalence of RSP ranged from 8.4% in Canada to as high as 67.7% in India [8]. When the lower limit of normal (LLN) criteria was used, the RSP prevalence varied between 6.3% and 18.0% [9]. Following the introduction of PRISm, data from the UK Biobank, involving over 350 000 individuals, indicated that the prevalence of PRISm was nearly 11% [5], a figure consistent with other reports [10]. However, the prevalence of PRISm appears significantly lower in some regions. The Chinese Pulmonary Health study reported a PRISm prevalence of only 5.5% [11]. This rate aligns with findings from Japan but is much lower than those observed in European and North American countries [12]. Variations in prevalence may stem from differences in diagnostic criteria and risk factors. For example, current smokers were nearly seven times more likely to exhibit PRISm than non-smokers [13], and the prevalence among Chinese smokers reached 28.6% [14]. Despite some studies reviewing the epidemiology and pathophysiology of PRISm [15,16], a comprehensive and standardised assessment of its global prevalence in the general population is lacking, leaving significant gaps in our understanding of its worldwide burden.

Therefore, we conducted a systematic review and meta-analysis to estimate the global prevalence of PRISm/RSP and evaluate the influence of factors such as age, smoking status, and geographic region. This approach will offer valuable insights into prevalence patterns and associated risk factors for PRISm, enhancing our understanding of its epidemiology and public health burden.

## METHODS

### Study design

We conducted a systematic review and meta-analysis to determine the international prevalence of PRISm and RSP and to identify associated risk factors in the general population. We registered this review with PROSPERO (CRD42024563392) and followed the PRISMA guidelines in reporting our findings [17] (Appendix S1 in the **Online Supplementary Document**).

### Data sources and search strategy

We searched multiple databases, including MEDLINE, Embase, and Wanfang (Chinese databases), from their inception to 30 April 2025. Keywords included terms ‘preserved ratio impaired spirometry’, ’restrictive spirometry pattern’, ‘spirometry impairment’, and ‘lung function impairment’, among others. We used Boolean operators and truncation to optimise results (Appendix S2 in the **Online Supplementary Document**).

### Selection criteria

We included population-based studies conducted worldwide that recruited adults aged 18 years or older. To be eligible, studies had to define PRISm/RSP using spirometry measurements consistent with GOLD or LLN criteria and report prevalence estimates and/or odds ratios (ORs) related to risk factors of PRISm/RSP derived from univariable or multivariable analyses. We excluded studies not written in English or Mandarin, not based on population-level data, ineligible according to GOLD or LLN criteria for PRISm/RSP, those that failed to provide prevalence estimates, non-original publications, such as reviews, case reports, or opinion pieces.

### Data extraction and quality assessment

Three reviewers independently extracted data using a structured extraction form. Extracted information included study characteristics (*e.g.* publication year, sample size, study setting), definitions of PRISm and RSP (GOLD or LLN), and specific outcome data such as prevalence, case counts, and ORs for risk factors. Additionally, we extracted data on sex, smoking status, and geographic regions to enable subgroup analyses. The primary outcome included the global prevalence of PRISm, RSP, and combined PRISm/RSP in the general population, along with subgroup analyses, with prevalence data extracted as percentages. The secondary outcome involved the analysis of risk factors for PRISm/RSP. We extracted risk factor data as ORs with 95% confidence interval (CI). We calculated univariate ORs from studies where possible.

Two reviewers independently performed quality assessment (QA) using the Joanna Briggs Institute Checklist for Prevalence Studies [18]. We considered a score ≥7 as high quality. We resolved discrepancies regarding study inclusion, exclusion, data extraction, or quality assessment through consensus among the four reviewers (Appendix S3 in the **Online Supplementary Document**).

### Statistical analysis

We performed a meta-analysis to estimate pooled prevalence rates of PRISm/RSP and pooled ORs for potential risk factors. Specifically, we used multi-level meta-analytic (MLMA) models to predict pooled prevalence estimates, and calculated pooled ORs for risk factors with similar definitions using random-effects models. We ascertained the robustness of results using sensitivity analysis, including studies with quality assessment scores ≥7. We conducted additional sensitivity analysis by removing studies with exceptionally high or low prevalence (arbitrarily defined as prevalence outside 1st and 3rd quartiles among included studies) to identify the possible impact of outlier bias on pooled overall estimates. We reported heterogeneity using *I*^2^ and prediction intervals for all pooled estimates. Furthermore, we observed heterogeneity using univariable mixed-effect regression modelling. We reported effects (β) as differences in logit prevalence (proportions). Model performance was quantified using the Akaike information criterion and R^2^ values. For comparison, we reported results for multivariable meta-regression adjusting for all *a priori* selected variables in Table S1 in the **Online Supplementary Document**. We assessed publication bias visually using funnel plots and statistically using Egger’s regression tests. We conducted all analyses using *R*, version 4.5.0 (R Core Team, Vienna, Austria) statistical programming language [19–21].

## RESULTS

### Search results

We retrieved a total of 3127 studies from MEDLINE, Embase, and Wanfang searches, out of which we reviewed 275 full-text articles. After review, we included 57 studies, of which 48 were used in meta-analysis (Figure S1 in the **Online Supplementary Document**).

### Characteristics of included studies

Twenty-five studies reported on the prevalence of PRISm, 30 on the prevalence of RSP, and two on the prevalence of both PRISm and RSPFort ystudies used the GOLD definition for PRISm and RSP, nine studies used the LLN definition, and eight studies reported prevalence using both GOLD and LLN definitions.

The included studies covered a total of 31 countries spanning across four WHO regions: the WHO African Region (AFRO), the WHO Region of the Americas (AMRO), the WHO European Region (EURO), and the WHO Western Pacific Region (WPRO). Most studies were from the AMRO, and two studies reported prevalence from multiple settings. Among studies conducted in single locations, 42 studies were conducted in high-income countries (HICs), eight studies in upper-middle-income countries (UMICs), one study in a lower-middle-income country and two studies were conducted in low-income countries (LICs) (Figure S2 in the **Online Supplementary Document**). Quality assessment yielded a mean score of 7.82 for all studies included in the review (Table S2 in the **Online Supplementary Document**).

### Results of meta-analysis

We included 48 studies reporting PRISm and/or RSP according to GOLD criteria (referred to as GOLD-PRISm and GOLD-RSP hereafter) in the meta-analysis. We excluded nine studies that reported only LLN outcomes from the main analysis, and their meta-analysis is presented in the supplementary materials.

### Overall prevalence of PRISm and RSP

**Figure 1:**
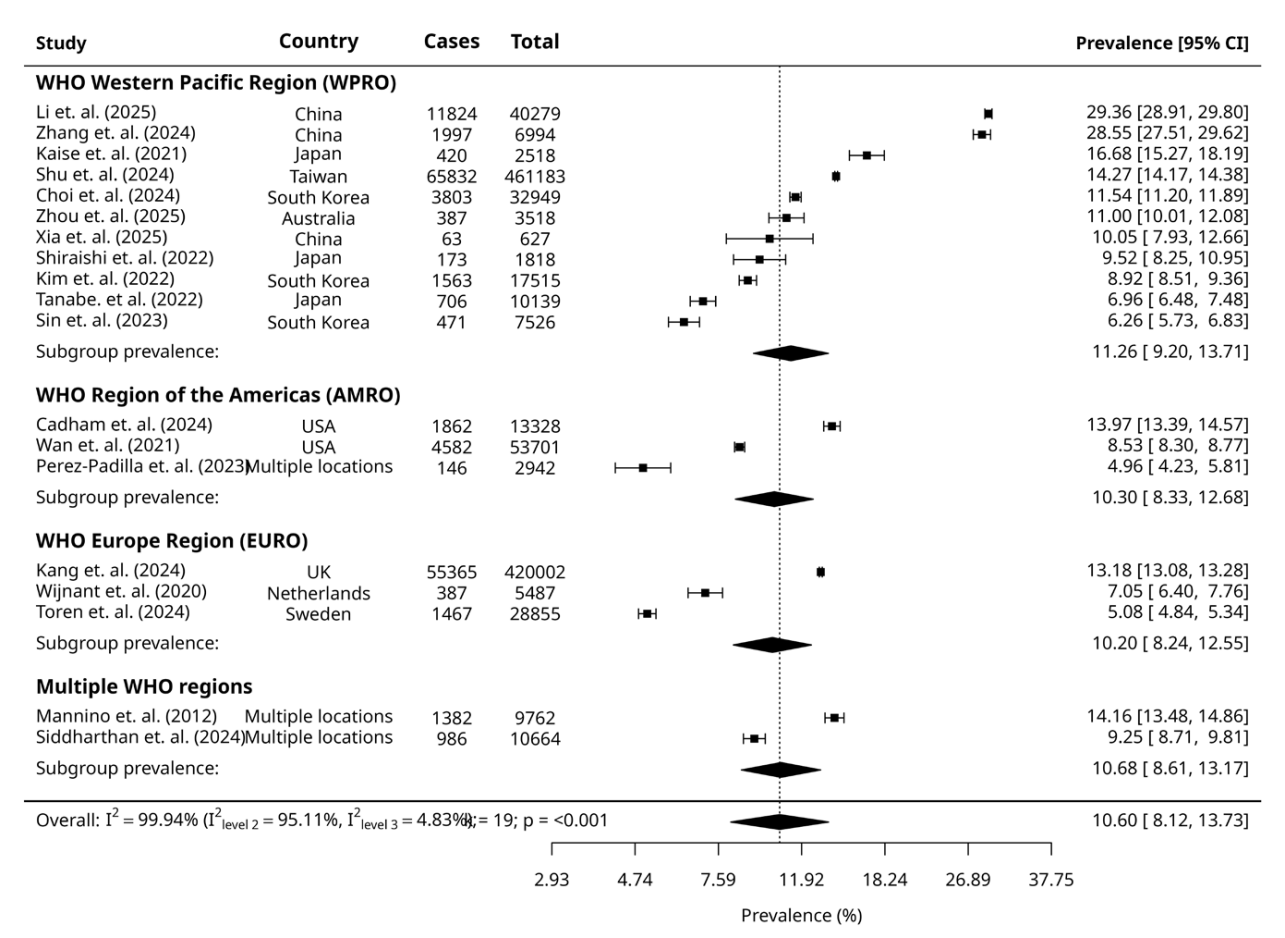
Forest plots of the included studies showing the estimated pooled prevalence of GOLD-PRISm.

**Figure 2:**
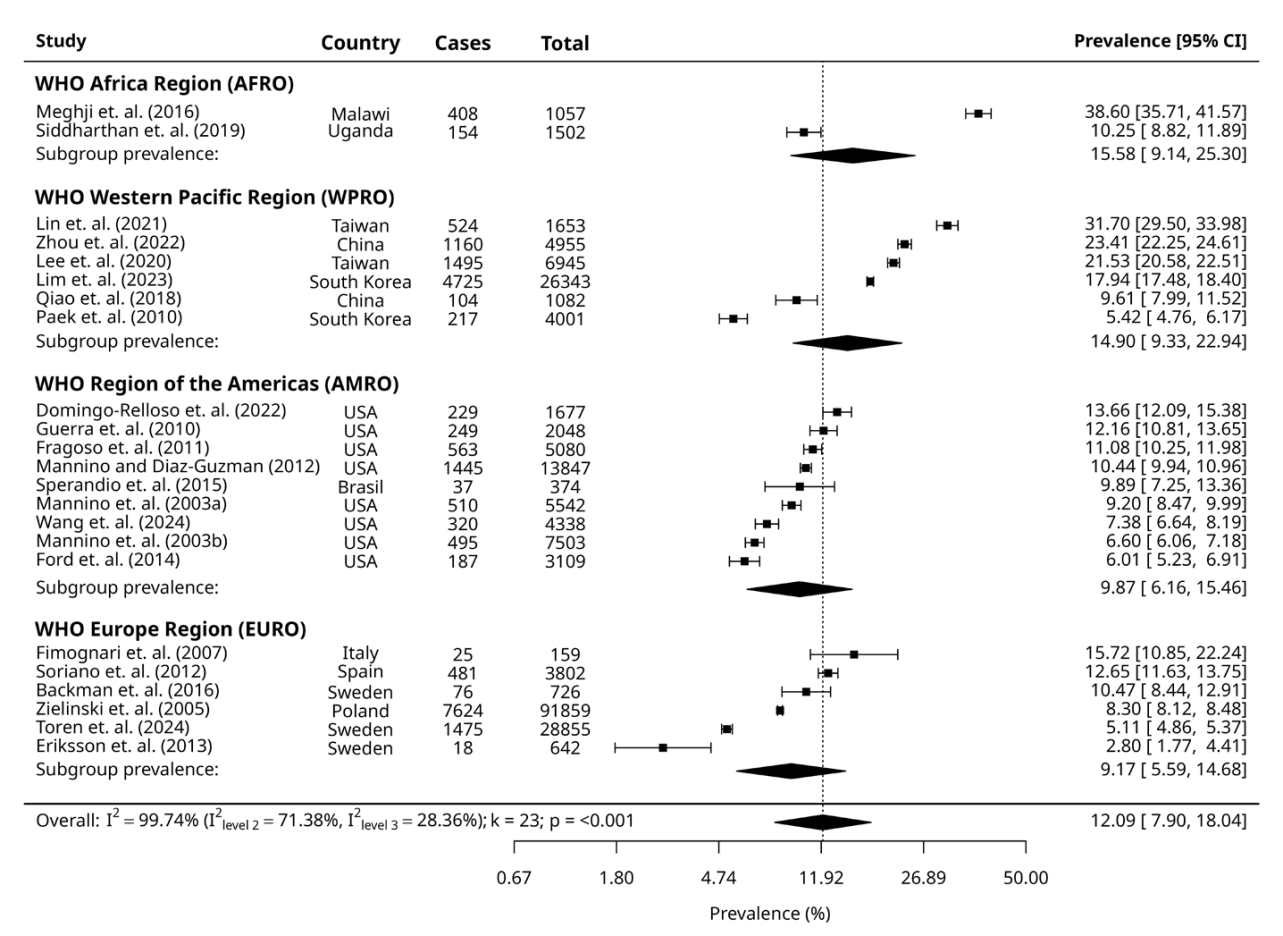
Forest plots of the included studies showing the estimated pooled prevalence of GOLD-RSP.

**Figure 3:**
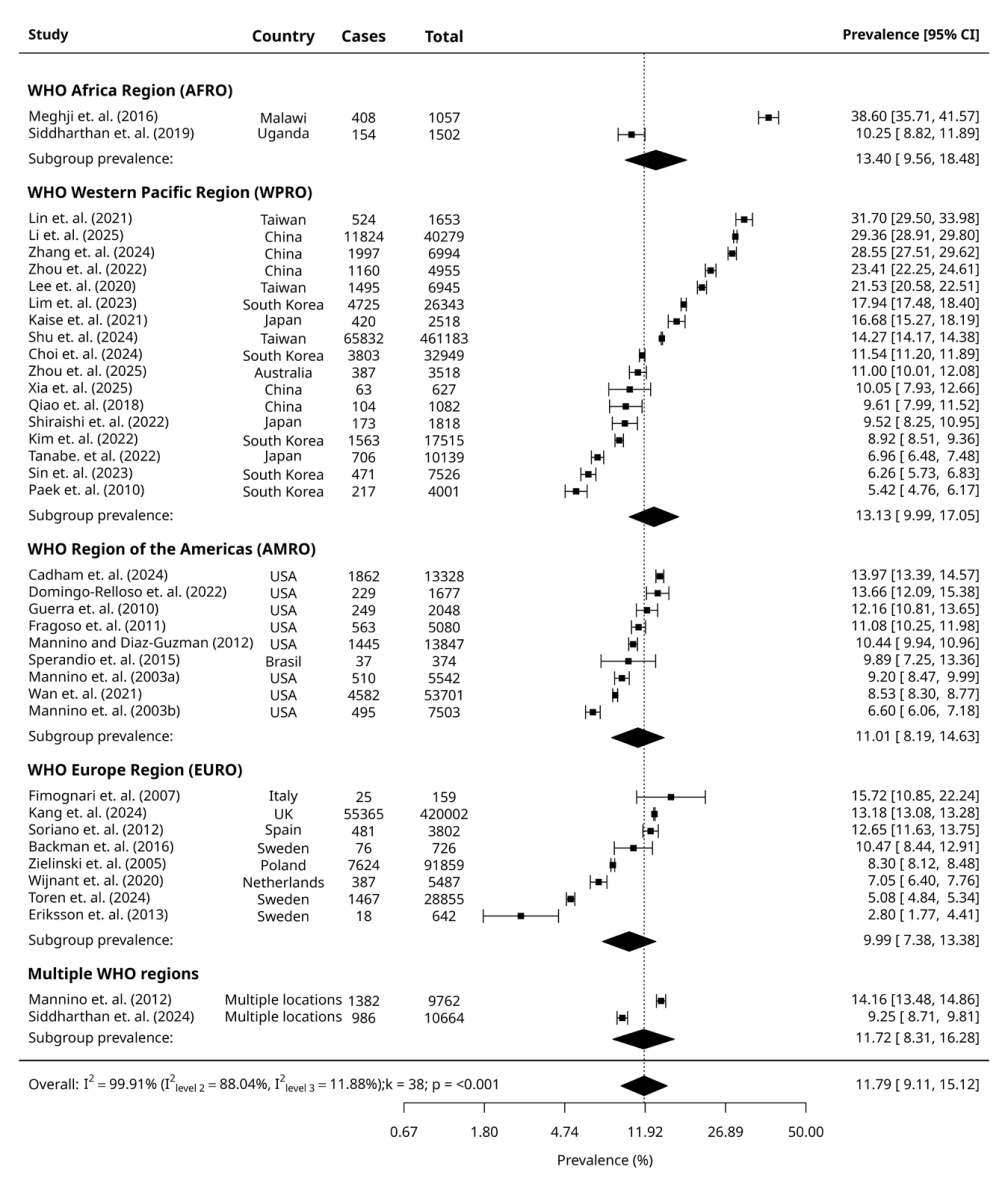
Forest plots of the included studies showing the estimated combined pooled prevalence of GOLD-PRISm and RSP.

We derived pooled prevalences of GOLD-PRISm, GOLD-RSP and combined GOLD-PRISm and GOLD-RSP from MLMA models, accounting for WHO geographical regions as the clustering variable. The overall pooled prevalence of GOLD-PRISm was 10.60% (95% CI = 8.12, 13.73) in our pooled sample of 1 129 807 participants (**Figure 1**). The overall pooled prevalence of GOLD-RSP was 12.09% (95% CI = 7.90, 18.04) for a pooled sample of 217 099 participants (**Figure 2**). Overall, the combined GOLD-PRISM and RSP pooled prevalence was 11.79% (95% CI = 9.11, 15.12) for a pooled sample of 1 307 662 participants (**Figure 3**). We reported consolidated overall prevalences from all MLMA and random-effect models and forest plots reported in Table S3 and Figure S3 in the **Online Supplementary Document**.

### Subgroup prevalence

Random-effects models reported similar prevalences of GOLD-PRISm and GOLD-RSP between males and females, respectively. The prevalence of GOLD-PRISm was highest among current smokers compared to non-smokers and ex-smokers. In contrast, the prevalence of GOLD-RSP was similar between non-smokers and current smokers, followed by ex-smokers. For both outcomes, prevalence was lowest among ex-smokers. We derived prevalences within WHO geographical regions and World Bank income-levels from their respective MLMA models. Prevalence of GOLD-PRISm was highest in WPRO, followed by the AMRO and EURO, and the prevalence of GOLD-RSP was highest in the AFRO, followed by the WPRO, the AMRO, and the lowest in the EURO. UMICs had the highest prevalence of GOLD-PRISm. Prevalence of GOLD-RSP was highest in LICs, followed by UMICs. Prevalence of GOLD-PRISm and GOLD-RSP was lowest among HICs (**Table 1**).

**Table 1:** Sub-group meta-analysis for prevalence of GOLD-PRISm, GOLD-RSP and combined GOLD-PRISm and RSP

	GOLD-PRISm					GOLD-RSP					Combined GOLD-PRISm and GOLD-RSP				
	**k**	***I*^2^ (%)**	***P*-value**	**Prevalence (%, 95% CI)**	**95% PI**	**k**	***I*^2^ (%)**	***P*-value**	**Prevalence (%, 95% CI)**	**95% PI**	**k**	***I*^2^ (%)**	***P*-value**	**Prevalence (%, 95% CI)**	**95% PI**
**Prevalence by gender (random-effects model)**															
Male	18†	99.8	<0.001	10.24 (7.62, 13.63)	2.45, 34.15	16†	99.4	<0.001	8.88 (6.9, 11.37)	2.90, 24.12	33†	99.7	<0.001	9.88 (8.15, 11.93)	3.03, 27.80
Female	17	98.8	<0.001	10.45 (8.11, 13.36)	3.18, 29.34	15	98.9	<0.001	9.73 (7.32, 12.83)	2.78, 28.89	32	99.7	<0.001	10.12 (8.41, 12.14)	3.28, 27.23
**Prevalence by smoking status (random-effects model)**															
Non-smoker	17	99.8	<0.001	10.87 (8.44, 13.89)	3.29, 30.41	8	98.1	<0.001	8.39 (6.03, 11.57)	2.54, 24.37	25	99.8	<0.001	10.02 (8.16, 12.23)	3.27, 26.85
Ex-smoker	17	99.2	<0.001	10.18 (7.76, 13.24)	2.83, 30.58	8	98.4	<0.001	7.87 (5.35, 11.45)	1.98, 26.58	25	99.1	<0.001	9.40 (7.53, 11.68)	2.81, 27.16
Current smoker	20	99.4	<0.001	13.23 (10.43, 16.64)	3.97, 35.99	9	93.2	<0.001	8.04 (6.49, 9.92)	3.76, 16.35	29	99.2	<0.001	11.33 (9.28, 13.76)	3.53, 30.84
**Prevalence by WHO geographical region (MLMA model)**															
AFRO		99.9	<0.001			2	99.7	<0.001	15.58 (9.14-25.30)	6.52, 32.83‡	2	99.9	<0.001	13.40 (9.56, 18.48)	8.04, 21.49‡
WPRO	11			11.26 (9.20, 13.71)	8.32, 15.06	6			14.90 (9.33, 22.94)	6.47, 30.70	17			13.13 (9.99, 17.05)	8.21, 20.33
AMRO	3			10.30 (8.33, 12.68)	7.55, 13.91‡	9			9.87 (6.16, 15.46)	4.19, 21.53	9			11.01 (8.19, 14.63)	6.76, 17.43
EURO	3			10.20 (8.24, 12.55)	7.47, 13.77‡	6			9.17 (5.59, 14.68)	3.83, 20.37	8			9.99 (7.38, 13.38)	6.08, 15.97
Multiple regions	2			10.68 (8.61, 13.17)	7.82, 14.43‡						2			11.72 (8.31, 16.28)	6.98, 19.02‡
**Prevalence by World Bank income-level (MLMA model)§**															
LIC		99.9	<0.001			2	99.7	<0.001	14.22 (9.41, 20.92)	7.62, 25.01‡	2	99.9	<0.001	15.87 (10.44, 23.41)	8.22, 28.45‡
UMIC	3			18.65 (10.58, 30.75)	6.69, 42.30‡	3			12.77 (8.5, 18.74)	6.84, 22.60‡	6			15.56 (10.71, 22.07)	8.29, 27.31
HIC	13			9.87 (5.55, 16.94)	3.38, 25.51	18			10.75 (7.26, 15.63)	5.78, 19.13	28			10.87 (7.59, 15.33)	5.75, 19.59
Mixed income groups	3			9.51 (5.15, 16.91)	3.18, 25.15‡						2			12.90 (8.38, 19.33)	6.57-23.77*

AFRO – WHO African Region, AMRO – WHO Region of the Americas, CI – confidence interval, EMRO – WHO Eastern Mediterranean Region, EURO – WHO European Region, GOLD – Global Initiative for Obstructive Lung Disease, HIC – high-income country, LIC – low-income country, MLMA – multi-level meta-analysis, PI – prediction interval, PRISm – preserved ratio impaired spirometry, RSP – restrictive spirometric pattern, UMIC – Upper-middle income country, and WPRO – WHO Western Pacific Region

*We excluded four studies (1–4) from the combined prevalence meta-analysis due to data duplication.

†Cestelli *et al.* (2025) recruited only men in their study. Both GOLD-PRISm and GOLD-RSP outcomes were reported for males. However, this study, was removed from overall prevalence calculations as it did not represent the general population.

‡Prediction interval estimates may be imprecise if fewer studies are included in the meta-analysis. Caution is advised when interpreting this estimate.

^§^Countries were classified according to World Bank country classification by income level for 2024–2025.

### Risk factors

Meta-analysis of univariable estimates suggested certain risk factors for GOLD-PRISm. Among 40–49 (reference group), 50–59, 60–69, and 70–79-year age groups, we associated 50–59 and 70–79-year groups with higher odds of GOLD-PRISm. Furthermore, we also associated current and former smoking with statistically higher odds of GOLD-PRISm. Comorbidities, including a history of asthma, diabetes, hypertension, obesity (body mass index (BMI)>30 kg/m2), and stroke, were significantly associated with higher odds of GOLD-PRISm. Meta-analyses of multivariable ORs reported significantly higher odds of GOLD-PRISm in participants with a history of diabetes or hypertension (**Table 2**).

**Table 2:** Pooled estimates for risk factors of GOLD-PRISm and GOLD-RSP*

		GOLD-PRISm					GOLD-RSP				
**Risk factor**	**Reference group**	**k**	***I*^2^ (%)**	***P*-valaue**	**Pooled OR (95% CI)**	**95% PI**	**k**	***P*-valaue**	***I*^2^ (%)**	**Pooled OR (95% CI)**	**95% PI**
**Univariable OR**											
Age 50 to 59 years	40 to 49 years	6	45.4	<0.001	1.16 (1.06, 1.27)	0.97, 1.40					
Age 60 to 69 years	40 to 49 years	6	81.7	0.187	1.14 (0.94, 1.38)	0.62, 2.10					
Age 70 to 79 years	40 to 49 years	3	39.6	0.011	1.25 (1.05, 1.48)	0.70, 2.23†					
Gender - male	female	19	99.9	0.108	1.20 (0.96, 1.51)	0.42, 3.49	12	0.517	90.9	1.08 (0.85, 1.38)	0.46, 2.55
Current smoking	Never smoking	22	99.0	<0.001	1.36 (1.14, 1.62)	0.57, 3.25	10	0.030	79.6	1.27 (1.02, 1.57)	0.63, 2.56
Former smoking	Never smoking	16	91.3	0.024	1.15 (1.02, 1.30)	0.69, 1.92	9	0.647	90.7	1.05 (0.86, 1.29)	0.54, 2.03
Obesity (>30 kg/m^2^)	18.5–24.9 kg/m^2^	7	96.5	<0.001	1.81 (1.48, 2.21)	0.95, 3.43	6	<0.001	68.8	2.21 (1.75, 2.78)	1.14, 4.29
Overweight (25–29.9 kg/m^2^)	18.5–24.9 kg/m^2^	6	98.8	0.202	1.17 (0.92, 1.48)	0.54, 2.52	6	0.304	77.5	1.15 (0.88, 1.49)	0.51, 2.57
Underweight (<18.5 kg/m^2^)	18.5–24.9 kg/m^2^	6	94.9	0.049	1.76 (1.00, 3.09)	0.27, 11.37	3	0.025	89.2	2.68 (1.13, 6.35)	0.09, 82.23†
History of asthma	No history of asthma	11	89.9	<0.001	1.81 (1.61, 2.02)	1.25, 2.62					
History of diabetes	No history of diabetes	13	98.2	<0.001	2.09 (1.65, 2.64)	0.83, 5.29	5	<0.001	38.0	2.64 (2.13, 3.26)	1.61, 4.32
History of hypertension	No history of hypertension	11	99.6	<0.001	1.65 (1.32, 2.06)	0.72, 3.80	3	<0.001	0.0	2.08 (1.46, 2.95)	0.96, 4.48
History of CVD	No history of CVD	4	99.1	0.336	1.33 (0.74, 2.38)	0.17, 10.12					
History of stroke	No history of stroke	5	64.5	<0.001	2.09 (1.71, 2.55)	1.21, 3.60					
History of tuberculosis	No history of tuberculosis	3	95.3	0.374	2.28 (0.37, 14.11)	0.00, 5805.51†					
**Univariable OR**											
Gender - male	female						3	<0.001	45.3	1.74 (1.30, 2.31)	0.86, 3.50†
Current smoking	Never smoking	4	94.6	0.349	1.21 (0.81, 1.79)	0.30, 4.90	4	0.043	49.9	1.55 (1.01, 2.37)	0.52, 4.61
Former smoking	Never smoking	3	0.0	0.764	0.99 (0.95, 1.04)	0.90, 1.10†					
Obesity (>30 kg/m2)	18.5–24.9 kg/m2						3	<0.001	44.5	1.97 (1.34, 2.89)	0.53, 7.27†
History of diabetes	No history of diabetes	5	87.7	<0.001	1.67 (1.24, 2.34)	0.63, 4.38	3	<0.001	0.0	2.06 (1.77, 2.39)	1.48, 2.86†
History of hypertension	No history of hypertension	3	71.6	0.002	1.42 (1.14, 1.77)	0.58, 3.45†					

Note: CI – confidence interval, BMI – body mass index, GOLD – Global Initiative for Obstructive Lung Disease, OR – odds ratio, PI – prediction interval, PRISm – preserved ratio impaired spirometry, RSP – restrictive spirometric pattern

*An estimate is statistically significant if its 95% CI does not contain the null value (1.00).

†Prediction interval estimates may be imprecise if fewer studies are included in the meta-analysis. Caution is advised when interpreting this estimation.

We found current smoking, obesity, being underweight (BMI<18.5 kg/m2), history of diabetes and hypertension to be significant risk factors for GOLD-RSP in the univariate OR meta-analysis. Male gender was not significantly associated with increased odds of GOLD-RSP in univariable analysis; however, it was significantly associated in the meta-analysis of multivariable ORs. A possible explanation for this dichotomy could be explained by the difference in the number of studies reporting univariable (k = 12) *vs.* multivariable (k = 3) ORs. In the meta-analysis of multivariable effect estimates, male gender, current smoking, obesity, and history of diabetes were significant risk factors for GOLD-RSP (**Table 2**). Forest plots for risk factor meta-analysis have been provided in Figure S4 in the **Online Supplementary Document**.

### Exploration of heterogeneity using meta-regression

Univariate mixed-effect meta-regression of *a priori* selected moderators showed that the prevalence of GOLD-PRISm was significantly associated with World Bank income-level (*P* = 0.01) and mean age of study population (*P* = 0.05). The final adjusted model explained 34.76% (R^2^) of total heterogeneity, and only World Bank income-level was significant in the multivariable model (**Table 3**). Only geographical regions were a major cause of heterogeneity in GOLD-RSP prevalence among variables included in our analysis. Among them, only the EURO (*P* = 0.03) region was a significant moderator. The final adjusted regression model for GOLD-RSP explained 71.31% of total heterogeneity (R^2^). Both AMRO (*P* = 0.002) and EURO regions (*P* = 0.001) were significant moderators (**Table 3**).

**Table 3:** Results of mixed-effect meta-regression – overall GOLD-PRISm and GOLD-RSP prevalence

	GOLD-PRISm			GOLD-RSP		
**Variable**	*β* (95% CI)*	***P*-value**	**R^2^ (%)**	*β* (95% CI)*	***P*-value**	**R^2^ (%)**
**Univariable meta-regression**						
WHO geographical location (reference: WPRO)			0.00			21.00
*AFRO*				0.33 (−0.68, 1.33)	0.52	
*AMRO*	−0.42 (−1.16, 0.32)	0.27		−0.64 (−1.28, 0.01)	0.053	
*EURO*	−0.54 (−1.28, 0.20)	0.15		−0.79 (−1.50, −0.07)	0.03	
*Multi*	−0.13 (−1.00, 0.74)	0.77				
World Bank income-level (reference: HIC)			30.85			5.34
*LIC*				0.87 (−0.14, 1.87)	0.09	
*UMIC*	0.91 (0.31, 1.52)	0.01		0.33 (−0.52, 1.17)	0.45	
*Mixed*	−0.13 (−0.73, 0.47)	0.67				
Bronchodilator use (reference: No)	−0.43 (−1.00, 0.14)	0.14	6.36	0.09 (−0.62, 0.81)	0.80	0.00
Mean age	−0.04 (−0.08, 0.00)	0.05	14.94	0.01 (−0.02, 0.05)	0.43	0.00
Females in study population in %	−0.01 (−0.07, 0.05)	0.76	0.00	0.01 (−0.02, 0.04)	0.55	0.00
Current smokers in study population in %	0.01 (−0.03, 0.04)	0.73	0.00	0.00 (−0.02, 0.02)	0.87	0.00
Publication year	0.01 (−0.08, 0.1)	0.81	0.00	0.03 (−0.01, 0.07)	0.17	4.25
**Multivariable meta-regression (best-fit models)**						
WHO geographical location (reference WPRO)†			34.76			71.31
*AFRO*				1.32 (−0.03, 2.66)		
*AMRO*				−1.41 (−2.33, −0.48)		
*EURO*				−1.64 (−2.61, −0.66)		
*Multi*						
World Bank income-level (reference: HIC)						
LIC						
UMIC	0.88 (0.20, 1.55)	0.01				
Mixed	−0.25 (−0.91, 0.41)	0.46				
Bronchodilator use (reference: no)				−0.52 (−1.38, 0.34)	0.23	
Mean age	−0.03 (−0.07, 0.01)	0.19		0.03 (0.00, 0.06)	0.07	
Females in study population in %						
Current smokers in study population in %				0.01 (0.00, 0.01)	0.30	
Publication year	−0.06 (−0.15, 0.03)	0.20				

EMRO – Eastern Mediterranean Region, EURO – European Region, GOLD – Global Initiative for Obstructive Lung Disease, HIC – high-income country, LIC – low-income country, PRISm – preserved ratio impaired spirometry, RSP – restrictive spirometric pattern, UMIC – upper-middle income country, WHO – World Health Organization, WPRO – Western Pacific Region

*Logit proportion.

†Countries were classified according to World Bank country classification by income level for 2024–2025 [42].

### Sensitivity analyses

The prevalence of GOLD-PRISm was similar in the sensitivity analysis compared to the main meta-analysis. However, the prevalence of GOLD-RSP was lower in high-quality studies (QA score ≥7). For outlier bias, we observed a slight reduction in heterogeneity (*I*^2^) after removing outliers, leading us to interpret that our findings were not biased due to outliers. Results of sensitivity analysis have been reported in Table S11 in the **Online Supplementary Document**.

### Studies excluded from the main meta-analysis

Nine studies reported prevalence of PRISm and RSP using only LLN-based spirometry definitions, and eight studies reported prevalence using both GOLD and LLN definitions. The prevalence of LLN-PRISm was 7.63%. and prevalence of LLN-RSP was 6.76% (Tables S12 and S13 in the **Online Supplementary Document**).

### Publication bias

We observed slight asymmetry in funnel plots of the overall prevalence of GOLD-PRISm, GOLD-RSP and combined prevalence, suggesting potentially missing smaller studies that report lower prevalence. We found all results from Egger’s tests non-significant, however, indicating no strong statistical evidence of publication bias (S14 in the **Online Supplementary Document**).

## DISCUSSION

In this systematic review, we included 48 studies and estimated that the pooled prevalence of PRISm and RSP in the general population aged 18 years and older was approximately 10.60% and 12.09%, respectively, based on the GOLD criteria. When we combined PRISm and RSP, the overall prevalence was 11.79% using the GOLD definition. We found that PRISm prevalence was notably higher, at 13%, among current smokers. Key risk factors for PRISm identified in this review included older adults, smoking, extremes of BMI, and a history of comorbidities, including asthma, diabetes, hypertension, and stroke.

A recent systematic review found a pooled prevalence of PRISm at 12% among community members, inpatients and individuals undergoing health check-ups [22]. We included only studies focusing on the general population and distinguished prevalence estimates based on both the GOLD and LLN definitions. Our pooled global prevalence of PRISm is approximately 1.4% lower than their report under the GOLD criteria. A possible explanation for this is that we excluded hospital-based studies and specific populations [23–25] who may have had higher exposure to cigarettes and other risk factors, potentially introducing selection bias. Our findings offer a more precise estimate for the general population, contributing valuable insights into the epidemiology and clinical significance of these pulmonary function abnormalities.

The reported prevalence of PRISm and RSP varied from 1.4% to 28.6% [12,26–28] across different studies. Several factors may account for the variation, with population heterogeneity being the primary likely source. For instance, populations with a higher prevalence of smoking, as demonstrated in the COPDgene study, tend to exhibit higher levels of PRISm [1]. Second, 48 of the 57 studies utilised the GOLD definition, which tends to yield higher prevalence compared to the LLN criteria [29]. We calculated prevalence using both criteria independently and confirmed that the prevalence of both PRISm and RSP is higher when applying the GOLD definition, as it is less strict than the LLN definition, particularly in older adults. This finding aligns with observations in COPD studies [30], where the GOLD definition is known to overestimate prevalence, particularly among older individuals. Third, the reference equations used in a study can influence both GOLD and LLN-based definitions. For instance, a study conducted in Malawi reported a GOLD-defined RSP prevalence of 9% using locally derived reference equations, compared to 38.6% when using NHANES equations [31]. Fourth, we found that WHO regions and World Bank income levels were associated with variations in prevalence, suggesting that geographic location, cultural factors, healthcare availability, lifestyle, and the quality of chronic disease management may play important roles in the development of these abnormal spirometric patterns.

The definition of PRISm and RSP emphasise different aspects of spirometric parameters, with PRISm primarily characterised by reduced FEV1, whereas RSP is defined by reduced FVC [1]. In some earlier studies, researchers also used FEV1 less than 80% predicted to define RSP. A recent retrospective study revealed that the two definitions showed over 70% overlap [32]. Both PRISm and RSP share similar risk factors, including smoking, obesity, and diabetes, and exhibit comparable mortality rates in patients with metabolic or cardiovascular conditions [33]. Given these similarities, we combined the two definitions in the present systematic review to calculate the overall prevalence. We found that the prevalence of PRISm was comparable to that of RSP, which aligns with recent reports showing a similar morbidity and mortality between the two definitions [28,32]. These findings further support the similarity between PRISm and RSP in the general populations.

We observed that the prevalence of PRISm was about 2.5% higher in current smokers than in non-smokers when using the GOLD definition. Persistent exposure to cigarette smoke is known to accelerate lung tissue ageing, reduce elastic recoil, and induce mucus hypersecretion, which in turn can impair lung function and contribute to the development of PRISm or RSP. Recent studies have also confirmed that individuals with PRISm are at a higher risk of respiratory symptoms, small airway dysfunction, and air trapping, consistent with the potential pathophysiological effects of smoking on the lungs [34,35]. However, this discrepancy in prevalence across smoking status was not observed for RSP, possibly because RSP is more closely associated with socioeconomic status, early-life lung development, and exposure to environmental hazards. Interestingly, the similar prevalences of PRISm and RSP between non-smokers and ex-smokers suggest that smoking cessation may be associated with a reduced prevalence of PRISm/RSP among individuals with a history of cigarette exposure, although causal inference cannot be established.

We identified obesity as a significant risk factor for both PRISm and RSP, and being underweight as a significant risk factor for RSP. Underweight individuals may experience weakened respiratory accessory muscles and reduced forced expiratory function, whereas obese individuals, particularly those with elevated waist-to-hip ratios, may face restricted expiration due to mechanical limitations [36,37]. These mechanisms likely underpin the metabolic subtype of PRISm [1]. We identified hypertension and cardiovascular disease (stroke) as significant risk factors for PRISm. It has been hypothesised that individuals with cardiovascular diseases may have an increased likelihood of subclinical heart failure, leading to pulmonary congestion and subsequently restricted spirometry [4]. However, this phenomenon is less likely to manifest in younger, otherwise healthy populations. An alternative explanation for the observed association between stroke and PRISm could be confounding factors, such as smoking and metabolic abnormalities. To clarify these relationships, future prospective cohort studies incorporating concurrent echocardiograms and chest CT imaging are necessary.

Notably, our meta-analysis was characterized by high heterogeneity, bringing into question the accuracy of estimates. To better understand the source of this heterogeneity, we built meta-regression models which explained more than one-third of the total heterogeneity for GOLD-PRISm prevalence, and more than two-thirds of the total heterogeneity for GOLD-RSP prevalence calculations. We used socioeconomic and geographical variation as the main sources of population heterogeneity for PRISm and RSP prevalences, respectively. This epidemiological behaviour is expected and similar to other respiratory conditions, as these have certain common geographic and socioeconomic predisposing factors such as air quality, climate, lifestyle factors, and smoking prevalence. In addition to expected sources of heterogeneity, we would also like to state certain methodological caveats concerning meta-analyses of prevalence. High *I*^2^ values are relatively common for prevalence-based meta-analysis, with Migliavaca *et al.* reporting a median *I*^2^ of 96.9% in their meta-analysis of 134 prevalence studies. The authors propose certain possible reasons for this, including temporal and geographical variation in prevalence trends, large sample sizes of prevalence studies leading to precise estimates (which result in narrower CIs and subsequently, lesser overlap and higher heterogeneity), and finally, the non-comparative nature of proportions, which results in more diverse estimates than comparative data such as relative risks, and ORs [38].

The present systematic analysis has some limitations. First, most of the included studies were conducted in high- and middle-income countries, which may introduce selection bias, while generalising the estimates of prevalence of PRISm/RSP to less developed regions. For example, PRISm/RSP prevalence was reported to be significantly higher in Malawi and South America compared to other countries [6,31]. Therefore, population-based studies specifically designed to evaluate regional and socioeconomic effect modification and to disentangle biological and environmental mechanisms are needed in the future. Second, random sampling was performed in only a limited number of studies. As PRISm is typically asymptomatic, individuals without symptoms may be less likely to undergo spirometry during cross-sectional screenings, potentially leading to an underestimation of PRISm/RSP prevalence. For instance, a recent nationally representative cross-sectional study that utilised randomised stratified sampling reported a PRISm prevalence of 5.5% [11], which is considerably lower than previously reported estimates in China [9]. Third, fewer than 20% of the studies included utilised post-bronchodilator spirometry to screen for PRISm/RSP. This may have led to an overestimation of prevalence, particularly for PRISm, as FEV1 values are more responsive to bronchodilator use [39,40]. Finally, our risk factor estimates might be affected by certain weaknesses which could reduce reliability. Certain comorbidities, such as hypertension and asthma, were primarily self-reported by patients in some studies, which could introduce bias in the assessment of risk factors [5]. Moreover, since we derived most data from unadjusted estimates, there is a possible risk of confounding. These factors encourage a degree of caution when interpreting risk factor results.

PRISm is considered a pre-disease state of COPD, with nearly 30% of individuals progressing to COPD over a 5-year follow-up period [41]. The high prevalence of PRISm in the general population, as indicated by our analysis, underscores the need for greater attention to this group of individuals. Furthermore, the potential reversibility of PRISm to normal spirometry suggests that it represents a critical window for early intervention and prevention of COPD [5]. Our systematic analysis highlights the need for more data from Africa, the Eastern Mediterranean, and the Southeast Asia region to ensure a globally representative estimate of PRISm/RSP prevalence. Additionally, the adoption of standardised diagnostic criteria and the inclusion of post-bronchodilator spirometry in future research would help reduce heterogeneity across studies and improve the reliability of prevalence estimates.

## CONCLUSIONS

Our findings suggest a high prevalence of PRISm/RSP, with PRISm being particularly common among current smokers. Identified risk factors include active smoking, extreme BMI, and comorbidities such as asthma, diabetes, obesity, hypertension, and stroke. These findings highlight the substantial global burden of PRISm and RSP and underscore the importance of targeted screening and early identification in high-risk populations. Increased awareness of modifiable risk factors, particularly smoking and abnormal BMI, may facilitate preventive strategies and inform public health interventions.

## Additional material


Online Supplementary Document
