# Supplementary material for: Prevalence of preserved ratio impaired spirometry and restrictive spirometry pattern in the general population: a systematic review and multi-level meta-analysis of studies from multiple countries
Source: J Glob Health. 2026 Mar 27;16:04072. doi: 10.7189/jogh.16.04072 (PMC13023682; doi:10.7189/jogh.16.04072)
Supplement: Online Supplementary Document [file jogh-16-04072-s001.pdf]

Supplement to: Xu W, Ferdous S, Peng B, Shi T. Prevalence of preserved ratio impaired spirometry and restrictive spirometry pattern in the general population: a systematic review and multi-level meta-analysis of studies from multiple countries. J Glob Health, 2026;16:04072.

Table of contents

Table of contents 2

Supplement S01: PRISMA 2020 checklist 3

Supplement S02: Search strategy 6

Supplement S03: Quality assessment 10

Supplement S04: Mixed-effect meta-regression – Multivariable models adjusted for all moderators (GOLD analysis) 13

Supplement S05: PRISMA flowchart showing study inclusion process 14

Supplement S06: Map showing global distribution of included studies 15

Supplement S07: Baseline characteristics of included studies (k=57) 16

Supplement S08: Consolidated overall prevalences from multi-level meta-analysis and random-effects models (main analysis) 24

Supplement S09: Forest plots – GOLD studies (main analysis) 25

Supplement S10: Forest plots – Risk factors (main and supplementary analysis) 38

  10.1 GOLD studies (main analysis) 38

  10.2 LLN studies (supplementary analysis) 52

Supplement S11:Sensitivity analysis - Pooled prevalences of GOLD-PRISm, GOLD-RSP, and combined GOLD-PRISm and GOLD-RSP in subgroups (QA score >7) 56

Supplement S12: Supplementary analysis - Results of overall LLN-PRISm, LLN-RSP, combined LLN-PRISm and RSP, sub-group meta-analyses and meta-regression 59

Supplement S13: Forest plots – LLN studies (supplementary analysis) 62

Supplement S14: Tests to investigate publication bias 71

  14.1 Overall prevalence of GOLD-PRISm 71

  14.2 Overall prevalence of GOLD-RSP 72

  14.3 Overall prevalence of combined GOLD-PRISm and GOLD-RSP 73

References 74

Supplement S01: PRISMA 2020 checklist

Section and Topic	Item #	Checklist item	Location where item is reported
**TITLE**			
Title	1	Identify the report as a systematic review.	Page 2, line 32
**ABSTRACT**			
Abstract	2	See the PRISMA 2020 for Abstracts checklist.	-
**INTRODUCTION**			
Rationale	3	Describe the rationale for the review in the context of existing knowledge.	Page 6, line 83-102
Objectives	4	Provide an explicit statement of the objective(s) or question(s) the review addresses.	Page 7, line 103-106
**METHODS**			
Eligibility criteria	5	Specify the inclusion and exclusion criteria for the review and how studies were grouped for the syntheses.	Page 8, line 122-128
Information sources	6	Specify all databases, registers, websites, organisations, reference lists and other sources searched or consulted to identify studies. Specify the date when each source was last searched or consulted.	Page 8, line 116-120
Search strategy	7	Present the full search strategies for all databases, registers and websites, including any filters and limits used.	Supplementary materials(S02)
Selection process	8	Specify the methods used to decide whether a study met the inclusion criteria of the review, including how many reviewers screened each record and each report retrieved, whether they worked independently, and if applicable, details of automation tools used in the process.	Page 8, line 130
Data collection process	9	Specify the methods used to collect data from reports, including how many reviewers collected data from each report, whether they worked independently, any processes for obtaining or confirming data from study investigators, and if applicable, details of automation tools used in the process.	Page 8-9, line 130-135
Data items	10a	List and define all outcomes for which data were sought. Specify whether all results that were compatible with each outcome domain in each study were sought (e.g. for all measures, time points, analyses), and if not, the methods used to decide which results to collect.	Page 9, line 134
	10b	List and define all other variables for which data were sought (e.g. participant and intervention characteristics, funding sources). Describe any assumptions made about any missing or unclear information.	Page 9, line 133-134
Study risk of bias assessment	11	Specify the methods used to assess risk of bias in the included studies, including details of the tool(s) used, how many reviewers assessed each study and whether they worked independently, and if applicable, details of automation tools used in the process.	Page 9 line 139-142
Effect measures	12	Specify for each outcome the effect measure(s) (e.g. risk ratio, mean difference) used in the synthesis or presentation of results.	Page 9, line 135-138
Synthesis methods	13a	Describe the processes used to decide which studies were eligible for each synthesis (e.g. tabulating the study intervention characteristics and comparing against the planned groups for each synthesis (item #5)).	
	13b	Describe any methods required to prepare the data for presentation or synthesis, such as handling of missing summary statistics, or data conversions.	Page 9, line 137-138
	13c	Describe any methods used to tabulate or visually display results of individual studies and syntheses.	-
	13d	Describe any methods used to synthesize results and provide a rationale for the choice(s). If meta-analysis was performed, describe the model(s), method(s) to identify the presence and extent of statistical heterogeneity, and software package(s) used.	Page 9-10, line 144-157
	13e	Describe any methods used to explore possible causes of heterogeneity among study results (e.g. subgroup analysis, meta-regression).	Page 10, line 151-155
	13f	Describe any sensitivity analyses conducted to assess robustness of the synthesized results.	Page 10, 147-150
Reporting bias assessment	14	Describe any methods used to assess risk of bias due to missing results in a synthesis (arising from reporting biases).	Page 10, line 155-157

Section and Topic	Item #	Checklist item	Location where item is reported
Certainty assessment	15	Describe any methods used to assess certainty (or confidence) in the body of evidence for an outcome.	Page 9, line 137
**RESULTS**			
Study selection	16a	Describe the results of the search and selection process, from the number of records identified in the search to the number of studies included in the review, ideally using a flow diagram.	Page 10, line 161-164, supplementary materials (S05)
	16b	Cite studies that might appear to meet the inclusion criteria, but which were excluded, and explain why they were excluded.	-
Study characteristics	17	Cite each included study and present its characteristics.	Supplementary materials (S07)
Risk of bias in studies	18	Present assessments of risk of bias for each included study.	Supplementary materials (S03)
Results of individual studies	19	For all outcomes, present, for each study: (a) summary statistics for each group (where appropriate) and (b) an effect estimate and its precision (e.g. confidence/credible interval), ideally using structured tables or plots.	Supplementary materials(S07)
Results of syntheses	20a	For each synthesis, briefly summarise the characteristics and risk of bias among contributing studies.	Page 10-11, line 166-178
	20b	Present results of all statistical syntheses conducted. If meta-analysis was done, present for each the summary estimate and its precision (e.g. confidence/credible interval) and measures of statistical heterogeneity. If comparing groups, describe the direction of the effect.	Figure 1,2 and 3, Table 1, 2 and 3
	20c	Present results of all investigations of possible causes of heterogeneity among study results.	Page 18, line 242-250; Table 3
	20d	Present results of all sensitivity analyses conducted to assess the robustness of the synthesized results.	Page 19, line 259-264; supplementary materials (S11)
Reporting biases	21	Present assessments of risk of bias due to missing results (arising from reporting biases) for each synthesis assessed.	Page 20 line 270-274; supplementary materials (S14)
Certainty of evidence	22	Present assessments of certainty (or confidence) in the body of evidence for each outcome assessed.	-
**DISCUSSION**			
Discussion	23a	Provide a general interpretation of the results in the context of other evidence.	Page 20, line 277-282
	23b	Discuss any limitations of the evidence included in the review.	Page 24-25, line 354-372
	23c	Discuss any limitations of the review processes used.	-
	23d	Discuss implications of the results for practice, policy, and future research.	Page 25, line 373-381
**OTHER INFORMATION**			
Registration and protocol	24a	Provide registration information for the review, including register name and registration number, or state that the review was not registered.	Page 8, line 112
	24b	Indicate where the review protocol can be accessed, or state that a protocol was not prepared.	-

Section and Topic	Item #	Checklist item	Location where item is reported
	24c	Describe and explain any amendments to information provided at registration or in the protocol.	-
Support	25	Describe sources of financial or non-financial support for the review, and the role of the funders or sponsors in the review.	Page 26, line 391-393
Competing interests	26	Declare any competing interests of review authors.	Page 26, line 401-403
Availability of data, code and other materials	27	Report which of the following are publicly available and where they can be found: template data collection forms; data extracted from included studies; data used for all analyses; analytic code; any other materials used in the review.	Page 27, line 404-406

From: Page MJ, McKenzie JE, Bossuyt PM, Boutron I, Hoffmann TC, Mulrow CD, et al. The PRISMA 2020 statement: an updated guideline for reporting systematic reviews. BMJ 2021;372:n71. doi: 10.1136/bmj.n71

Supplement S02: Search strategy

MEDLINE

#1 preserved ratio impaired spirometry.mp.

#2 "restrictive spirometr* pattern".ab,ti.

#3 "spirometr* restriction".ab,ti.

#4 restrictive ventilatory pattern.ab,ti.

#5 restrictive pattern.ab,ti.

#6 restrictive lung function pattern.ab,ti.

#7 restrictive pulmonary function pattern.ab,ti.

#8 restrictive lung disease.ab,ti.

#9 restrictive pulmonary disease.ab,ti.

#10 "spirometr* defect".ab,ti.

#11 restrictive defect.ab,ti.

#12 ventilatory defect.ab,ti.

#13 "spirometr* impairment".ab,ti.

#14 ventilatory impairment.ab,ti.

#15 GOLD-unclassified.ab,ti.

#16 GOLD-U.ab,ti.

#17 unclassified pattern.ab,ti.

#18 LLN-unclassified.ab,ti.

#19 LLN-U.ab,ti.

#20 "non*specific pattern".ab,ti.

#21 "abnormal spirometr*".ab,ti.

#22 "spirometr* abnormality".ab,ti.

#23 "impaired spirometr*".ab,ti.

#24 lung function impairment.ab,ti.

#25 1 or 2 or 3 or 4 or 5 or 6 or 7 or 8 or 9 or 10 or 11 or 12 or 13 or 14 or 15 or 16 or 17 or 18 or 19 or 20 or 21 or 22 or 23 or 24

#26 *morbidity/ or exp prevalence/ or *mortality/

#27 *risk factors/

#28 (burden adj3 disease).mp. [mp=title, book title, abstract, original title, name of substance word, subject heading word, floating sub-heading word, keyword heading word, organism supplementary concept word, protocol supplementary concept word, rare disease supplementary concept word, unique identifier, synonyms, population supplementary concept word, anatomy supplementary concept word]

#29 predictive factors.ab,ti.

#30 26 or 27 or 28 or 29

#31 25 and 30

Overall hits: 268

Embase

#1 preserved ratio impaired spirometry.mp.

#2 "restrictive spirometr* pattern".ab,ti.

#3 "spirometr* restriction".ab,ti.

#4 restrictive ventilatory pattern.ab,ti.

#5 restrictive pattern.ab,ti.

#6 restrictive lung function pattern.ab,ti.

#7 restrictive pulmonary function pattern.ab,ti.

#8 restrictive lung disease.ab,ti.

#9 restrictive pulmonary disease.ab,ti.

#10 "spirometr* defect".ab,ti.

#11 restrictive defect.ab,ti.

#12 ventilatory defect.ab,ti.

#13 "spirometr* impairment".ab,ti.

#14 ventilatory impairment.ab,ti.

#15 GOLD-unclassified.ab,ti.

#16 GOLD-U.ab,ti.

#17 unclassified pattern.ab,ti.

#18 LLN-unclassified.ab,ti.

#19 LLN-U.ab,ti.

#20 "non*specific pattern".ab,ti.

#21 "abnormal spirometr*".ab,ti.

#22 "spirometr* abnormality".ab,ti.

#23 "impaired spirometr*".ab,ti.

#24 lung function impairment.ab,ti.

#25 1 or 2 or 3 or 4 or 5 or 6 or 7 or 8 or 9 or 10 or 11 or 12 or 13 or 14 or 15 or 16 or 17 or 18 or 19 or 20 or 21 or 22 or 23 or 24

#26 *morbidity/ or exp prevalence/ or *mortality/

#27 *risk factors/

#28 (burden adj3 disease).mp. [mp=title, book title, abstract, original title, name of substance word, subject heading word, floating sub-heading word, keyword heading word, organism supplementary concept word, protocol supplementary concept word, rare disease supplementary concept word, unique identifier, synonyms, population supplementary concept word, anatomy supplementary concept word]

#29 predictive factors.ab,ti.

#30 26 or 27 or 28 or 29

#31 25 and 30

Overall hits: 1165

WANFANG Database

#1 保留比值受损肺功能 or 保留比率的肺功能减损 or 肺功能保留 or 保留比例肺功能受损 or 保存率肺功能受损

#2 preserved ratio impaired spirometry

#3 未分类肺功能

#4 非特异性肺通气 or 非特异性肺功能 or 肺功能非特异性

#5 #1 or #2 or #3 or #4

#6 限制性通气功能障碍 or 限制型通气功能障碍 or 限制型肺通气功能障碍 or 限制性肺通气功能障碍 or 限制性肺疾病

#7 restrictive ventilatory pattern

#8 #6 or #7

#9 #5 or #8

语种 中文

Overall hits: 1694

Supplement S03: Quality assessment

The Joanna Briggs Institute (JBI) Critical Appraisal Tools for prevalence studies for used for quality assessment of our included studies. [62] Assessments were completed by two reviewers for each study, marking each criterion as 'yes', 'no', or 'unclear'. Conflicts were resolved in a discussion. The number of 'yes' responses for each study (total) were added, and studies with score ≥7 were considered as 'high-quality', and included in our sensitivity analysis.

Quality assessment for prevalence studies

JBI criteria:

Q1: Was the sample frame appropriate to address the target population?
Q2: Were study participants sampled in an appropriate way?
Q3: Was the sample size adequate?
Q4: Were the study subjects and the setting described in detail?
Q5: Was the data analysis conducted with sufficient coverage of the identified sample?
Q6: Were valid methods used for the identification of the condition?
Q7: Was the condition measured in a standard, reliable way for all participants?
Q8: Was there appropriate statistical analysis?
Q9: Was the response rate adequate, and if not, was the low response rate managed appropriately?

Study	Q1	Q2	Q3	Q4	Q5	Q6	Q7	Q8	Q9	Total
Backman et. al. (2016) (1)	Yes	Yes	Yes	Yes	Yes	Yes	Yes	Yes	Yes	9
Cadham et. al. (2024) (2)	Yes	Yes	Yes	Yes	No	Yes	Yes	Yes	Yes	8
Carsin et. al. (2019) (3)	Yes	Yes	Yes	Yes	No	Yes	Yes	Yes	No	7
Cestelli et. al. (2025) (4)	No	Yes	Yes	Yes	Unclear	Yes	Yes	Yes	Yes	7
Choi et. al. (2024) (5)	Yes	Yes	Yes	Yes	Yes	Yes	Yes	Yes	Yes	9
Domingo-Relloso et. al. (2022) (6)	Yes	Unclear	Yes	Yes	Unclear	Yes	Yes	Yes	Unclear	6
Eriksson et. al. (2013) (7)	Yes	Yes	Yes	Yes	Yes	Yes	Yes	Yes	Yes	9
Fimognari et. al. (2007) (8)	Yes	No	Yes	Yes	Unclear	Yes	Yes	Yes	Unclear	6
Ford et. al. (2014) (9)	Yes	Yes	Yes	Yes	Yes	Yes	Yes	Yes	Yes	9

Fragoso et. al. (2011) (10)	Yes	Yes	Yes	Yes	Yes	Yes	Yes	Yes	Yes	9
Guerra et. al. (2017) (11)	Yes	Yes	Yes	Yes	Yes	Yes	Yes	Yes	Yes	9
Guerra et. al. (2010) (12)	Yes	Yes	Yes	Yes	Unclear	Yes	Yes	Yes	Yes	8
Higbee et. al. (2022) (13)	Yes	Yes	Yes	Yes	Yes	Yes	Yes	Yes	Yes	9
Joshi et. al. (2021) (14)	Yes	Yes	Yes	Yes	Yes	Yes	Yes	Yes	Yes	9
Kaaks et. al. (2022) (15)	Yes	Unclear	Yes	Yes	Yes	Yes	Yes	Yes	Yes	8
Kaise et. al. (2021) (16)	Yes	No	Yes	Yes	Unclear	Yes	Yes	Yes	Unclear	6
Kanetake et. al. (2022) (17)	Yes	Unclear	Yes	Yes	Unclear	Yes	Yes	Yes	Unclear	6
Kang et. al. (2024) (18)	Yes	Yes	Yes	Yes	Yes	Yes	Yes	Yes	Yes	9
Kim et. al. (2022) (19)	Yes	Yes	Yes	Yes	Unclear	Yes	Yes	Yes	Yes	8
Kwon et. Al. (2024) (20)	Yes	Yes	Yes	Yes	No	Yes	Yes	Yes	Unclear	7
Lee et. al. (2020) (21)	Yes	Unclear	Yes	Yes	Unclear	Yes	Yes	Yes	Unclear	6
Li et. al. (2025) (22)	Yes	Yes	Yes	Yes	Yes	Yes	Yes	Yes	Yes	9
Lim et. al. (2023) (23)	Yes	No	Yes	Yes	Yes	Yes	Yes	Yes	Yes	8
Lin et. al. (2021) (24)	Yes	Unclear	Yes	Yes	Unclear	Yes	Yes	Yes	Unclear	6
Mannino et. al. (2003a) (25)	Yes	Yes	Yes	Yes	Yes	Yes	Yes	Yes	Yes	9
Mannino et. al. (2003b) (26)	Yes	Yes	Yes	Yes	Unclear	Yes	Yes	Yes	Unclear	7
Mannino and Diaz-Guzman (2012) (27)	Yes	Yes	Yes	Yes	Unclear	Yes	Yes	Yes	Yes	8
Mannino et. al. (2012) (28)	Yes	Yes	Yes	Yes	Unclear	Yes	Yes	Yes	Unclear	7
Meghji et. al. (2016) (29)	Yes	Yes	Yes	Yes	Yes	Yes	Yes	Yes	Yes	9
Nonato et. al. (2015) (30)	Yes	Yes	Yes	Yes	Yes	Yes	Yes	Yes	Yes	9
Paek et. al. (2010) (31)	Yes	Yes	Yes	Yes	No	Yes	Yes	Yes	Unclear	7
Pefura-Yone et. al. (2016) (32)	Yes	Yes	Yes	Yes	Yes	Yes	Yes	Yes	Yes	9
Perez-Padilla et. al. (2023) (33)	Yes	Yes	Yes	Yes	Yes	Yes	Yes	Yes	Yes	9
Qiao et. al. (2018) (34)	Yes	Yes	Yes	Yes	Yes	Unclear	Yes	Yes	Yes	8
Schwartz et. al. (2021) (35)	Yes	Yes	Yes	Yes	Yes	Yes	Yes	Yes	Yes	9
Shiraishi et. al. (2022) (36)	Yes	Unclear	Yes	Yes	No	Yes	Yes	Yes	Unclear	6
Shu et. al. (2024) (37)	Yes	Yes	Yes	Yes	Unclear	Yes	Yes	Yes	Yes	8
Siddharthan et. al. (2017) (38)	Yes	Yes	Yes	Yes	Yes	Yes	Yes	Yes	Yes	9
Siddharthan et. al. (2019) (39)	Yes	Yes	Yes	Yes	Yes	Yes	Yes	Yes	Yes	9

Siddharthan et. al. (2024) (40)	Yes	Yes	Yes	Yes	Yes	Yes	Yes	Yes	Yes	9
Sin et. al. (2023) (41)	Yes	Unclear	Yes	Yes	Yes	Yes	Yes	Yes	Unclear	7
Soriano et. al. (2012) (42)	Yes	Yes	Yes	Yes	Yes	Yes	Yes	Yes	Yes	9
Sperandio et. al. (2015) (43)	Yes	No	Yes	Yes	Unclear	Yes	Yes	Yes	Unclear	6
Tanabe. et al. (2022) (44)	Unclear	Unclear	Yes	Yes	Unclear	Yes	Yes	Yes	Unclear	5
Toren et. al. (2024) (45)	Yes	Yes	Yes	Yes	Yes	Yes	Yes	Yes	Yes	9
Tran et. al. (2023) (46)	Yes	No	Yes	Yes	No	Yes	Yes	Yes	No	6
Wan et. al. (2014) (47)	Yes	Unclear	Yes	Yes	Unclear	Yes	Yes	Yes	Unclear	6
Wan et. al. (2021) (48)	Yes	No	Yes	Yes	Unclear	Yes	Yes	Yes	Unclear	6
Wang et. al. (2022) (49)	Yes	Yes	Yes	Yes	Unclear	Yes	Yes	Yes	Yes	8
Wang et. al. (2024) (50)	Yes	Yes	Yes	Yes	Unclear	Yes	Yes	Yes	Unclear	7
Wijnant et. al. (2020) (51)	Yes	Yes	Yes	Yes	Unclear	Yes	Yes	Yes	Yes	8
Xia et. al. (2025) (52)	Yes	Yes	Yes	Yes	No	Yes	Yes	Yes	Yes	8
Xiao et. al. (2025) (53)	Yes	Yes	Yes	Yes	No	Yes	Yes	Yes	Unclear	7
Zhang et. al. (2024) (54)	Yes	Yes	Yes	Yes	Unclear	Yes	Yes	Yes	Unclear	7
Zhou et. al. (2022) (55)	Yes	Yes	Yes	Yes	Unclear	Yes	Yes	Yes	Yes	8
Zhou et. al. (2025) (56)	Yes	Yes	Yes	Yes	Unclear	Yes	Yes	Yes	Yes	8
Zielinski et. al. (2005) (57)	Yes	Yes	Yes	Yes	Yes	Yes	Yes	Yes	Yes	9

Supplement S04: Mixed-effect meta-regression – Multivariable models adjusted for all moderators (GOLD analysis)

Table 4.1: Results of multivariable mixed-effect meta-regression – models adjusted with all *a-priori* selected moderators

Variable	Group	GOLD-PRISm			GOLD-RSP		
		β (95% CI) (logit proportion)	p	R^2 (%)	β (95% CI) (logit proportion)	p	R^2 (%)
**Multivariable meta-regression (full models)**							
WHO geographical location (ref: WPRO)	AFRO	-	-	4.49	0.65 (-2.06 to 3.36)	0.64	63.19
	AMRO	-0.01 (-1.04 to 1.03)	0.99		-2.08 (-4.35 to 0.18)	0.07	
	EURO	-0.44 (-1.68 to 0.8)	0.48		-2.14 (-4.05 to -0.23)	0.03	
	Multi	1.06 (-1.13 to 3.26)	0.34		-	-	
World Bank income level (ref: HIC)	LIC	-	-		-*	-*	
	UMIC	0.81 (-0.17 to 1.8)	0.10		-*	-*	
	Mixed	-1.18 (-3.32 to 0.96)	0.28		-	-	
Bronchodilator use (ref: No)		0.12 (-0.98 to 1.21)	0.84		-0.67 (-1.75 to 0.42)	0.23	
Mean age		-0.02 (-0.08 to 0.05)	0.65		0.03 (-0.01 to 0.06)	0.18	
% females in study population		0.03 (-0.07 to 0.12)	0.60		0.02 (-0.04 to 0.09)	0.46	
% current smokers in study population		0.02 (-0.05 to 0.08)	0.61		0.00 (-0.01 to 0.02)	0.52	
Publication year		-0.02 (-0.18 to 0.14)	0.79		0.01 (-0.03 to 0.05)	0.76	

Note: AFRO – WHO African Region, AMRO – WHO Region of the Americas, CI – confidence interval, EURO – WHO European Region, GOLD – Global Initiative for Obstructive Lung Disease, HIC – high-income country, LIC – low-income country, MLMA – multi-level meta-analysis, PI – prediction interval, PRISm – preserved ratio impaired spirometry, RSP – restrictive spirometric pattern, and UMIC – upper-middle income country, WHO – World Health Organization and WPRO – WHO Western Pacific Region.

*World Bank income levels were automatically dropped from GOLD-RSP multivariable model due to being redundant

Supplement S05: PRISMA flowchart showing study inclusion process

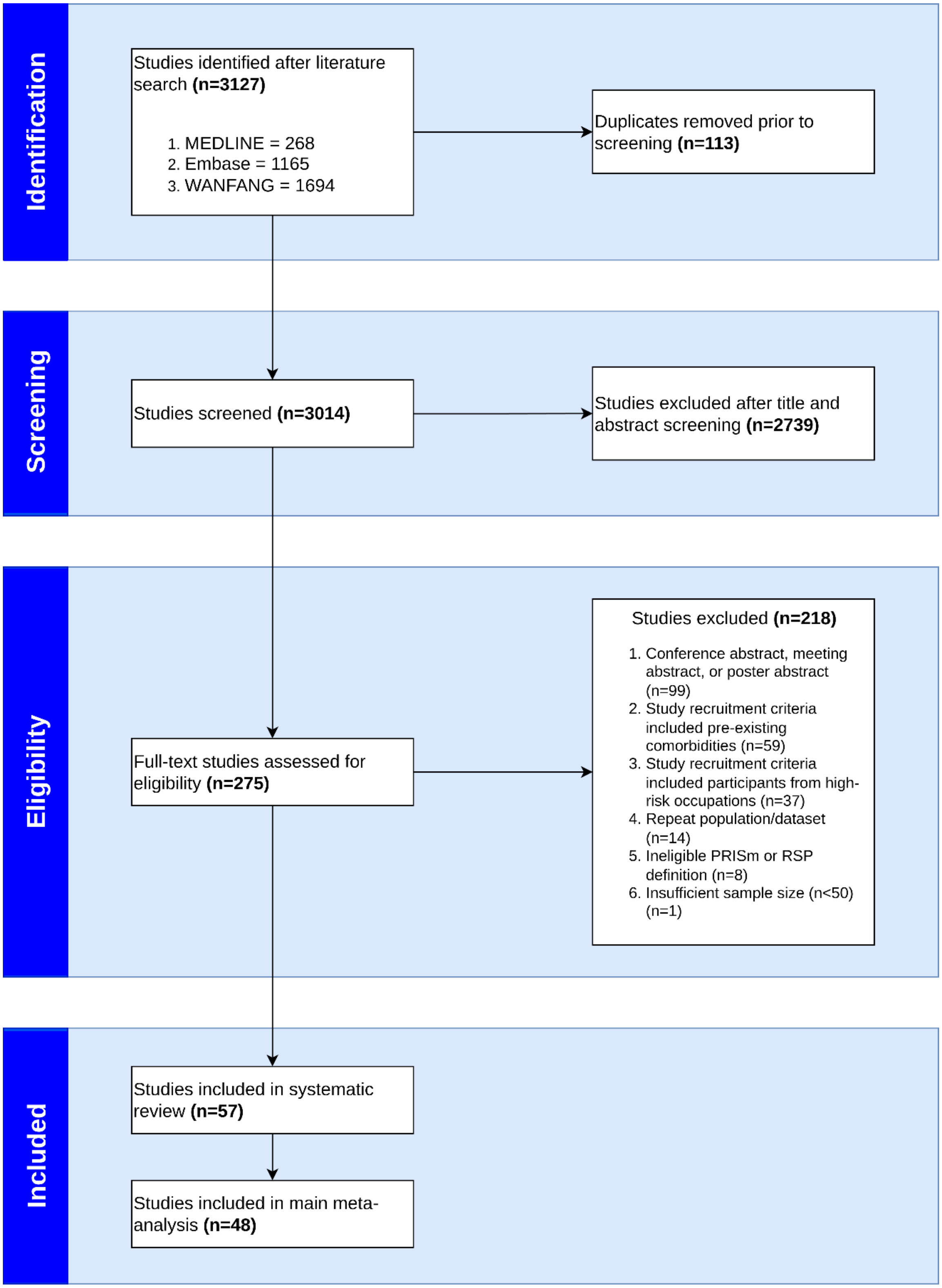

Supplement S06: Map showing global distribution of included studies

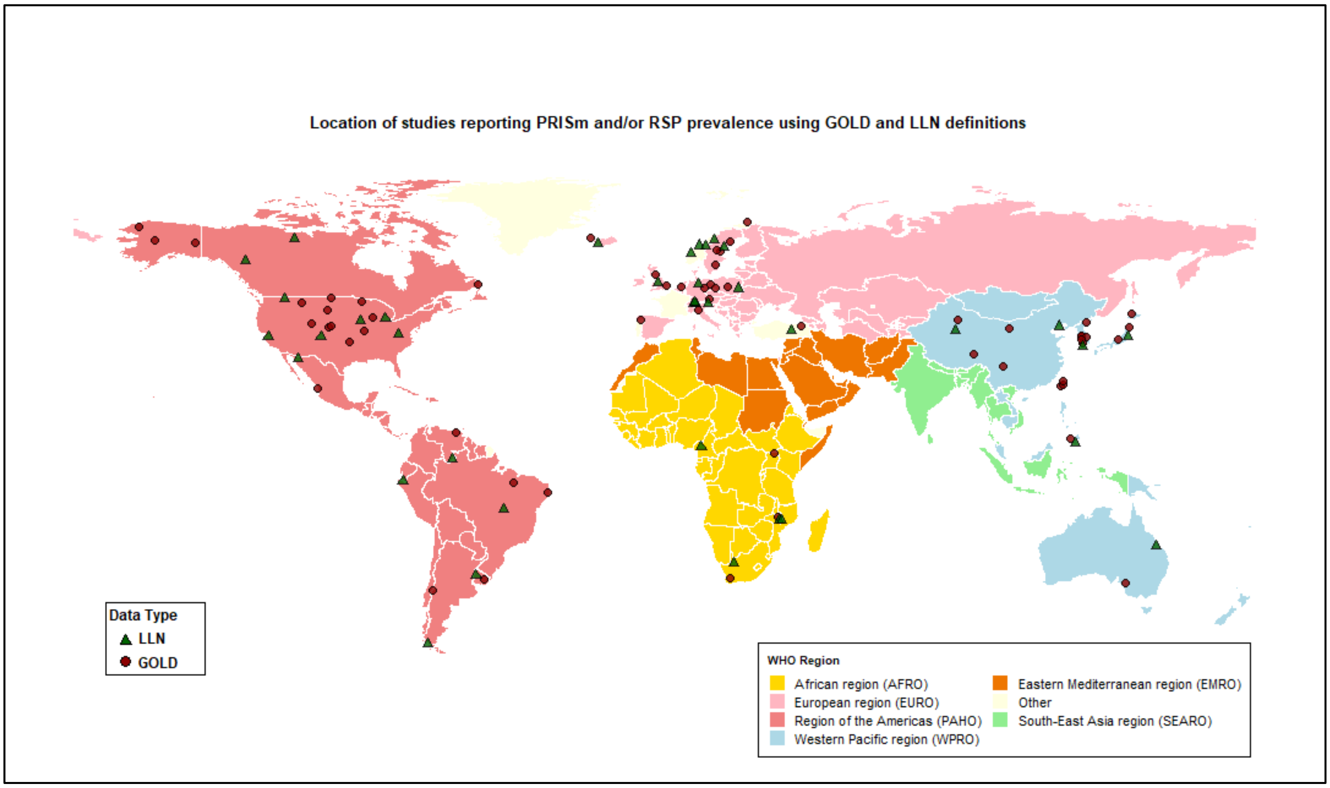

Supplement S07: Baseline characteristics of included studies (k=57)

Table 7.1: Baseline characteristics of reported studies

Sl.	Study	Location (WHO region)	World Bank income level	Diagnosis definition	Use of bronchodilator before spirometry	Outcome reported	Total sample size (n)	Cases (n)	Prevalence reported (%)	QA score[a]	Meta-analysis inclusion
01.	Backman et. al. (2016) (1)	Sweden (EUR)	High	GOLD[b]	No	RSP	726	76	10.47	9	Main and supplementary analysis
				LLN				68	9.37		
				GOLD	Yes			53	7.30		Excluded from meta-analysis
				LLN				44	6.06		
02.	Cadham et. al. (2024) (2)	USA (AMR)	High	GOLD	No	PRISm	13328	1862	13.97	8	Main analysis
03.	Carsin et. al. (2019) (3)	Switzerland and Europe (EUR)	High	LLN	Yes	RSP	8291	423	5.10	7	Supplementary analysis
04.	Cestelli et. al. (2025) (4)	Norway (EUR)	High	GOLD	No	PRISm	26091	1110	4.25	7	Main and supplementary analysis – only included in prevalence of males as this study only recruited males. Not included in overall prevalence calculations.
				GOLD		RSP		956	3.66		
				LLN		PRISm		1270	4.87		
				LLN		RSP		1348	5.17		
05.	Choi et. al. (2024) (5)	South Korea (WPR)	High	GOLD	No	PRISm	32949	3803	11.54	9	Main analysis
06.	Domingo-Relloso et. al. (2022) (6)	United States (AMR)	High	GOLD	No	RSP	1677	229	13.66	6	Main analysis

07.	Eriksson et. al. (2013) (7)	Sweden (EUR)	High	GOLD	No	RSP	642	18	2.8	9	Main analysis
08.	Fimognari et. al. (2007) (8)	Italy (EUR)	High	GOLD	Not specified	RSP	159	25	15.72	6	Main analysis
09.	Ford et. al. (2014) (09)	United States (AMR)	High	GOLD	No	RSP	3109	187	6.00	9	Main analysis
10.	Fragoso et. al. (2011) (10)	United States (AMR)	High	GOLD	No	RSP	5080	563	11.08	9	Main analysis
				LLN				475	9.35		Supplementary analysis
11.	Guerra et. al. (2017) (11)	Switzerland and Europe (EUR)	High	LLN	No	RSP	12767	554	4.34	9	Supplementary analysis
12.	Guerra et. al. (2010) (12)	United States (AMR)	High	GOLD	No	RSP	2048	249	12.16	8	Main analysis
13.	Higbee et. al. (2022) (13)	United Kingdom (EUR)	High	GOLD	No	PRISm	351874	38639	10.98	9	Main analysis – only included in prevalence of genders due to data duplication with Kang et. al. (2024).
14.	Joshi et. al. (2021) (14)	Canada (AMR)	High	LLN	No	RSP	44816	3496	7.80	9	Supplementary analysis
15.	Kaaks et. al. (2022) (15)	Germany (EUR)	High	GOLD	No	PRISm	1987	311	15.70	8	Main analysis – only included in smoker prevalence as the study only recruited smokers. Not included in overall prevalence calculations.

16.	Kaise et. al. (2021) (16)	Japan (WPR)	High	GOLD	No	PRISm	2518	420	16.68	6	Main analysis
17.	Kanetake et. al. (2022) (17)	Japan (WPR)	High	LLN	No	PRISm	1672	176	10.53	6	Supplementary analysis
18.	Kang et. al. (2024) (18)	UK (EUR)	High	GOLD	No	PRISm	420002	55365	13.18	9	Main analysis – overall, smokers and combined meta-analysis. Not included in sex prevalence calculation due to missing data.
19.	Kim et. al. (2022) (19)	South Korea (WPR)	High	GOLD	No	PRISm	17515	1563	8.92	8	Main analysis
20.	Kwon et. Al. (2024) (20)	South Korea (WPR)	High	GOLD	No	RSP	22387	2535	11.32	7	Main analysis – only sex and smoking habit prevalence data is included from this study as this study has the same dataset as Choi et. al. 2024 and that study did not provide data on sex and smoking habits.
21.	Lee et. al. (2020) (21)	Taiwan (WPR)	High	GOLD	Not specified	RSP	6945	1495	21.53	6	Main analysis
22.	Li et. al. (2025) (22)	China (WPR)	Upper-middle	GOLD	No	PRISm	40279	11824	29.36	9	Main analysis
23.	Lim et. al. (2023) (23)	South Korea (WPR)	High	GOLD	No	RSP	26343	4725	17.93	8	Main analysis
24.	Lin et. al. (2021) (24)	Taiwan (WPR)	High	GOLD	Not specified	RSP	1563	524	31.7	6	Main analysis

25.	Mannino et. al. (2003a) (25)	USA (AMR)	High	GOLD	No	RSP	5542	510	9.2	9	Main analysis
26.	Mannino et. al. (2003b) (26)	USA (AMR)	High	GOLD	No	RSP	7503	495	6.6	7	Main analysis
27.	Mannino and Diaz-Guzman (2012) (27)	USA (AMR)	High	GOLD	No	RSP	13847	1445	10.44	8	Main analysis
				LLN				1180	8.52		Supplementary analysis
28.	Mannino et. al. (2012) (28)	Multiple locations - China, Turkey, Austria, South Africa, Iceland, Germany, Poland, Norway, Canada, USA, The Philippines, Australia, UK, Sweden	High	GOLD	Yes	RSP	9762	1382	14.16	7	Main analysis
29.	Meghji et. al. (2016) (29)	Malawi (AFR)	Low	GOLD	Yes	RSP	1057	408	38.6	9	Main analysis
				LLN				95	9.00		Supplementary analysis
30.	Nonato et. al. (2015) (30)	Multiple locations - Latin America - Brazil (SA), Chile (SA), Uruguay (SA), Venezuela	-	LLN	Yes	RSP	5315	260	4.89	9	Supplementary analysis

		(SA), Mexico (NA) (AMR)									
31.	Paek et. al. (2010) (31)	South Korea (WPR)	High	GOLD	No	RSP	4001	217	5.42	7	Main analysis
32.	Pefura-Yone et. al. (2016) (32)	Cameroon (AFR)	Lower-middle	LLN	Not specfied	RSP	1003	189	18.80	9	Supplementary analysis
33.	Perez-Padilla et. al. (2023) (33)	Multiple locations - Latin America - Brazil (SA), Chile (SA), Uruguay (SA), Venezuela (SA), Mexico (NA) (AMR)	-	GOLD	Yes	PRISm	2942	146	4.96	9	Main analysis
34.	Qiao et. al. (2018) (34)	China (WPR)	Upper-middle	LLN	Yes	RSP	1082	104	9.61	8	Main analysis
35.	Schwartz et. al. (2021) (35)	USA (AMR)	High	LLN	No	PRISm	8494	4030	22.32	9	Supplementary analysis
					Yes			1507	17.74		Excluded from analysis
36.	Shiraishi et. al. (2022) (36)	Japan (WPR)	High	GOLD	No	PRISm	1818	173	9.52	6	Main analysis
37.	Shu et. al. (2024) (37)	Taiwan (WPR)	High	GOLD	No	PRISm	461183	65832	14.27	8	Main analysis

38.	Siddharthan et. al. (2017) (38)	Peru (AMR)	Upper-middle	LLN	Yes	RSP	3055	144	4.70	9	Supplementary analysis
39.	Siddharthan et. al. (2019) (39)	Uganda (AFR)	Low	GOLD	Yes	RSP	1502	154	10.24	9	Main analysis
40.	Siddharthan et. al. (2024) (40)	Multiple locations – Nepal, Peru, Uganda	-	GOLD	Yes	PRISm	10664	986	9.25	9	Main analysis
41.	Sin et. al. (2023) (41)	South Korea (WPR)	High	GOLD	No	PRISm	7526	471	6.26	7	Main analysis
				LLN				487	6.47		Supplementary analysis
42.	Soriano et. al. (2012) (42)	Spain (EUR)	High	GOLD	No	RSP	3802	481	12.65	9	Main analysis
43.	Sperandio et. al. (2015) (43)	Brasil (AMR)	Upper-middle	GOLD	Yes	RSP	374	37	9.89	6	Main analysis
44.	Tanabe. et al. (2022) (44)	Japan (WPR)	High	GOLD	No	PRISm	10139	706	6.96	5	Main analysis
45.	Toren et. al. (2024) (45)	Sweden (EUR)	High	GOLD	No	PRISm	28855	1467	5.08	9	Main analysis – only PRISm data included for combined prevalence calculations.
				GOLD		RSP		1475	5.11		
				LLN		PRISm		562	1.95		Supplementary analysis
				LLN		RSP		588	2.04		

46.	Tran et. al. (2023) (46)	USA (AMR)	High	GOLD	Yes	PRISm	5055	685	13.55	6	Main analysis – only included in smoker prevalence as the study only recruited smokers. Not included in overall prevalence calculations.
47.	Wan et. al. (2014) (47)	USA (AMR)	High	GOLD	Yes	PRISm	10192	1257	12.33	6	Main and supplementary analysis – only included in smoker prevalence as the study only recruited smokers. Not included in overall prevalence calculations.
				LLN			9492	1082	11.40		
48.	Wan et. al. (2021) (48)	USA (AMR)	High	GOLD	No	PRISm	53701	4582	8.53	6	Main analysis
49.	Wang et. al. (2022) (49)	China (WPR)	Upper-middle	LLN	No	RSP	61624	4983	8.09	8	Supplementary analysis
50.	Wang et. al. (2024) (50)	USA (AMR)	High	GOLD	Not specified	RSP	4338	320	7.38	7	Main analysis
51.	Wijnant et. al. (2020) (51)	Netherlands (EUR)	High	GOLD	No	PRISm	5487	387	7.05	8	Main analysis
52.	Xia et. al. (2025) (52)	China (WPR)	Upper-middle	GOLD	No	PRISm	627	63	10.05	8	Main analysis
53.	Xiao et. al. (2025) (53)	USA (AMR)	High	GOLD	No	PRISm	24691	1452	5.88	7	Main analysis – only risk factor data was included from this study. It was removed from prevalence calculations due to data duplication with Ford et. al. 2014 and Mannino and Diaz-Guzman 2012.

54.	Zhang et. al. (2024) (54)	China (WPR)	Upper-middle	GOLD	No	PRISm	6994	1997	28.55	7	Main analysis
55.	Zhou et. al. (2022) (55)	China (WPR)	Upper-middle	GOLD	Not specified	RSP	4955	1160	23.41	8	Main analysis
56.	Zhou et. al. (2025) (56)	Australia (WPR)	High	GOLD	Yes	PRISm	3518	387	11.00	8	Main analysis
57.	Zielinski et. al. (2005) (57)	Poland (EUR)	High	GOLD	Not specified	RSP	91859	7624	8.30	9	Main analysis

Note: AFRO – WHO African Region, AMRO – WHO Region of the Americas, EURO – WHO European Region, GOLD – Global Initiative for Obstructive Lung Disease, LLN – lower limit of normal, PRISm – preserved ratio impaired spirometry, RSP – restrictive spirometric pattern, WHO – World Health Organization, WPRO – WHO Western Pacific Region

[a] Quality assessment was performed using the Joanna Briggs Institute (JBI) checklist for prevalence studies. The maximum possible score was 9 and assessment was carried out independently by two reviewers. Conflicts were resolved by a third reviewer.

[b] GOLD definitions: PRISm – FEV1/FVC > 0.70 and FEV1 < 80% predicted, RSP – FEV1/FVC > 0.70 and FVC < 80%;

LLN definitions: PRISm – FEV1/FVC > LLN and FEV1 < LLN, RSP – FEV1/FVC > LLN and FVC < LLN

Supplement S08: Consolidated overall prevalences from multi-level meta-analysis and random-effects models (main analysis)

Table 8.1: Consoldated overall prevalences from different modelling approaches

Modelling approach	GOLD-PRISm			GOLD-RSP			Combined GOLD-PRISm + GOLD-RSP		
	I^2 (%)	Prevalence (%, 95% CI)	95% PI	I^2 (%)	Prevalence (%, 95% CI)	95% PI	I^2 (%)	Prevalence (%, 95% CI)	95% PI
MLMA – WHO geographical regions (reported in main manuscript)	**Level 2:** 95.1<br>**Level 3:** 4.8<br>**Total:** 99.9	10.60 (8.12-13.73)	3.56-27.57	**Level 2:** 71.4<br>**Level 3:** 28.4<br>**Total:** 99.7	12.09 (7.90-18.04)	2.96-38.22	**Level 2:** 88.0<br>**Level 3:** 11.9<br>**Total:** 99.9	11.79 (9.11-15.12)	3.64-32.09
MLMA – World Bank income-levels	**Level 2:** 48.7<br>**Level 3:** 51.2<br>**Total:** 99.9	12.14 (6.97-20.30)	3.08-37.50	**Level 2:** 86.3<br>**Level 3:** 13.4<br>**Total:** 99.7	12.51 (8.28-18.46)	3.12-38.82	**Level 2:** 79.8<br>**Level 3:** 20.1<br>**Total:** 99.9	13.67 (9.69-18.93)	4.04-37.34
Random-effects model	99.9	10.87 (8.61-13.63)	3.44-29.47	99.6	11.26 (8.71-14.45)	2.84-35.55	99.8	11.85 (9.94-14.07)	3.66-32.25

Note: CI – confidence interval, GOLD – Global Initiative for Obstructive Lung Disease, MLMA – multi-level meta-analysis, PI – prediction interval, PRISm – preserved ratio impaired spirometry, RSP – restrictive spirometric pattern, and WHO – World Health Organization.

Supplement S09: Forest plots – GOLD studies (main analysis)

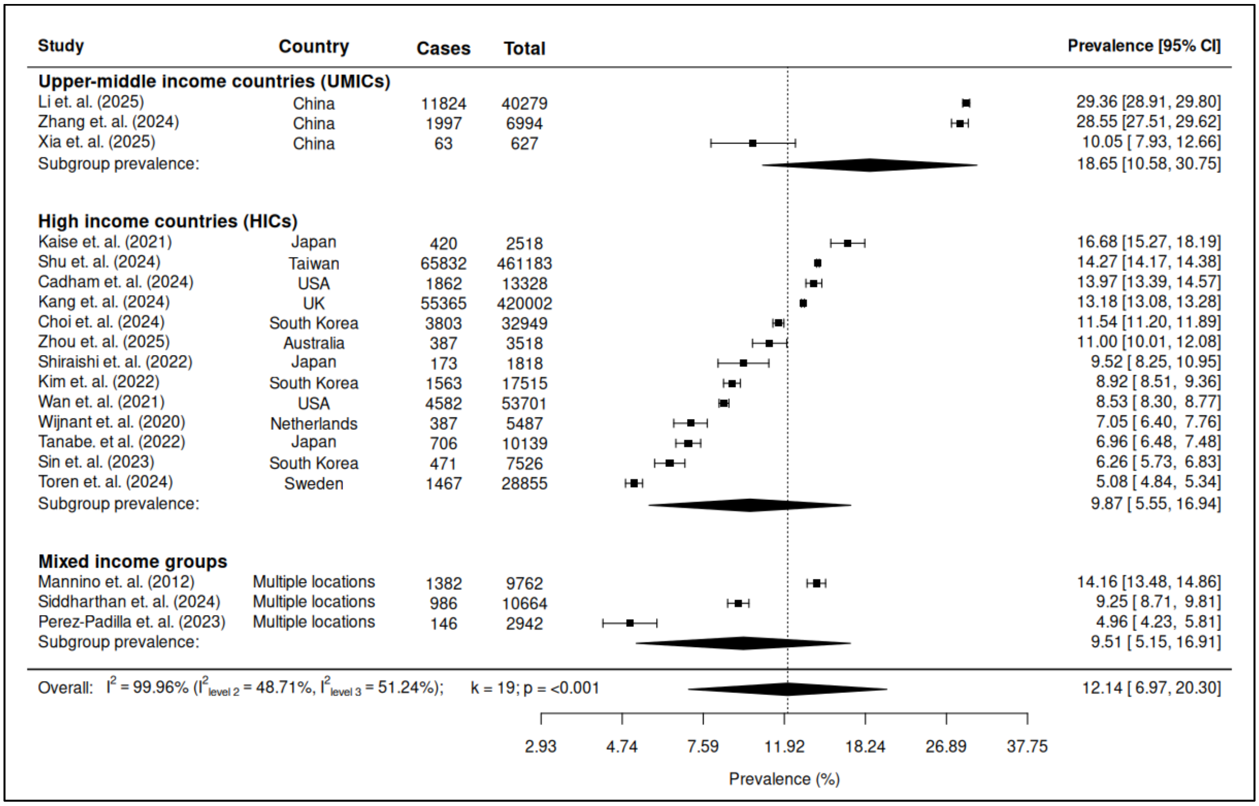

Figure 9.1: Prevalence of GOLD-PRISm (MLMA – World Bank income-level model)

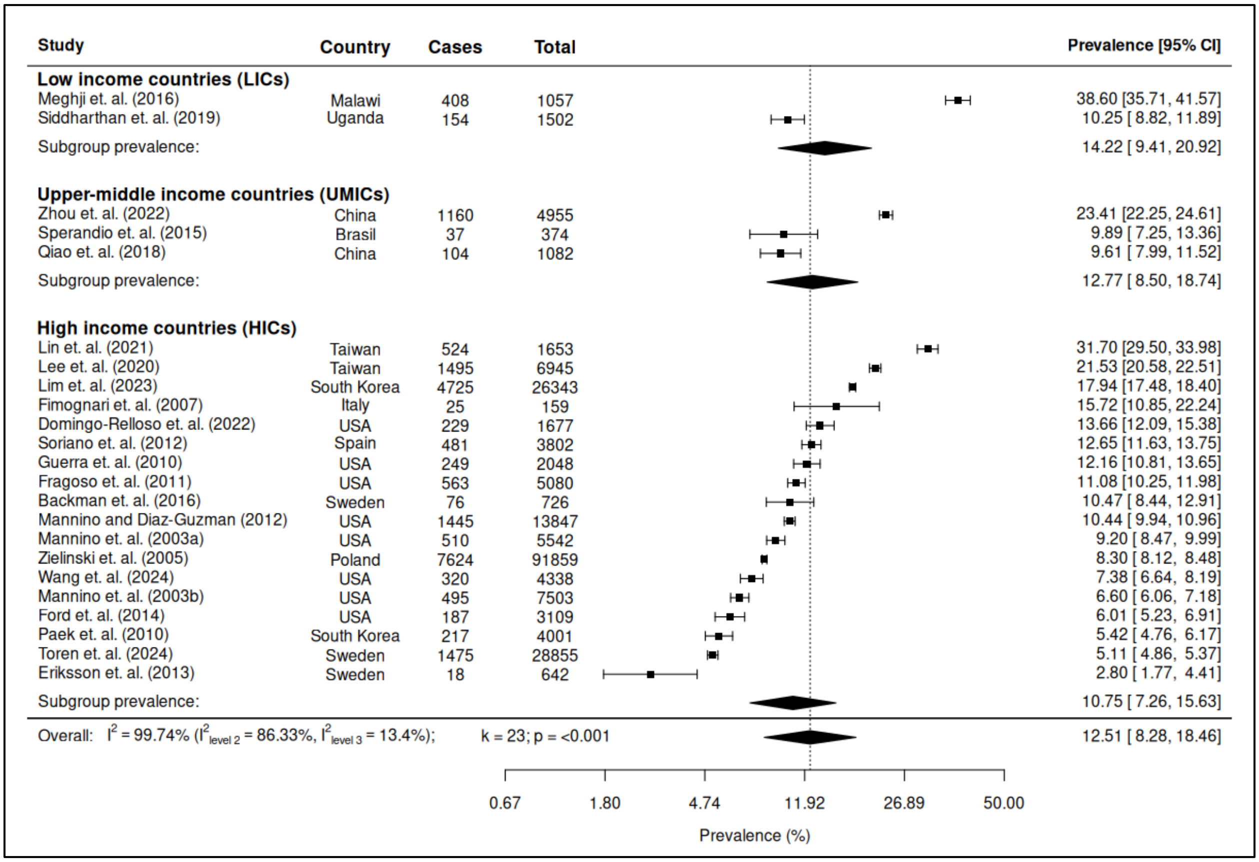

Figure 9.2: Prevalence of GOLD-RSP (MLMA – World Bank income-level model)

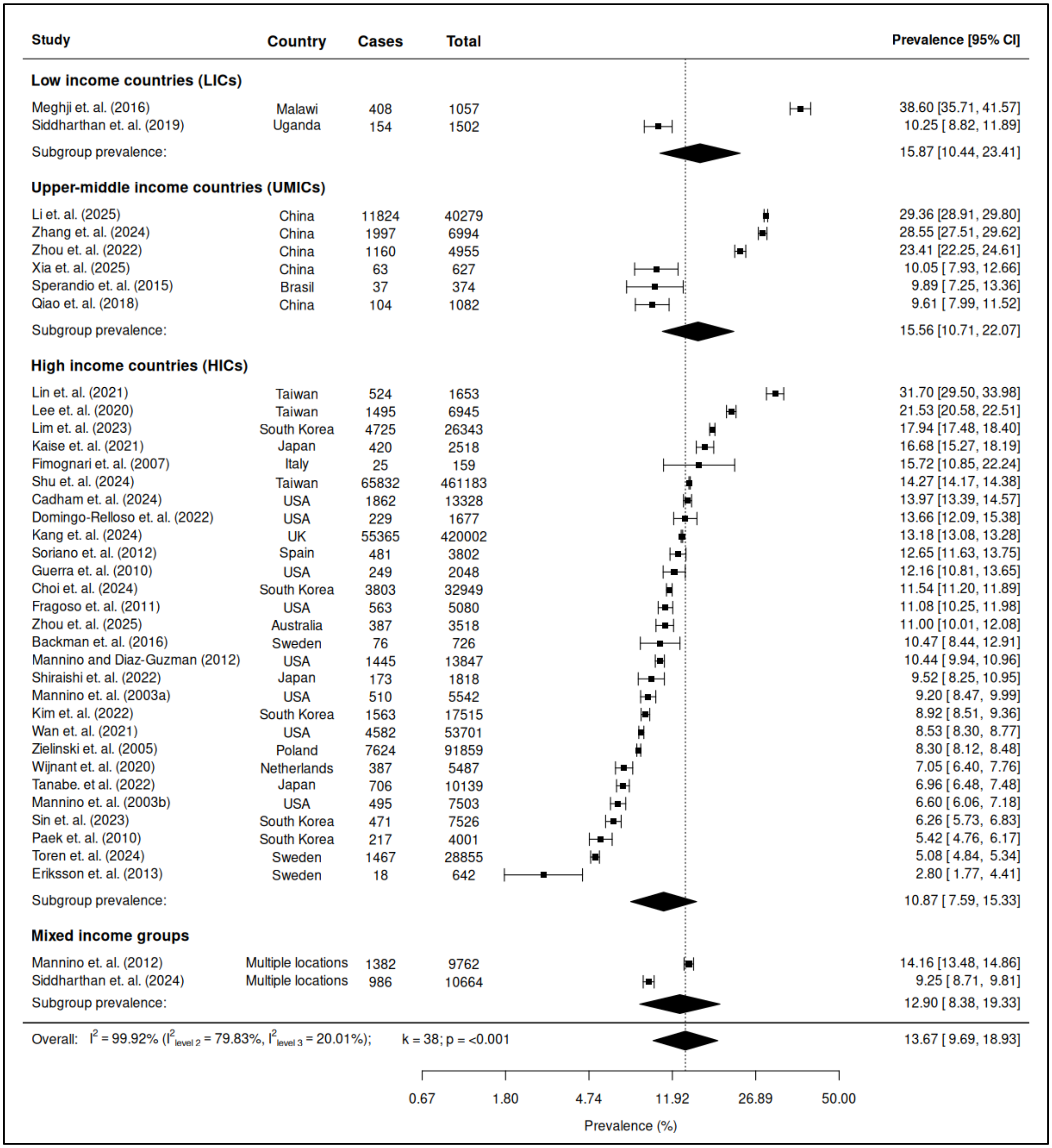

Figure 9.3: Prevalence of combined GOLD-PRISm and GOLD-RSP (MLMA – World Bank income-level model)

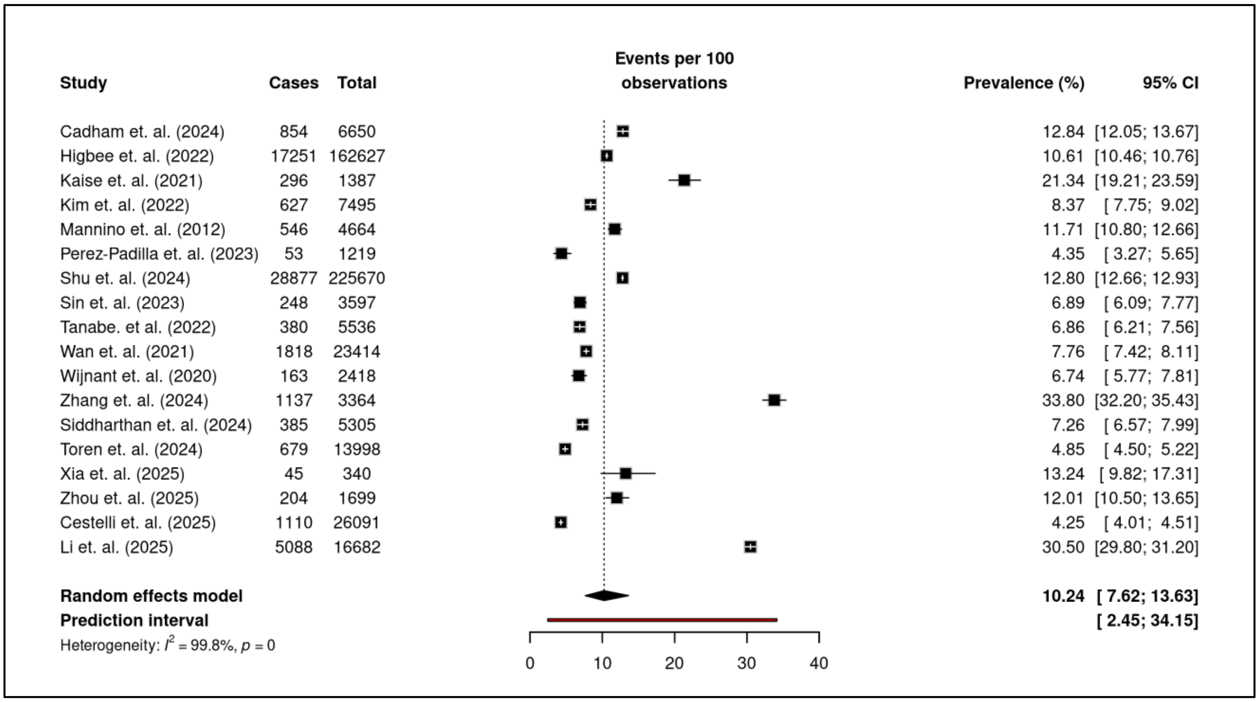

Figure 9.4: Prevalence of GOLD-PRISm in males

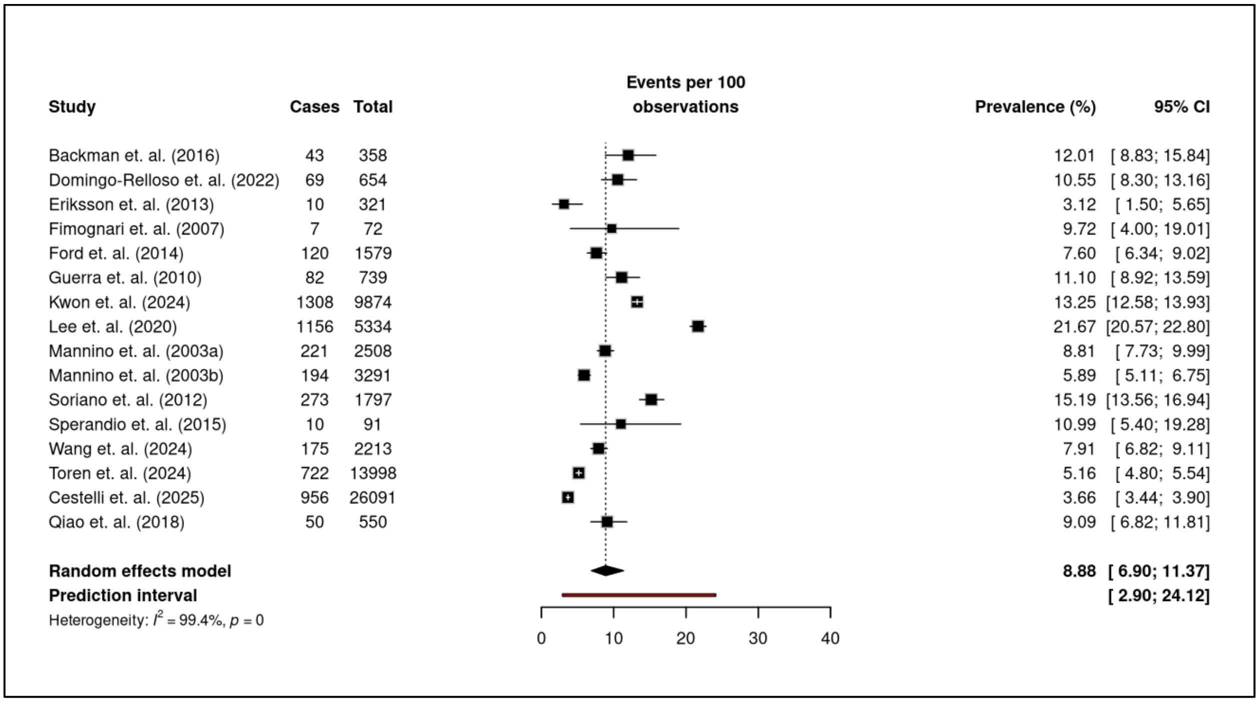

Figure 9.5: Prevalence of GOLD-RSP in males

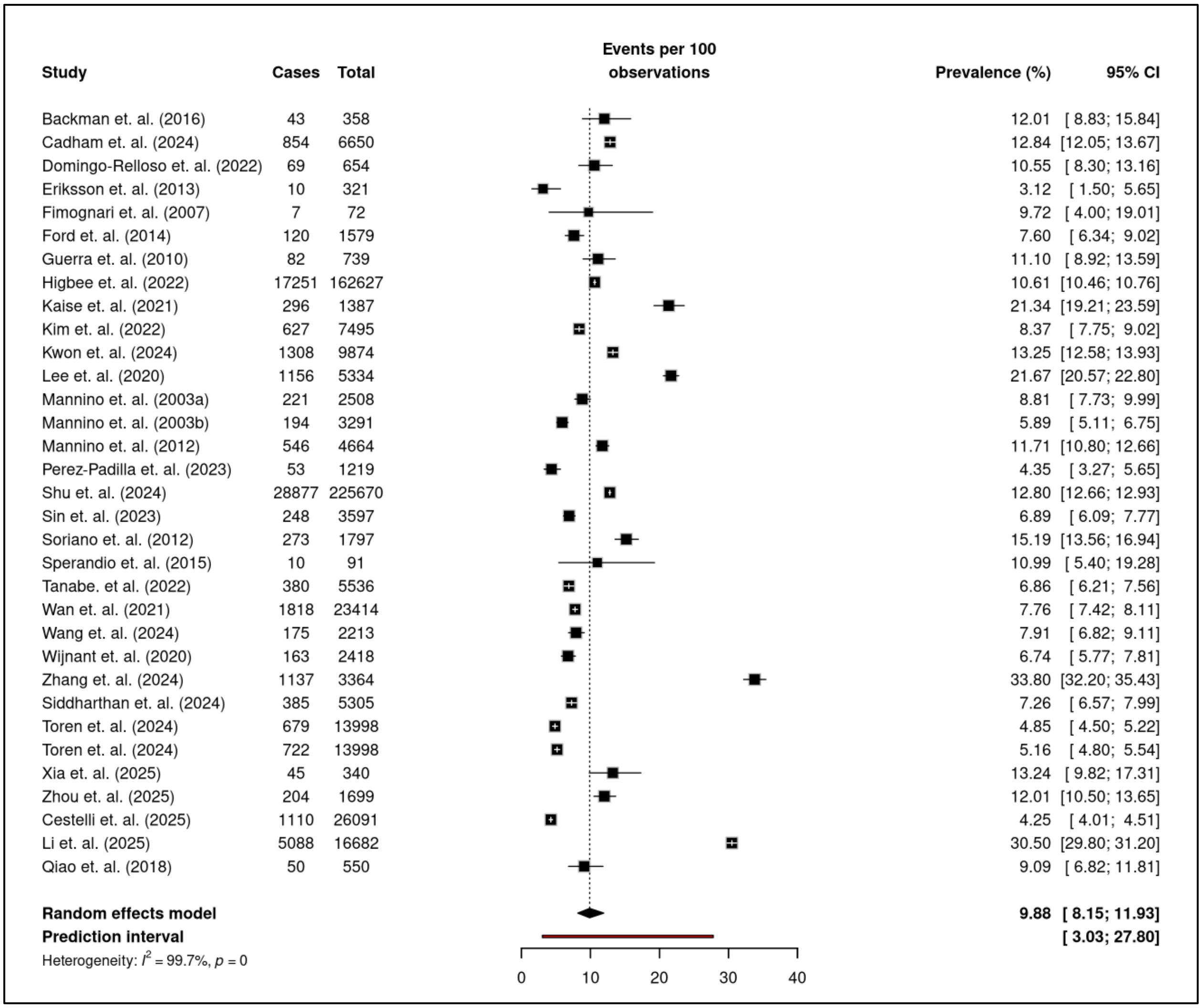

Figure 9.6: Combined prevalence of GOLD-PRISm and GOLD-RSP in males

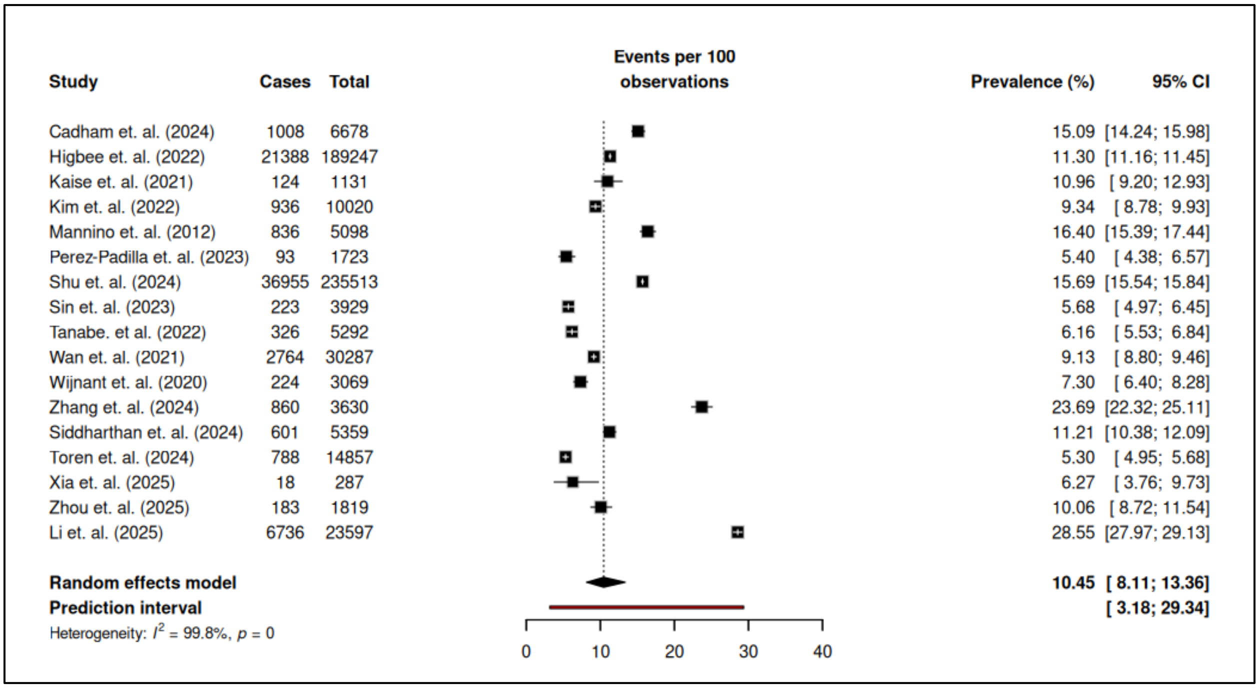

Figure 9.7: Prevalence of GOLD-PRISm in females

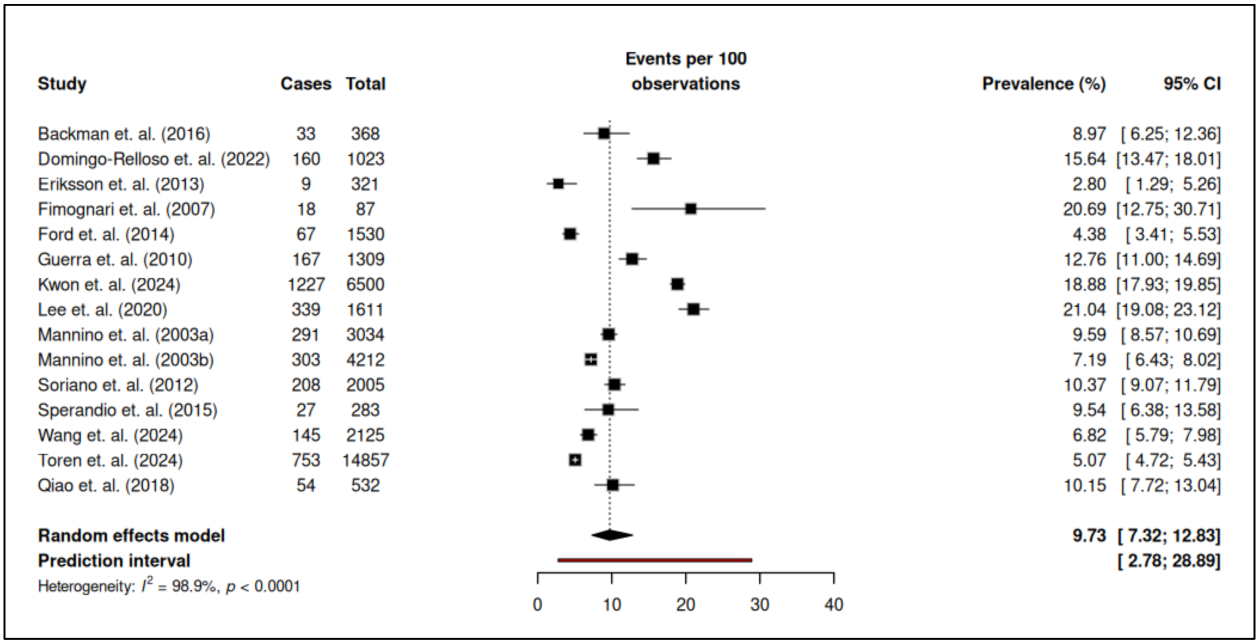

Figure 9.8: Prevalence of GOLD-RSP in females

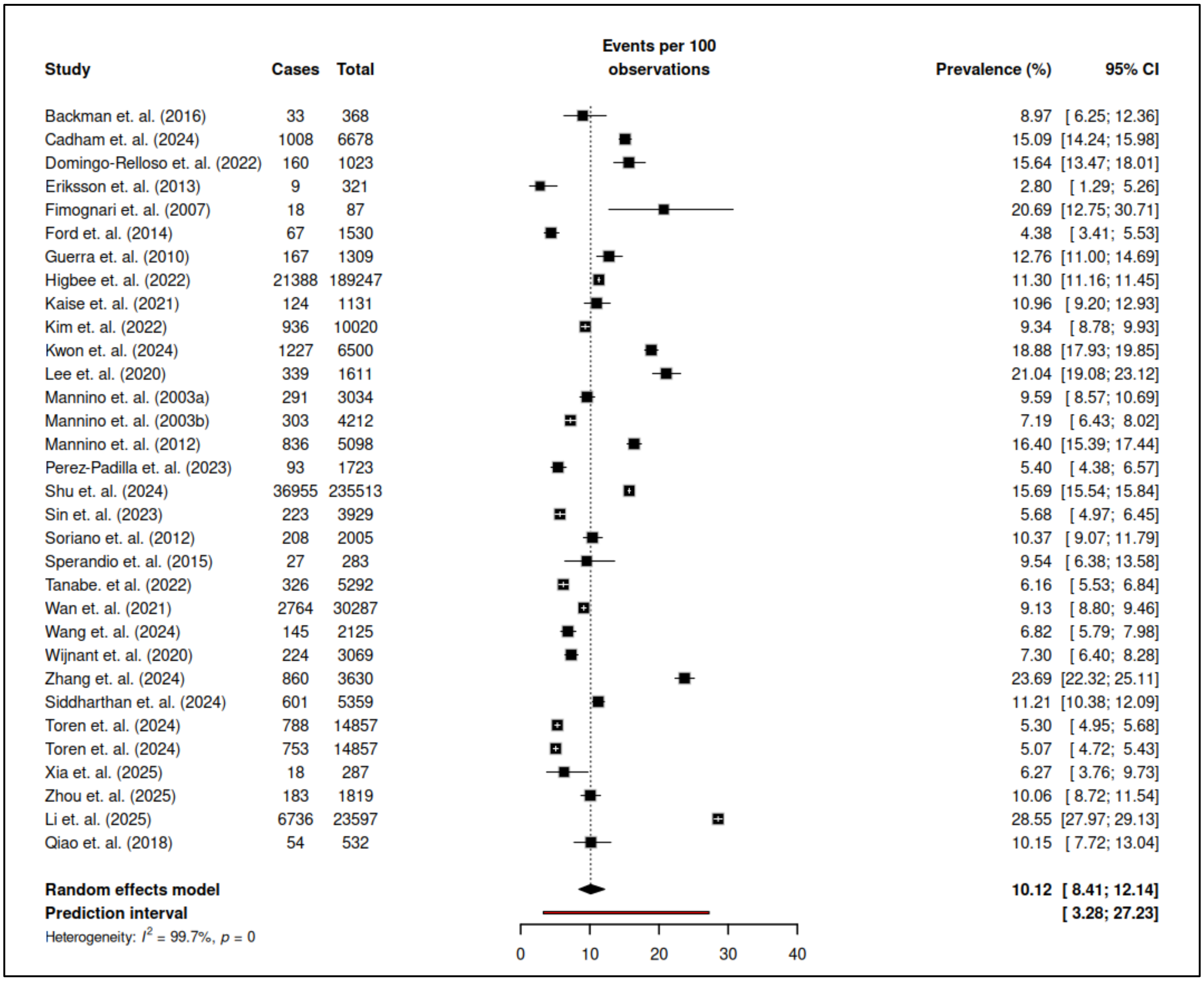

Figure 9.9: Combined prevalence of GOLD-PRISm and GOLD-RSP in females

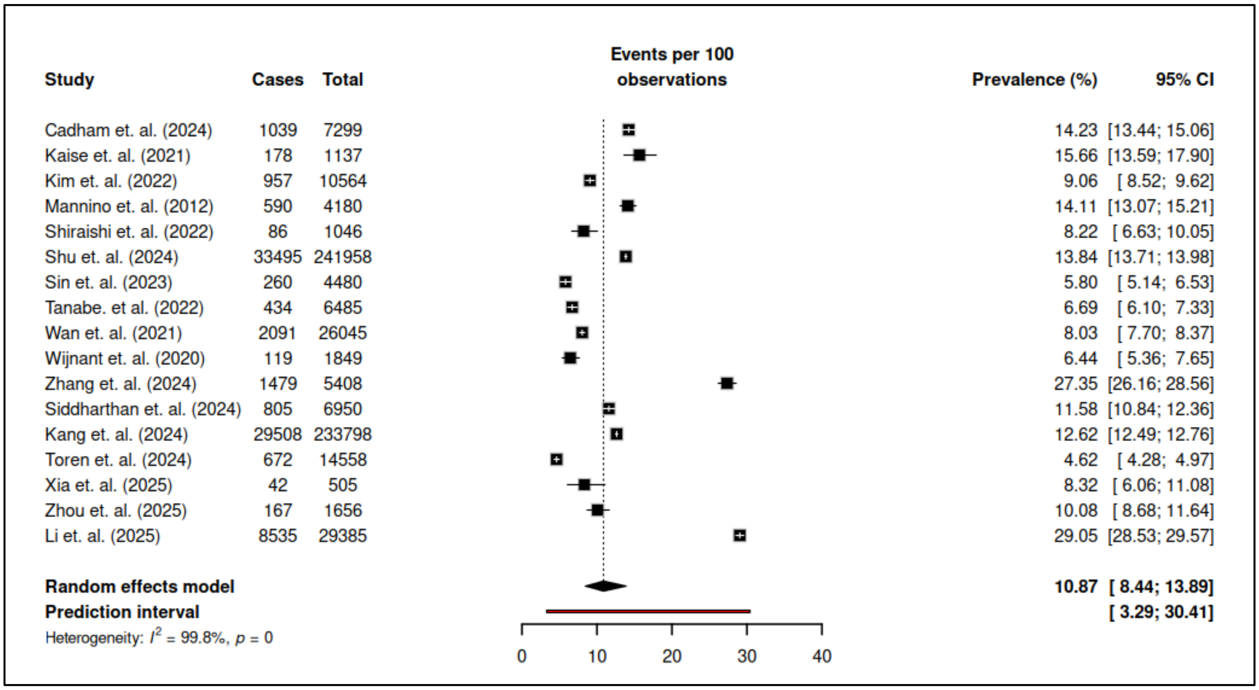

Figure 9.10: Prevalence of GOLD-PRISm in non-smokers

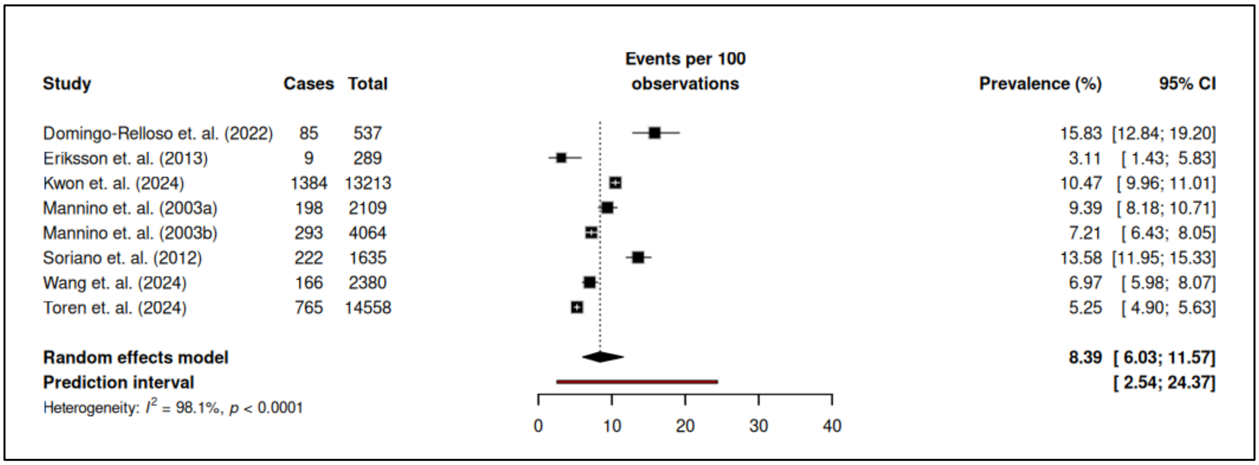

Figure 9.11: Prevalence of GOLD-RSP in non-smokers

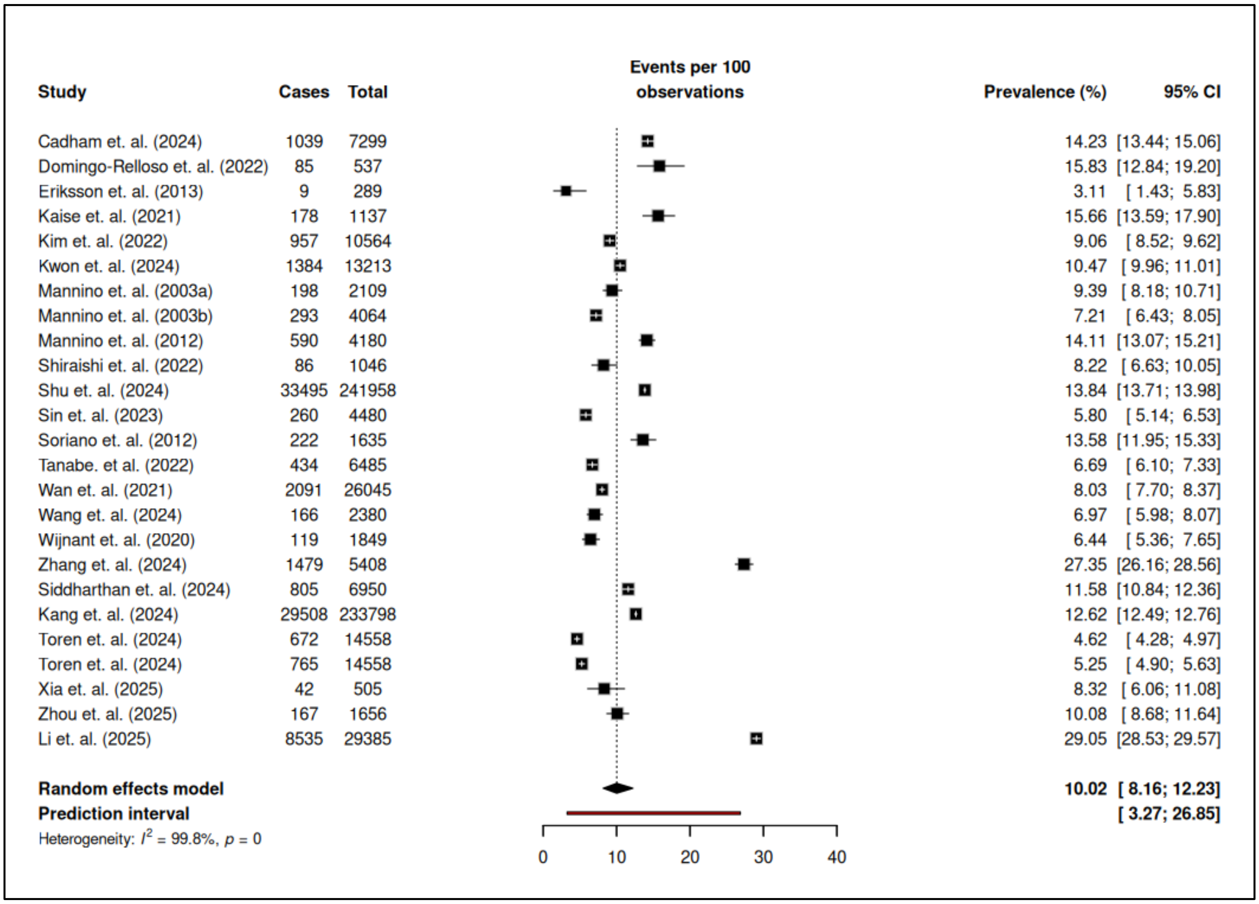

Figure 9.12: Combined prevalence of GOLD-PRISm and GOLD-RSP in non-smokers

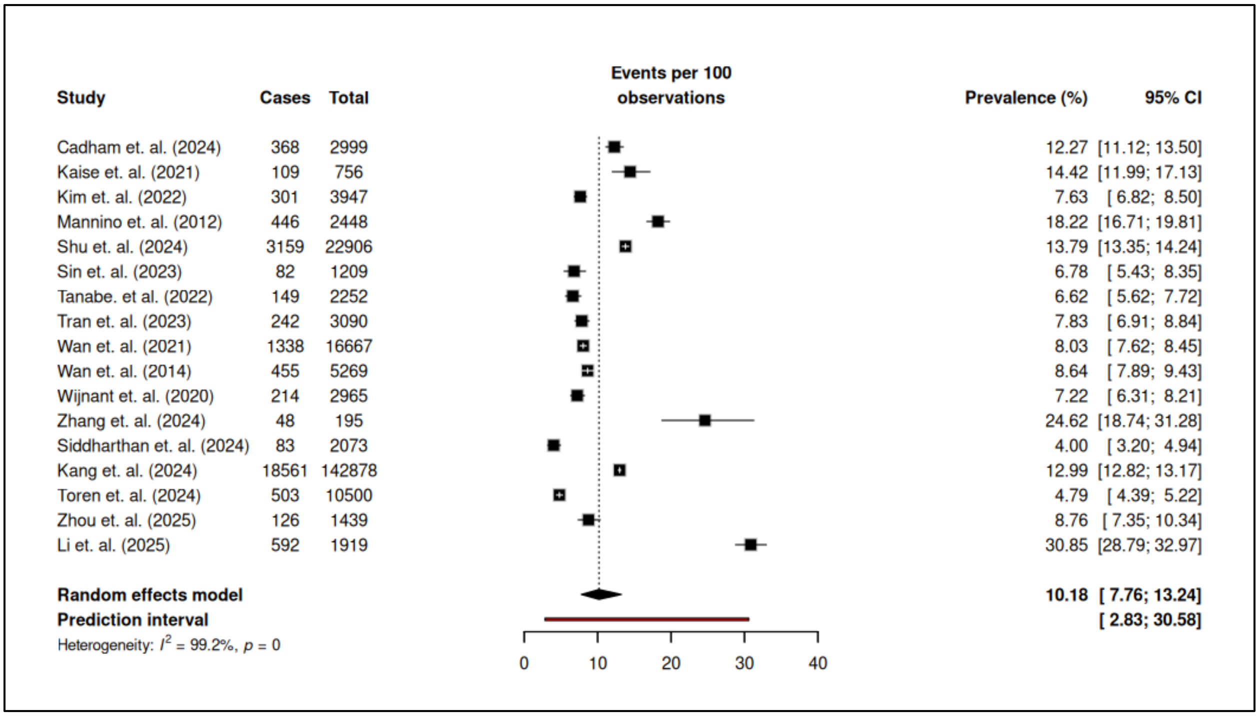

Figure 9.13: Prevalence of GOLD-PRISm in ex-smokers

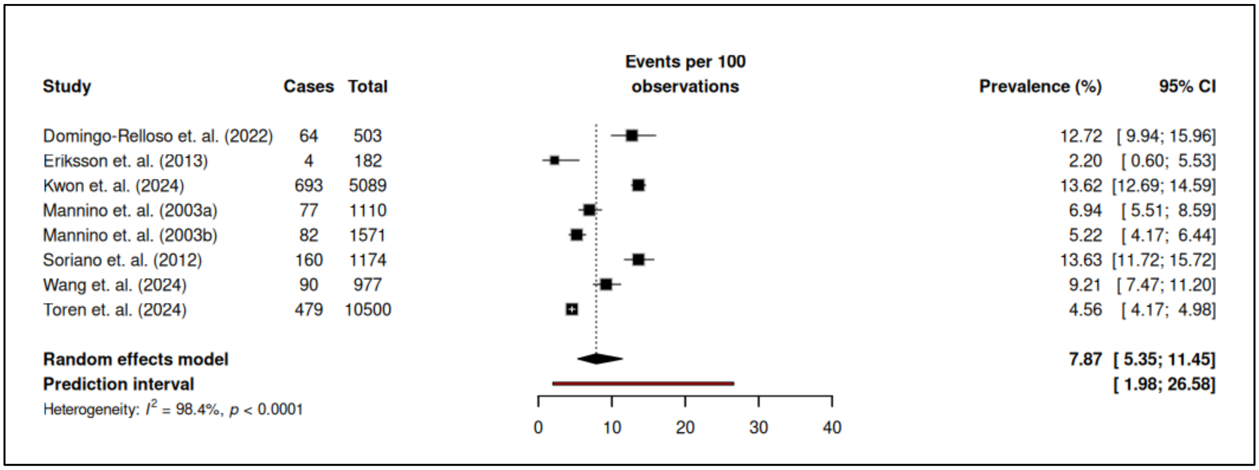

Figure 9.14: Prevalence of GOLD-RSP in ex-smokers

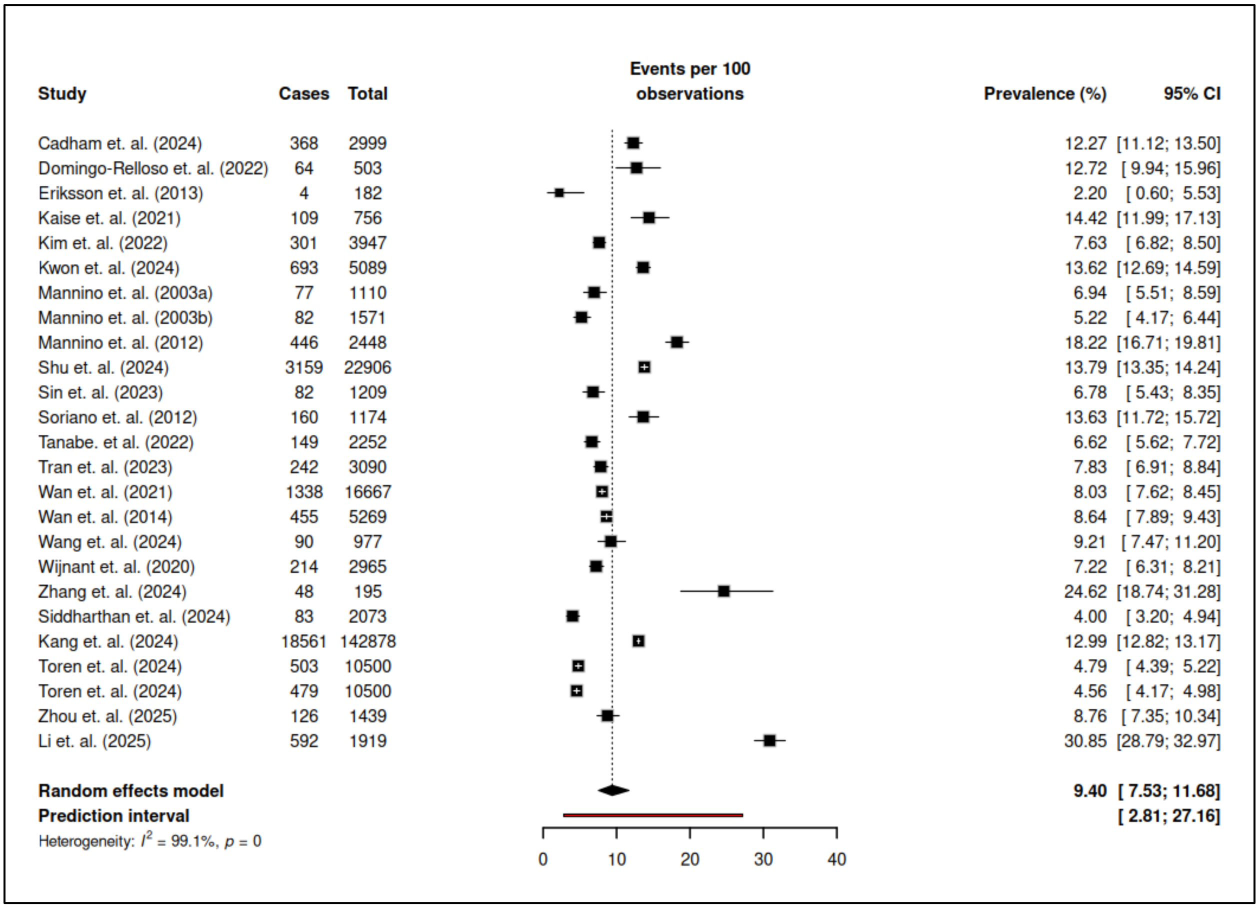

Figure 9.15: Combined prevalence of GOLD-PRISm and GOLD-RSP in ex-smokers

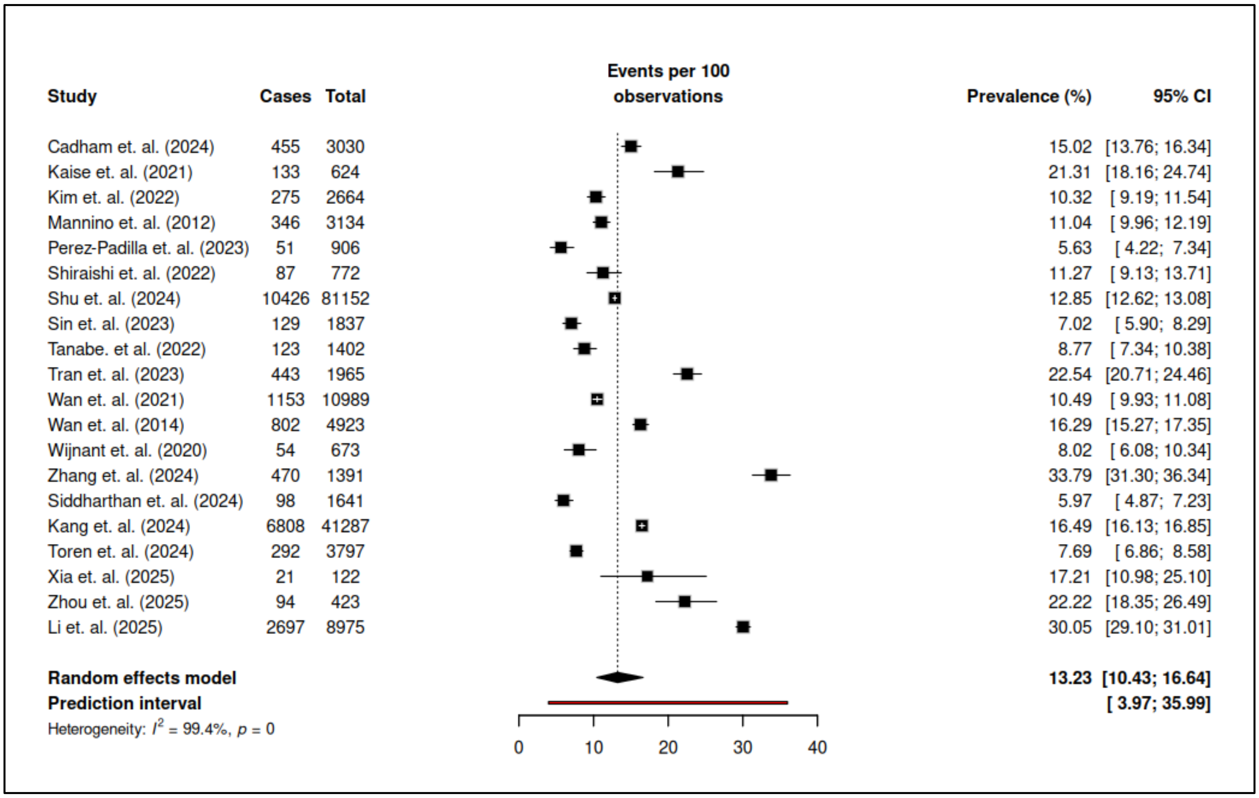

Figure 9.16: Prevalence of GOLD-PRISm in current smokers

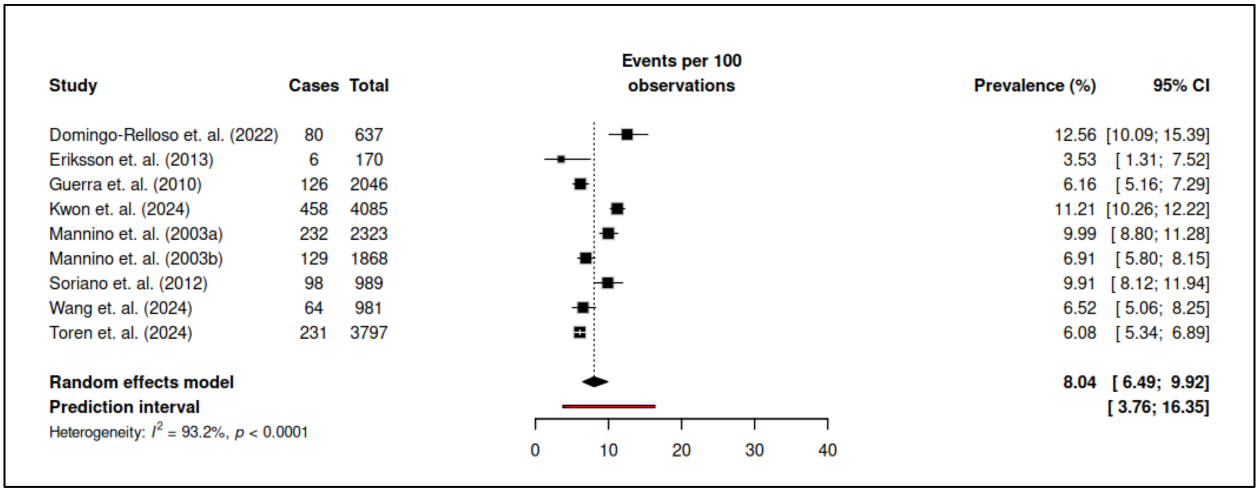

Figure 9.17: Prevalence of GOLD-RSP in current smokers

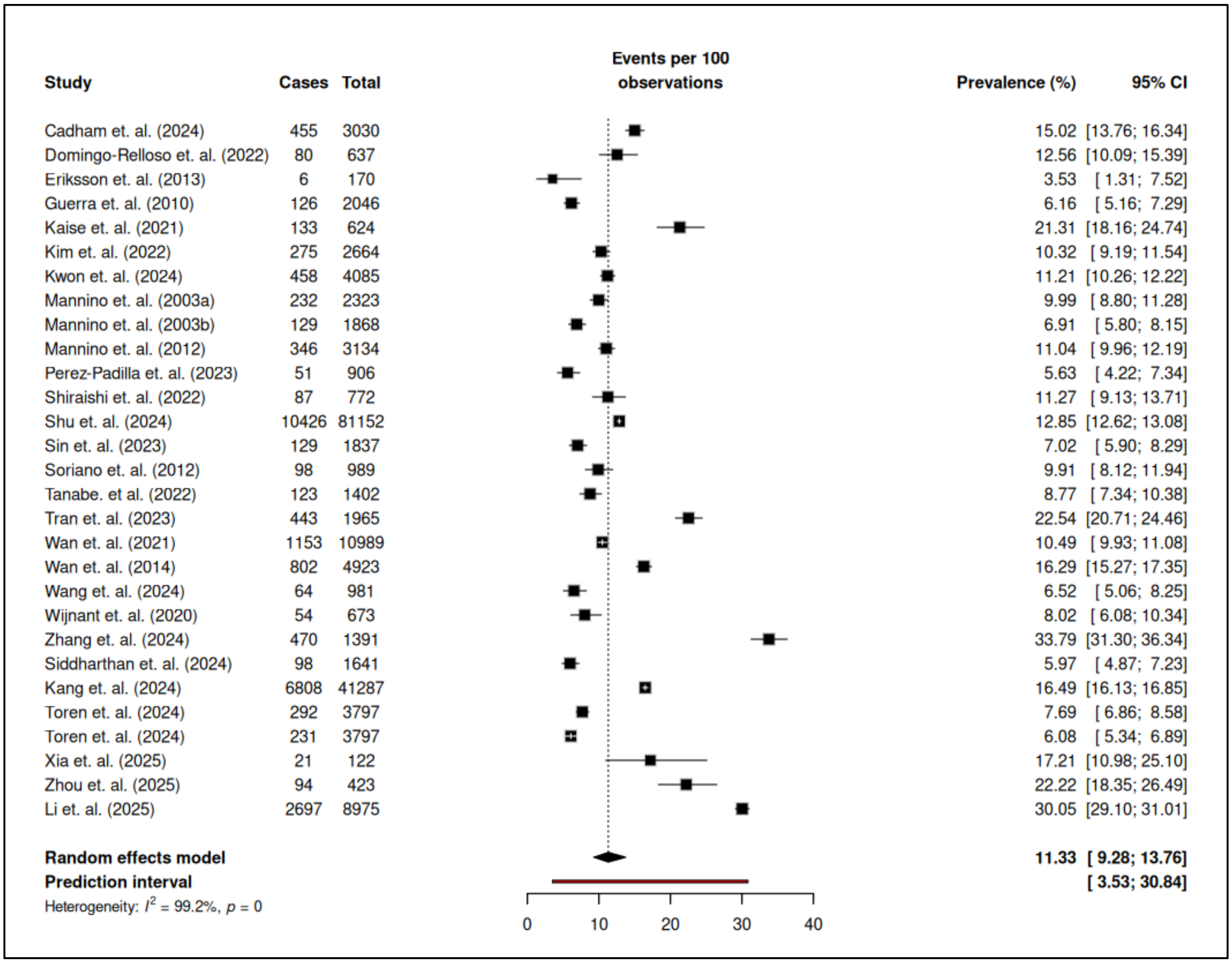

Figure 9.18: Combined prevalence of GOLD-PRISm and GOLD-RSP in current smokers

Supplement S10: Forest plots – Risk factors (main and supplementary analysis)

10.1 GOLD studies (main analysis)

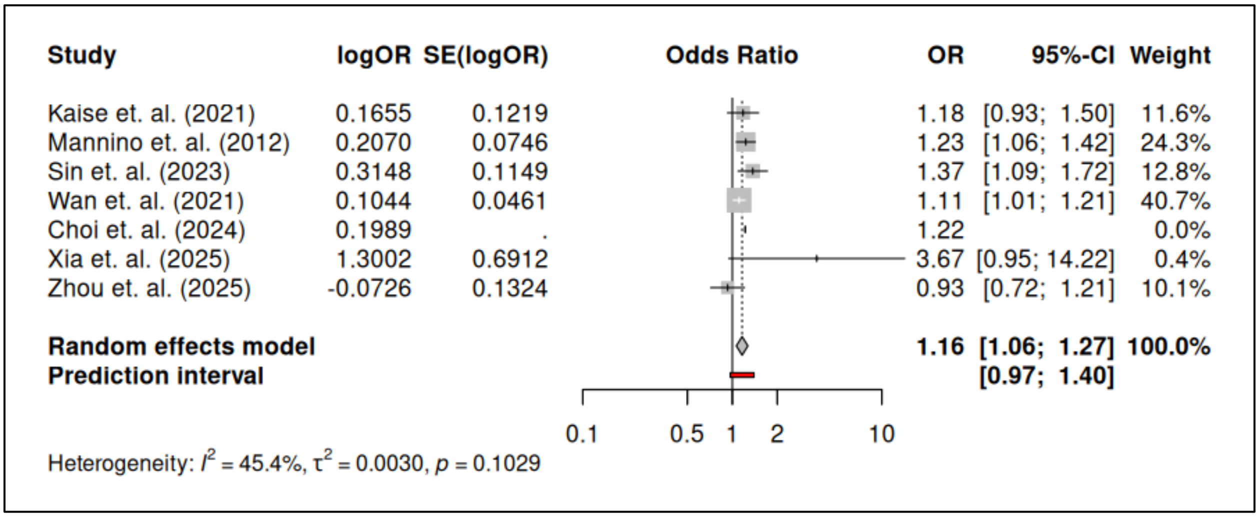

Figure 10.1.1: Pooled univariable odds ratio of age group 50-59 years (vs 40-49 years) for GOLD-PRISm

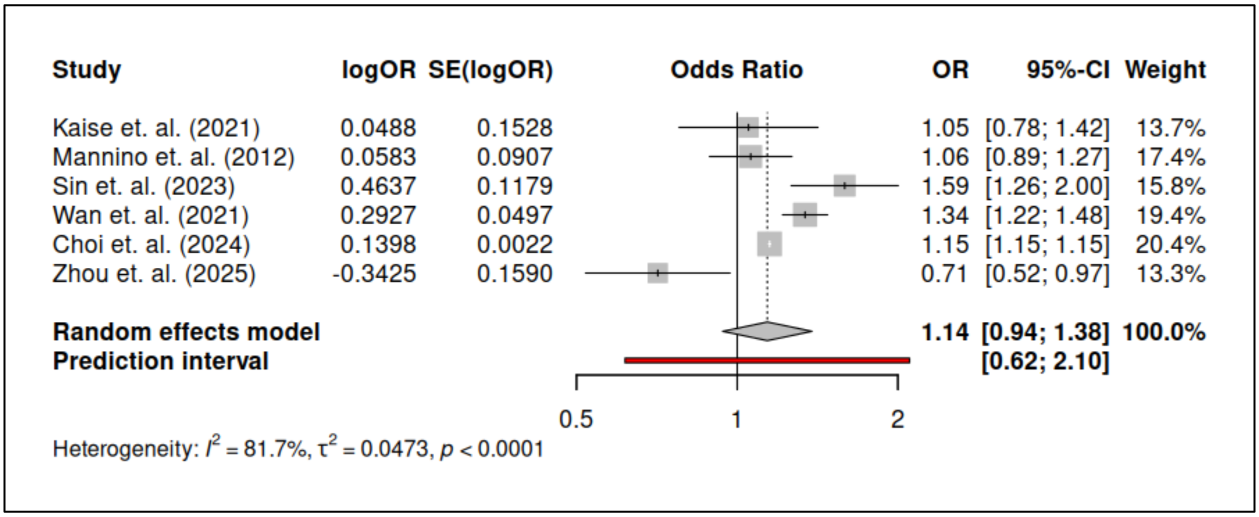

Figure 10.1.2: Pooled univariable odds ratio of age group 60-69 years (vs 40-49 years) for GOLD-PRISm

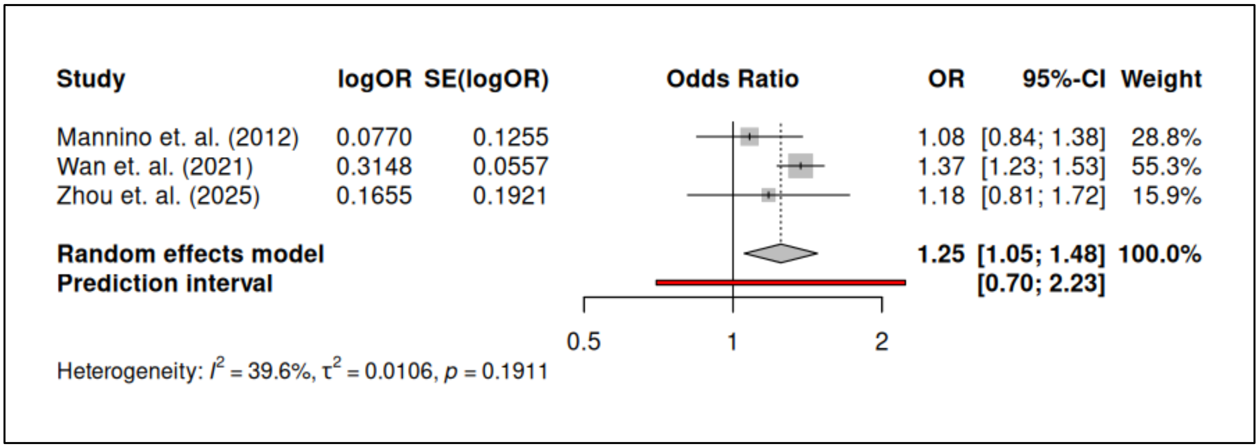

Figure 10.1.3: Pooled univariable odds ratio of age group 70-79 years (vs 40-49 years) for GOLD-PRISm

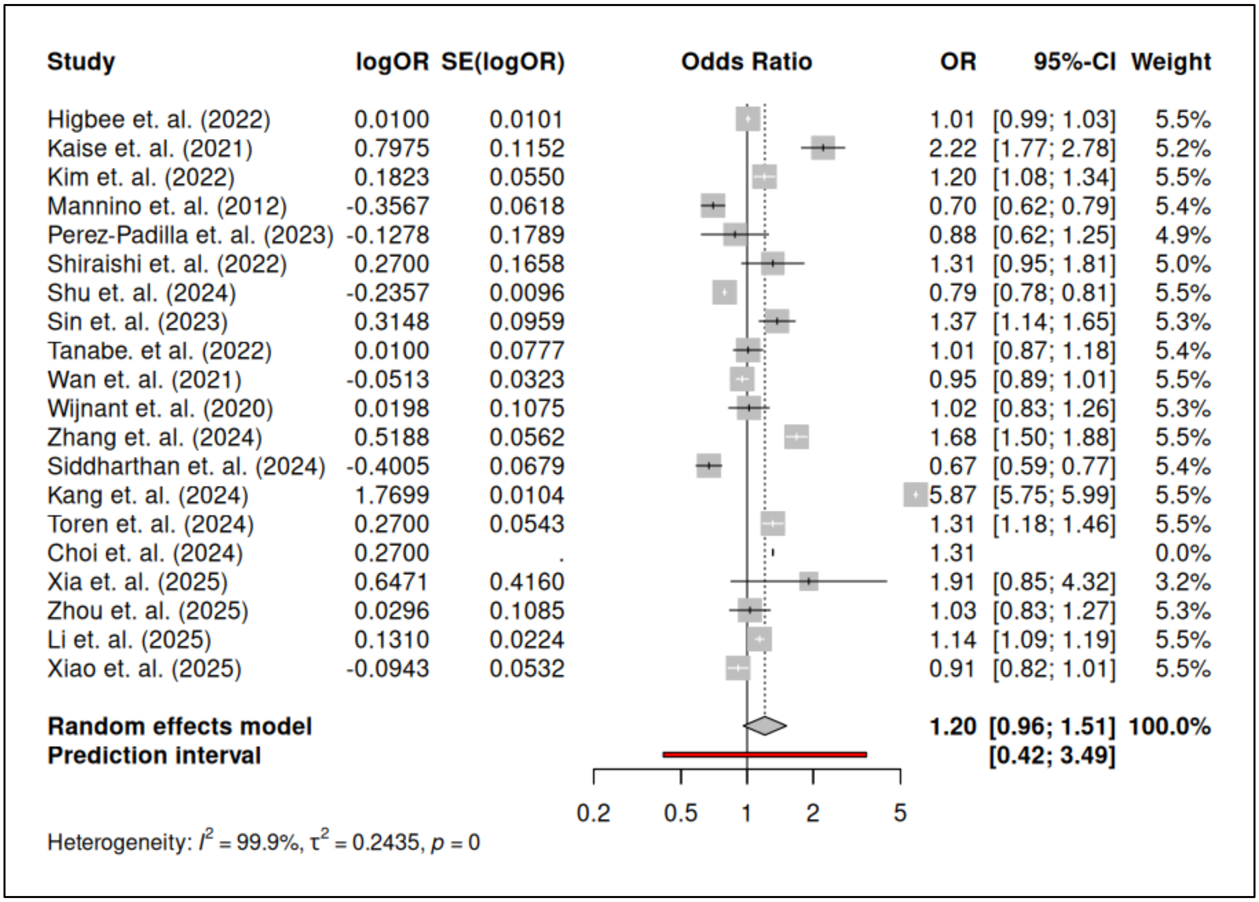

Figure 10.1.4: Pooled univariable odds ratio of males (vs females) for GOLD-PRISm

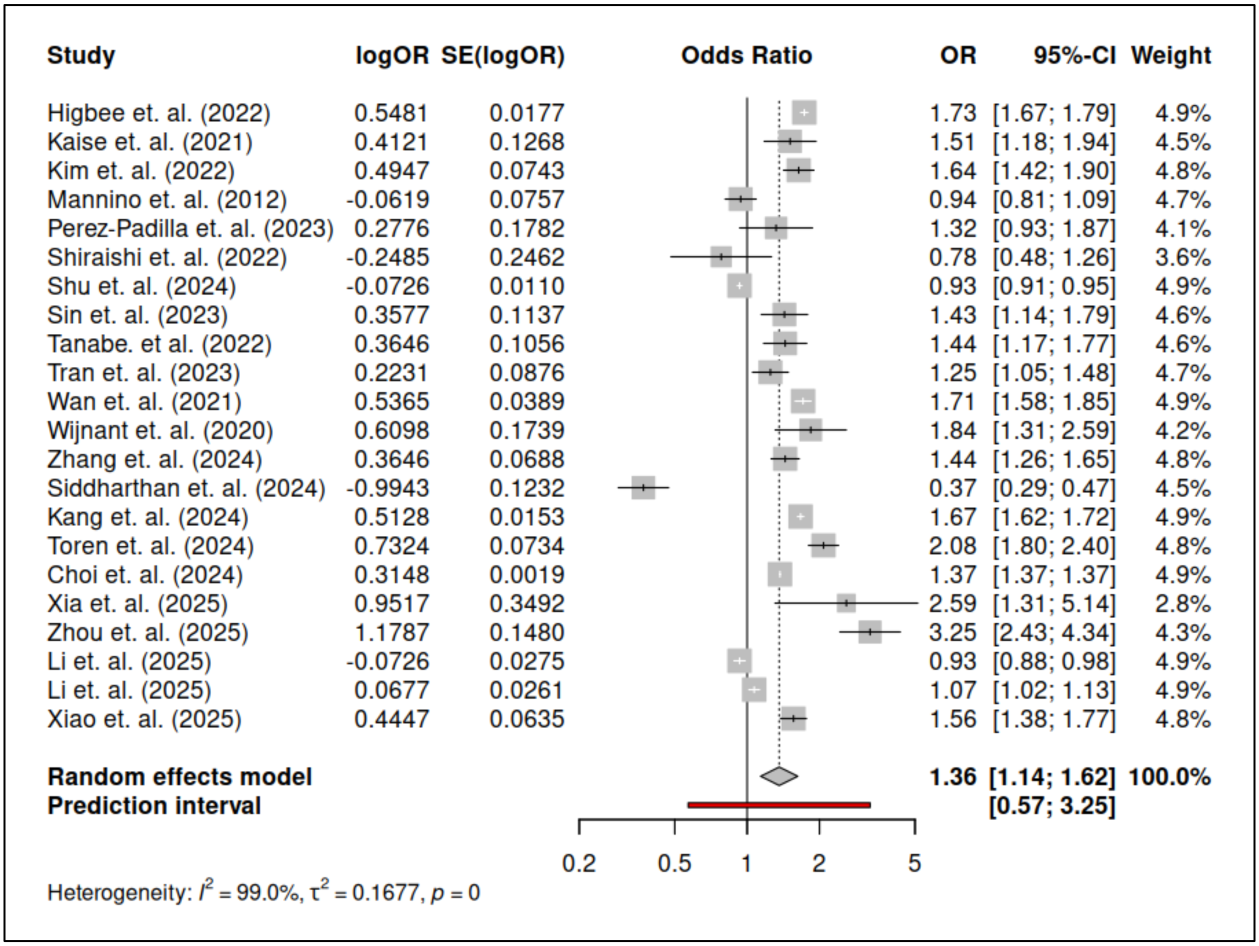

Figure 10.1.5: Pooled univariable odds ratio of current smokers (vs non-smokers) for GOLD-PRISm

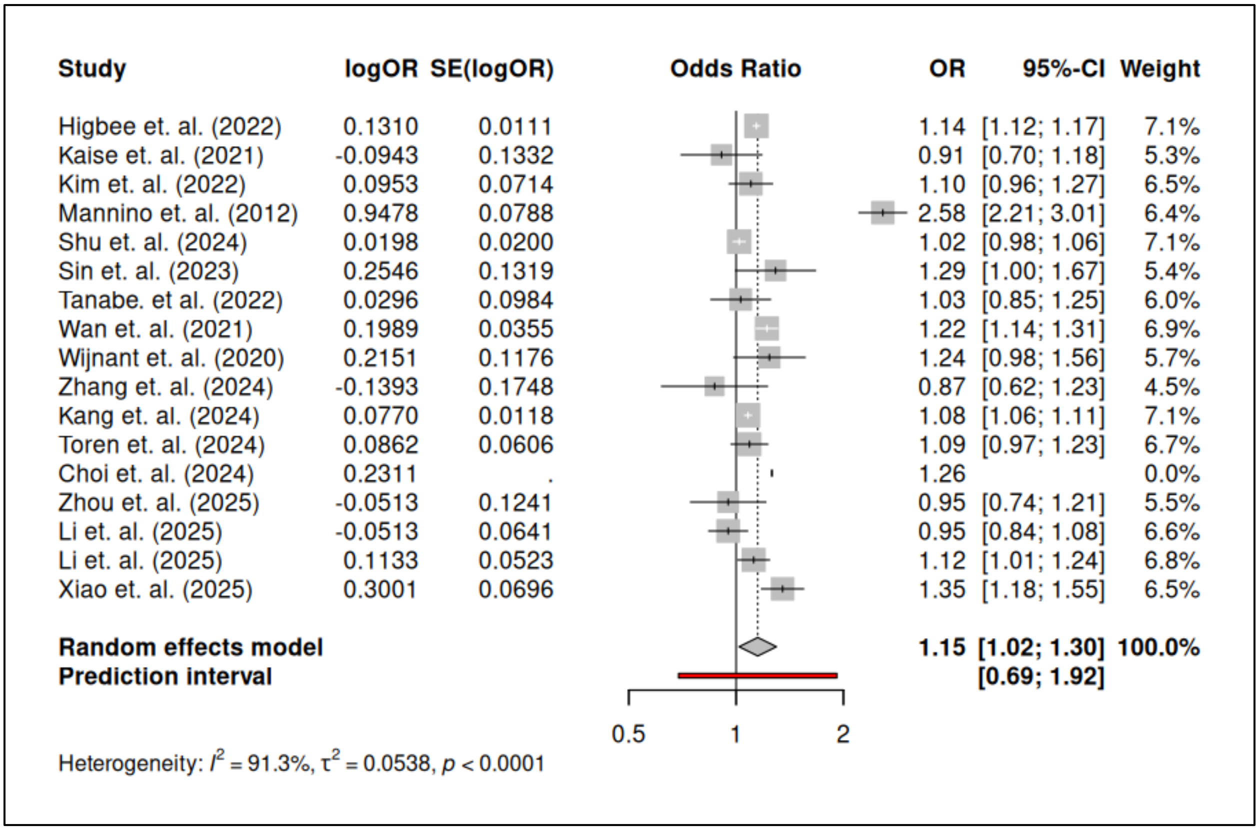

Figure 10.1.6: Pooled univariable odds ratio of former smokers (vs non-smokers) for GOLD-PRISm

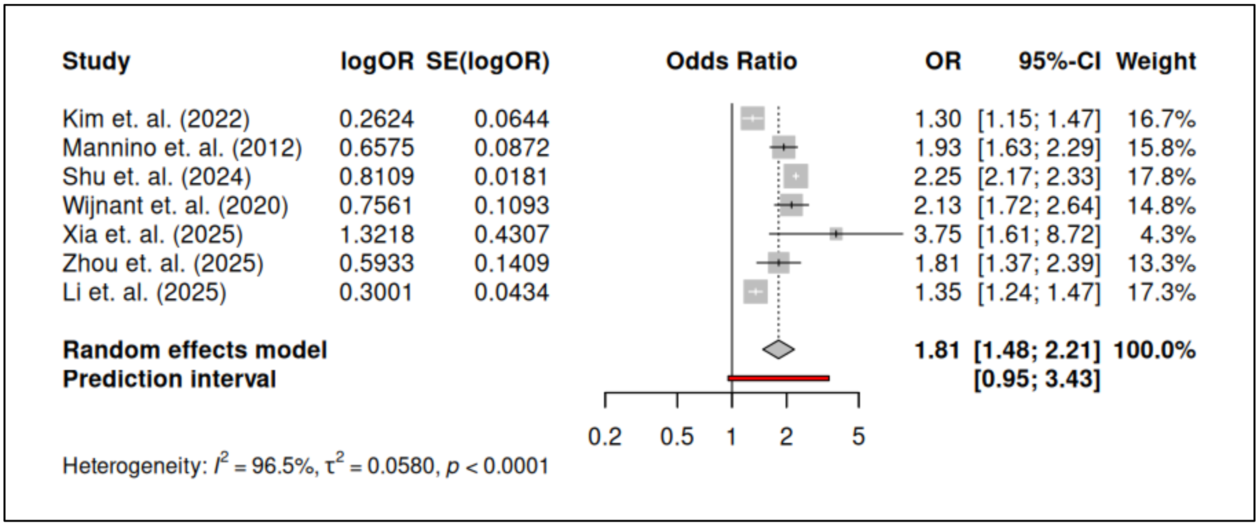

Figure 10.1.7: Pooled univariable odds ratio of obese (≥30 kg/m^2) BMI (vs normal 18.5-24.9 kg/m^2 BMI) for GOLD-PRISm

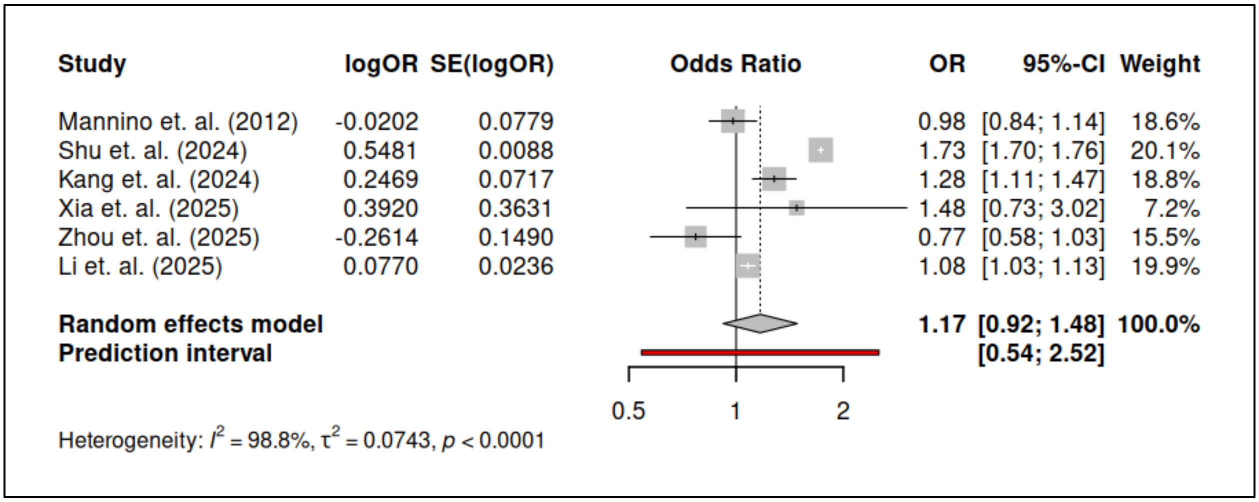

Figure 10.1.8: Pooled univariable odds ratio of overweight (25-29.9 kg/m^2) BMI (vs normal 18.5-24.9 kg/m^2 BMI) for GOLD-PRISm

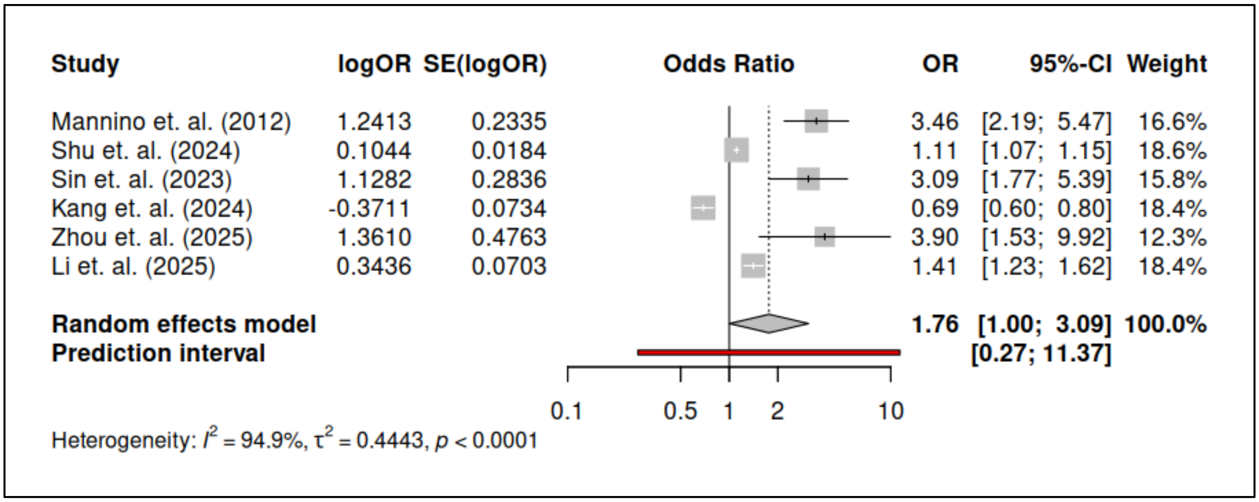

Figure 10.1.9: Pooled univariable odds ratio of underweight (<18.5 kg/m^2) (vs normal 18.5-24.9 kg/m^2 BMI) for GOLD-PRISm

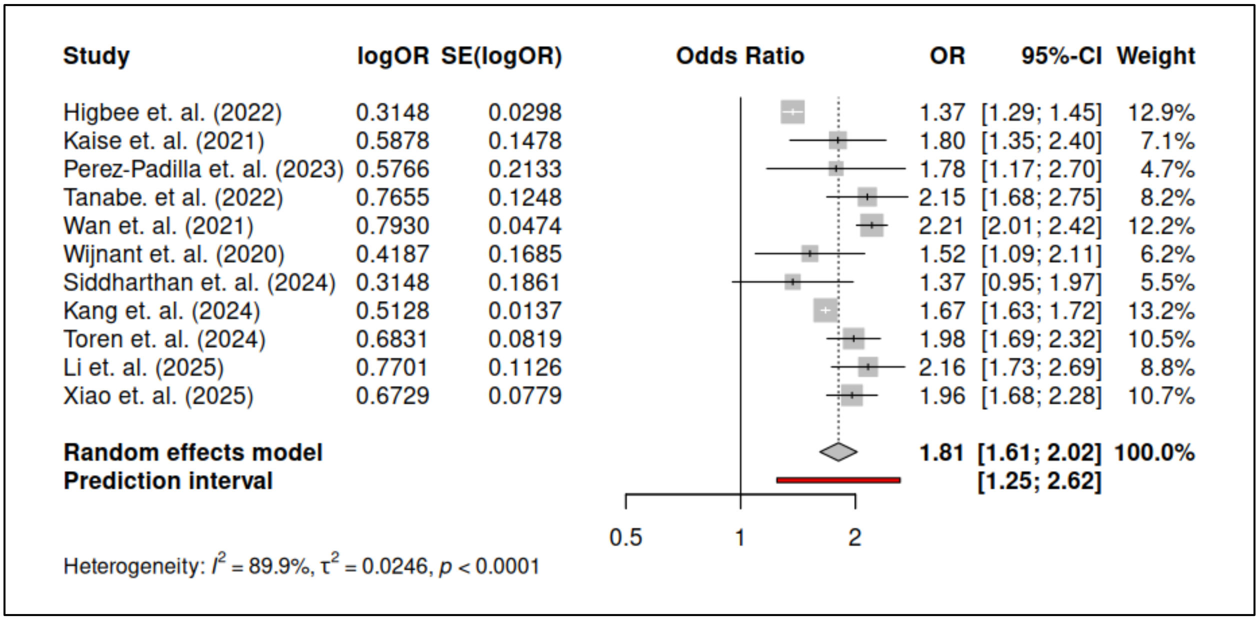

Figure 10.1.10: Pooled univariable odds ratio of history of asthma for GOLD-PRISm

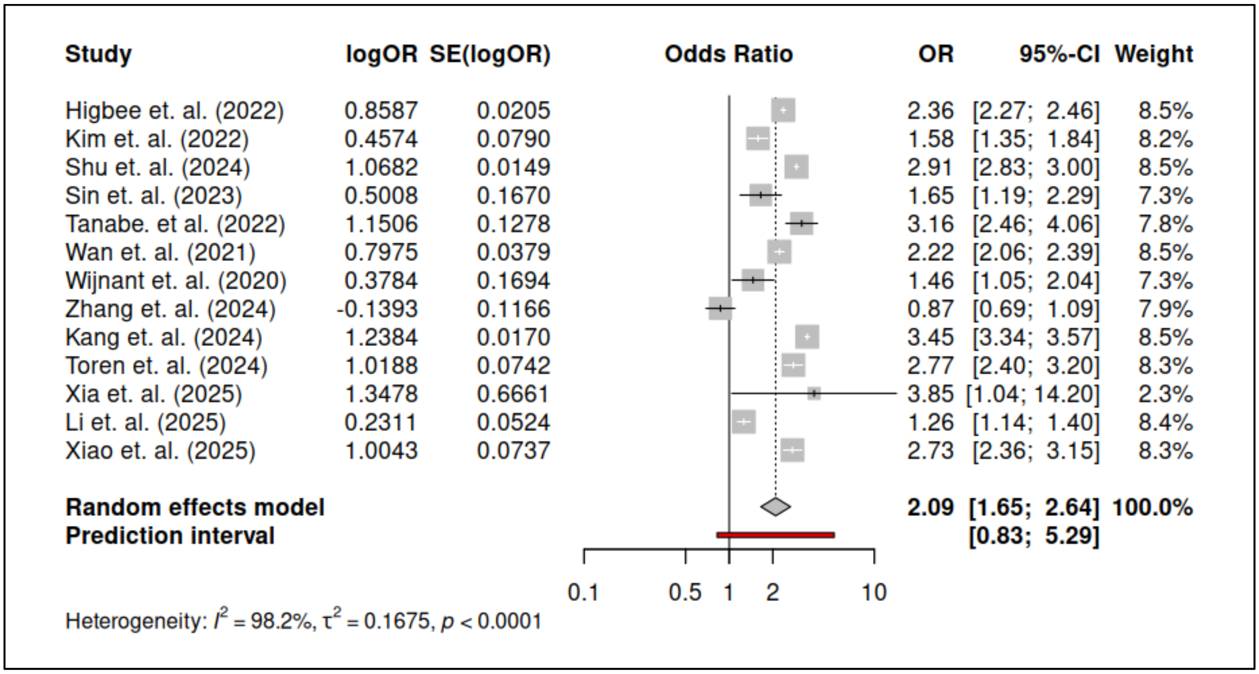

Figure 10.1.11: Pooled univariable odds ratio of history of diabetes for GOLD-PRISm

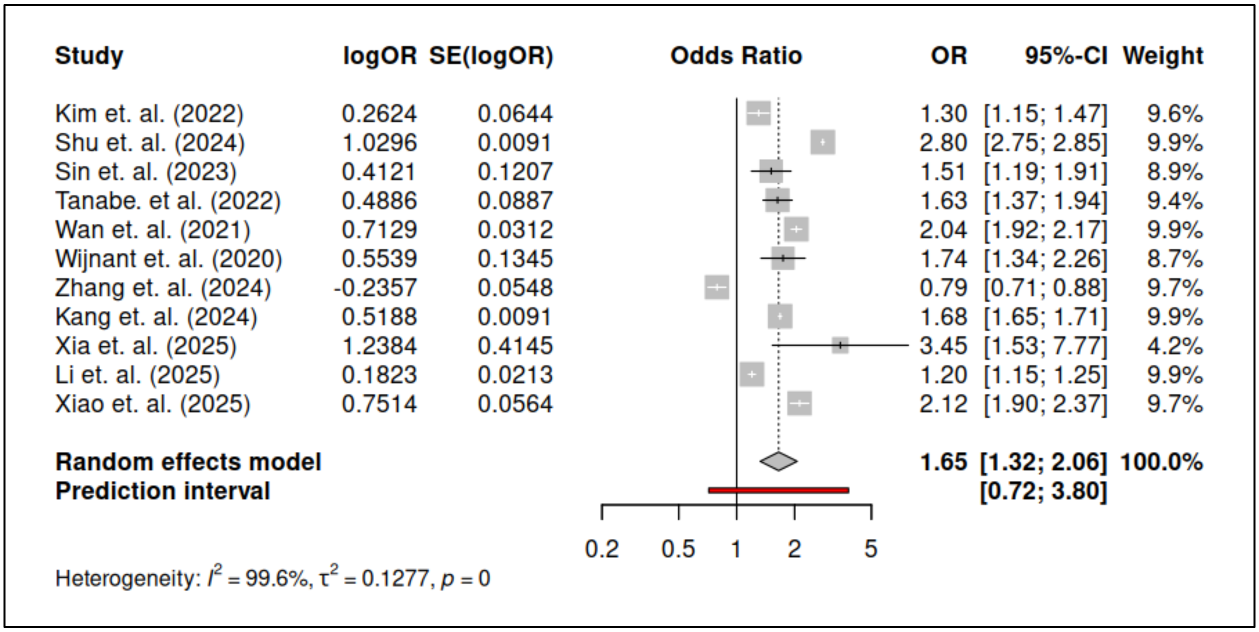

Figure 10.1.12: Pooled univariable odds ratio of history of hypertension for GOLD-PRISm

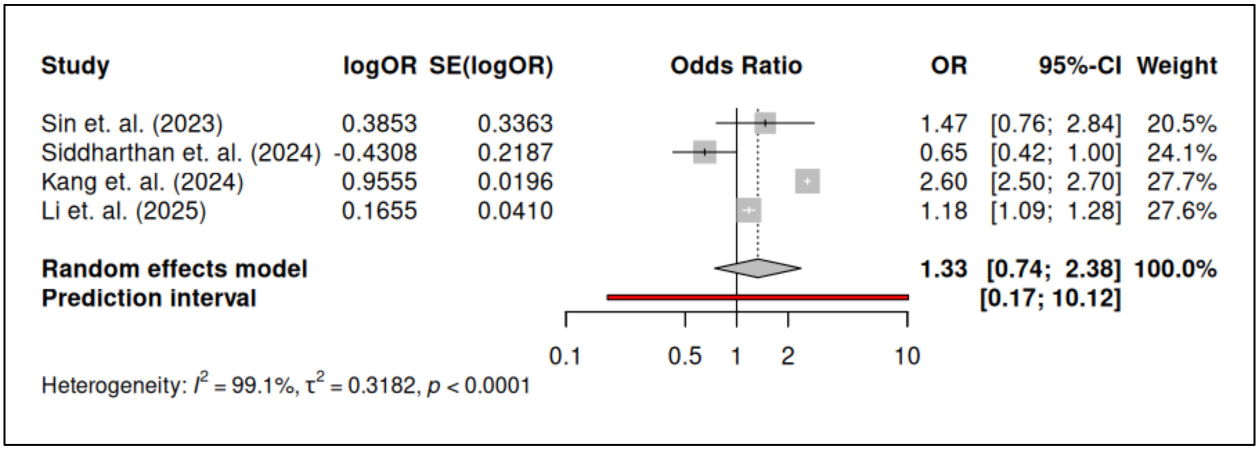

Figure 10.1.13: Pooled univariable odds ratio of history of cardiovascular disease for GOLD-PRISm

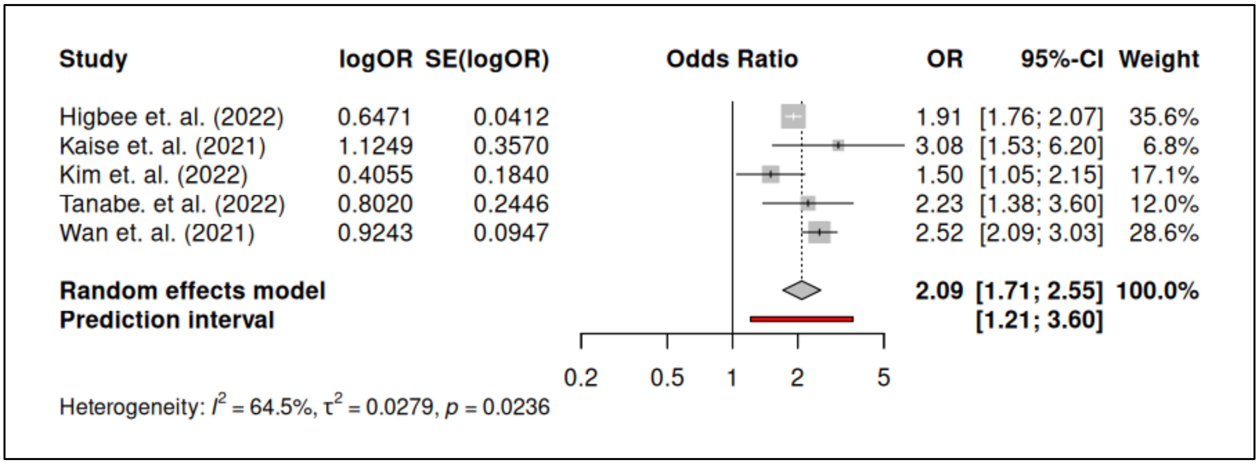

Figure 10.1.14: Pooled univariable odds ratio of history of stroke for GOLD-PRISm

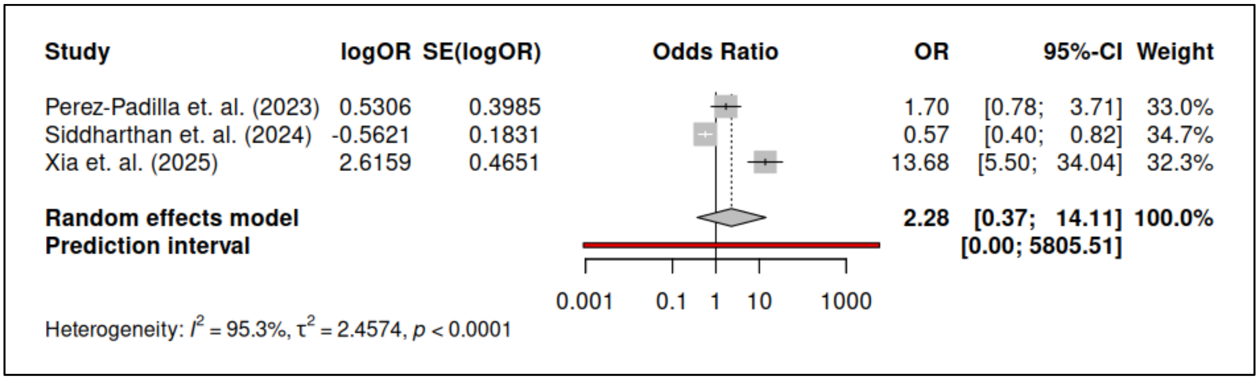

Figure 10.1.15: Pooled univariable odds ratio of history of tuberculosis for GOLD-PRISm

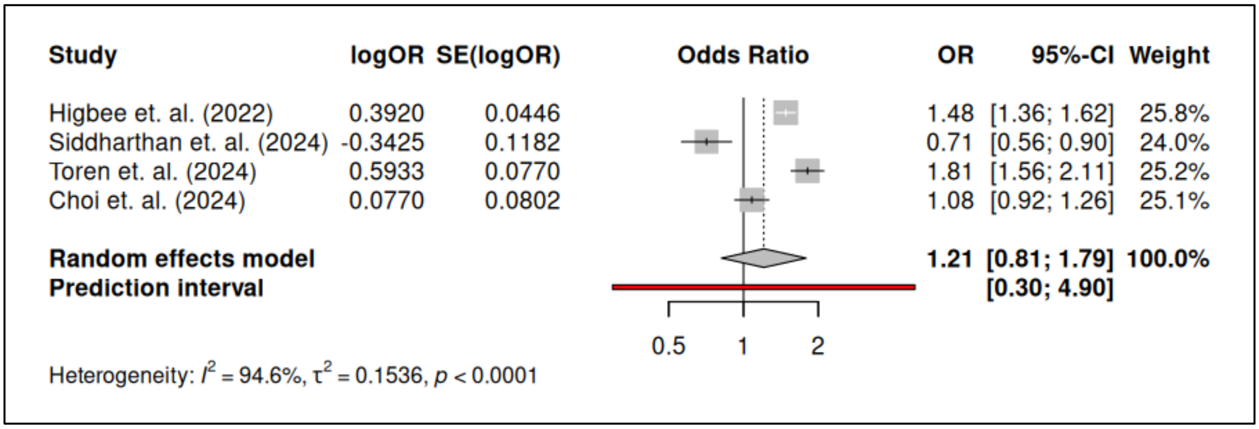

Figure 10.1.16: Pooled multivariable odds ratio of current smokers (vs non-smokers) for GOLD-PRISm

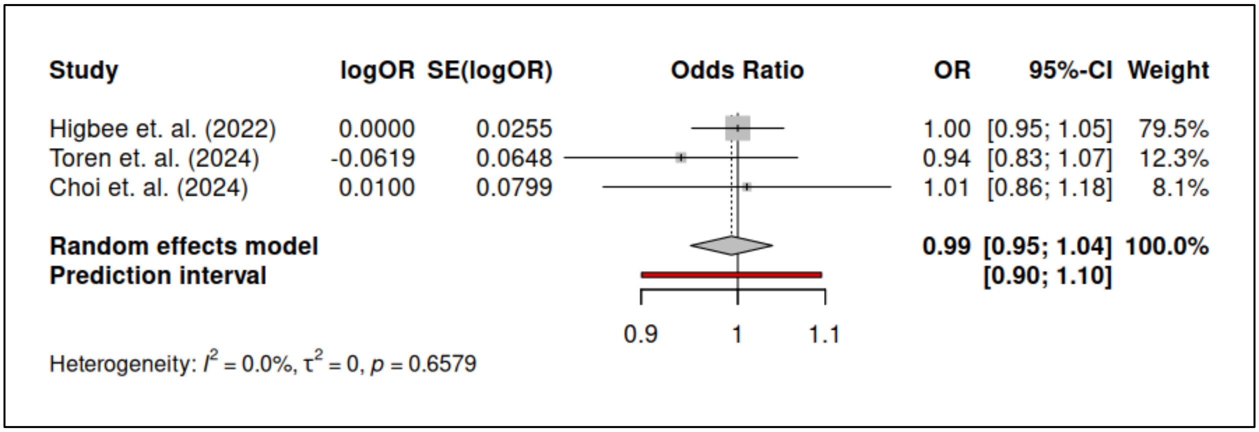

Figure 10.1.17: Pooled multivariable odds ratio of former smokers (vs non-smokers) for GOLD-PRISm

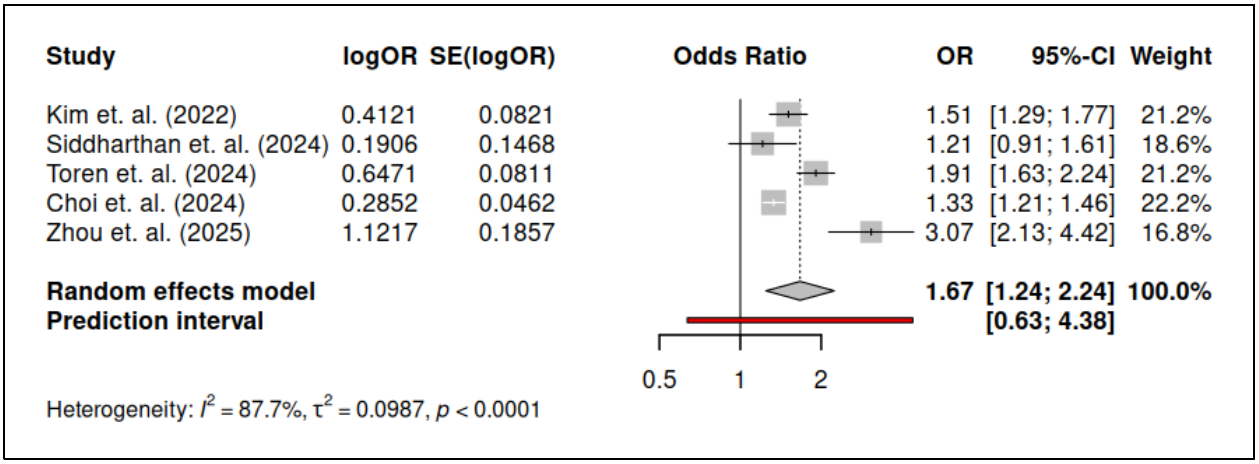

Figure 10.1.18: Pooled multivariable odds ratio of history of diabetes for GOLD-PRISm

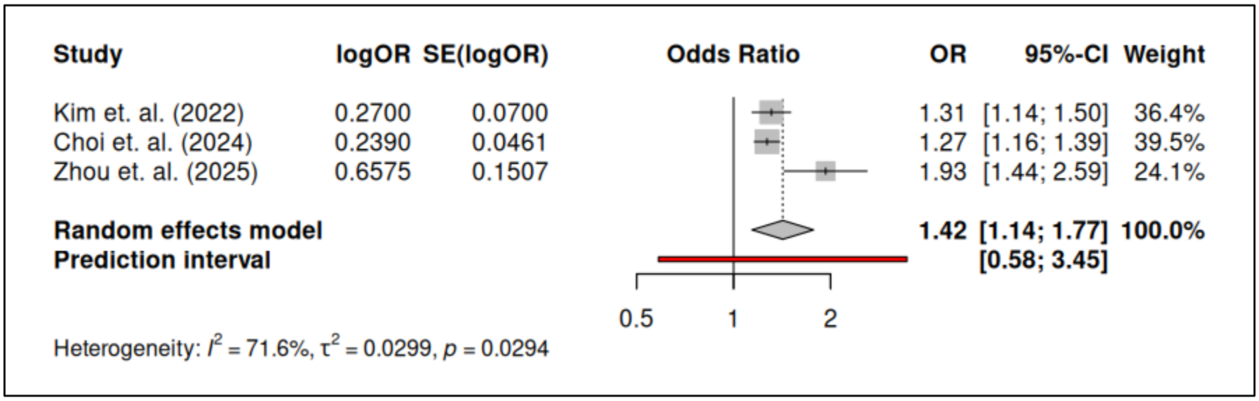

Figure 10.1.19: Pooled multivariable odds ratio of history of hypertension for GOLD-PRISm

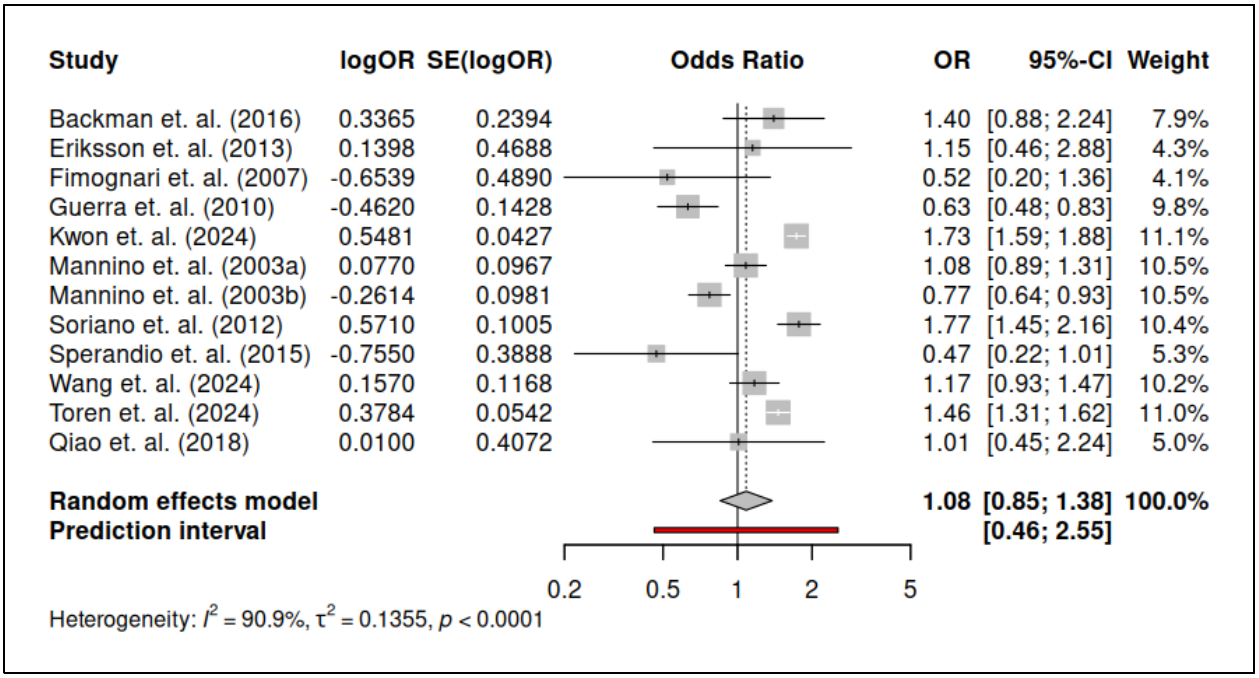

Figure 10.1.20: Pooled univariable odds ratio of males (vs females) for GOLD-RSP

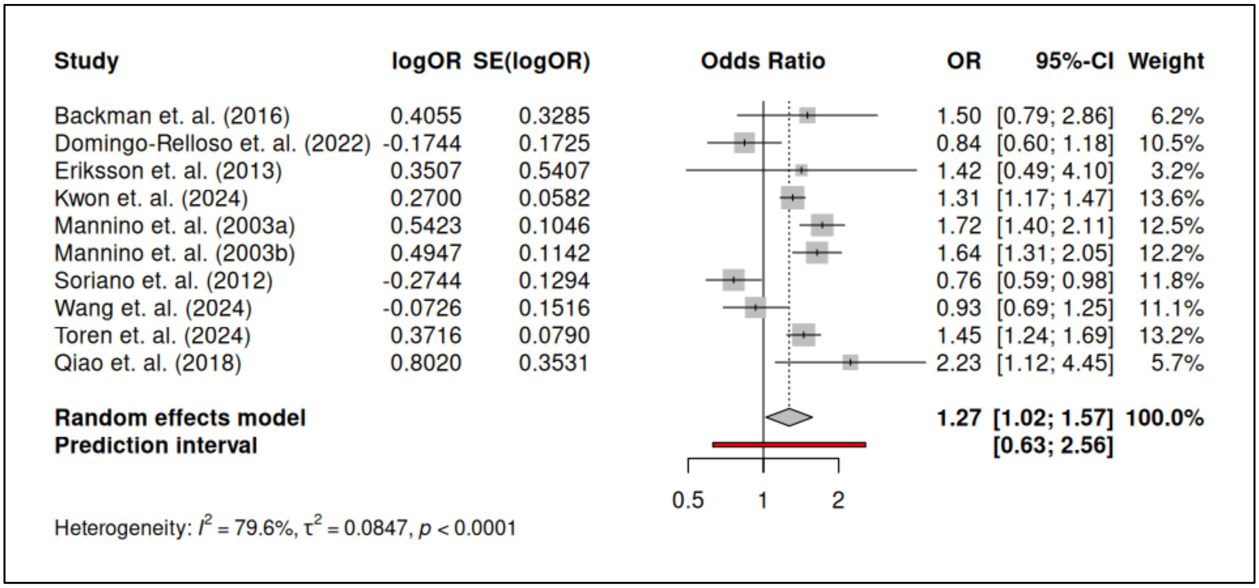

Figure 10.1.21: Pooled univariable odds ratio of current smokers (vs non-smokers) for GOLD-RSP

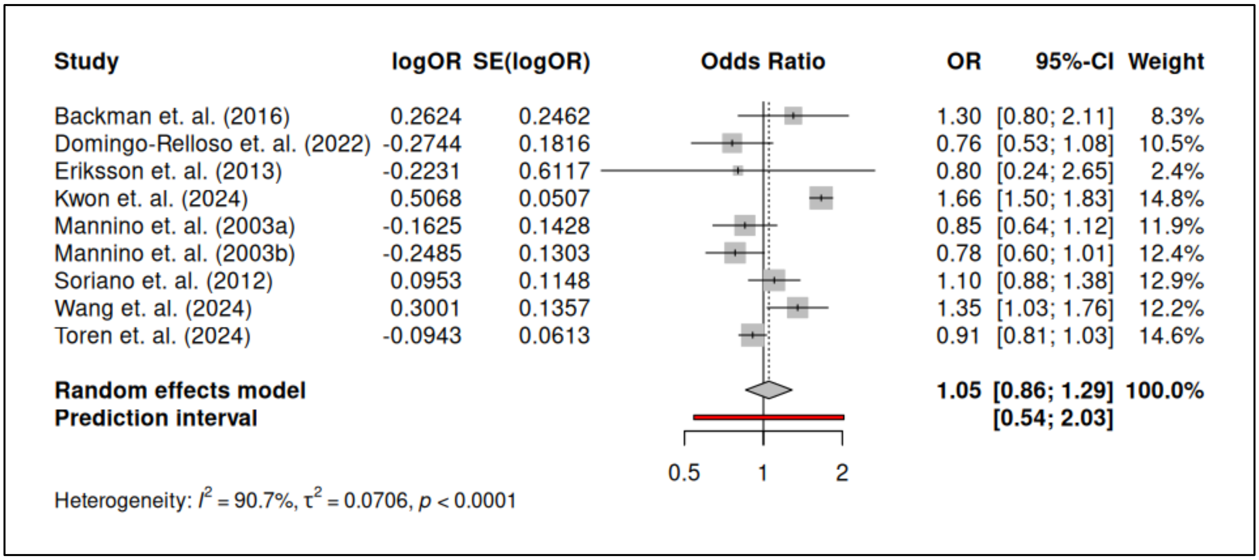

Figure 10.1.22: Pooled univariable odds ratio of former smokers (vs non-smokers) for GOLD-RSP

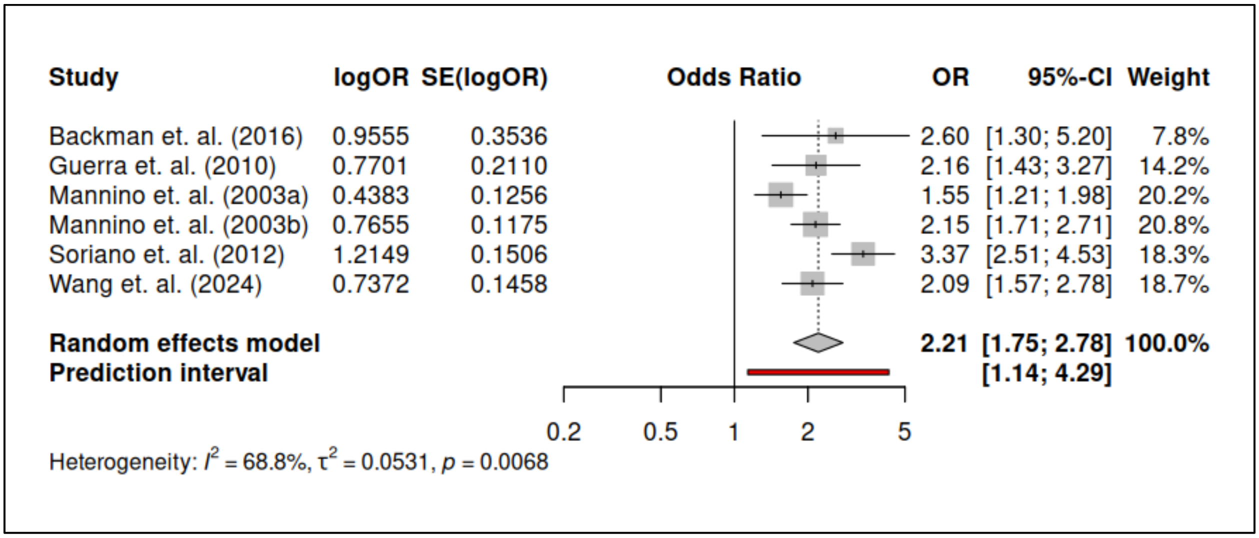

Figure 10.1.23: Pooled univariable odds ratio of obese (≥30 kg/m^2) BMI (vs normal 18.5-24.9 kg/m^2 BMI) for GOLD-RSP

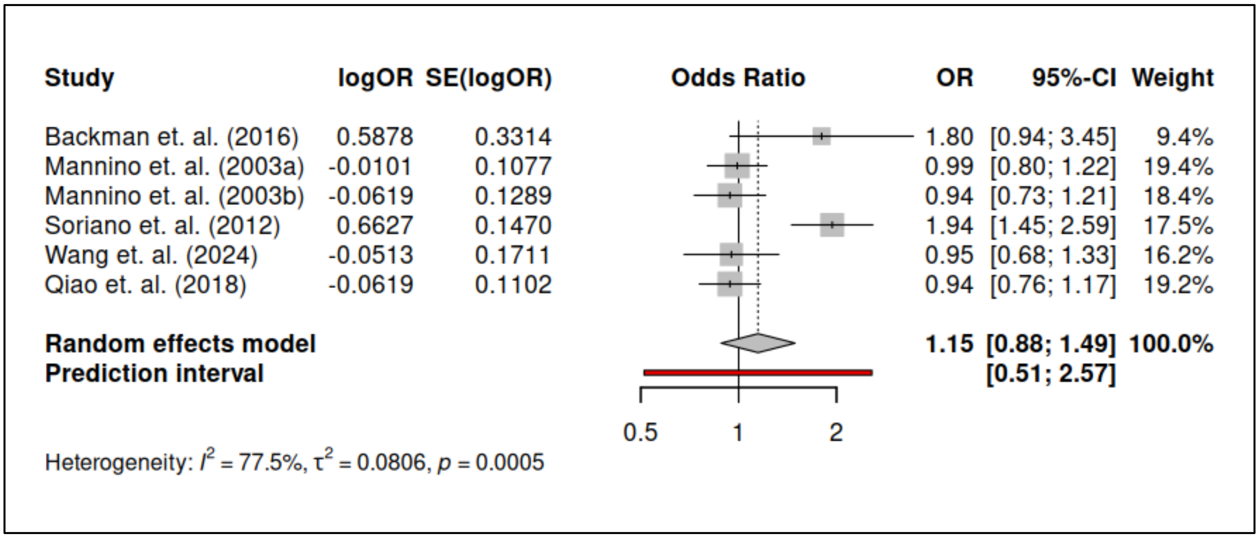

Figure 10.1.24: Pooled univariable odds ratio of overweight (25-29.9 kg/m^2) BMI (vs normal 18.5-24.9 kg/m^2 BMI) for GOLD-RSP

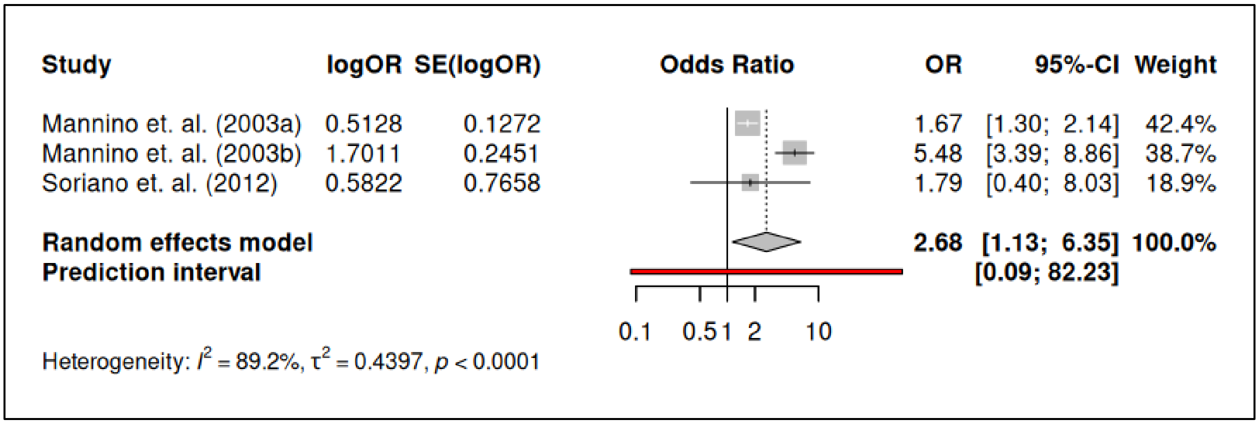

Figure 10.1.25: Pooled univariable odds ratio of underweight (<18.5 kg/m^2) BMI (vs normal 18.5-24.9 kg/m^2 BMI) for GOLD-RSP

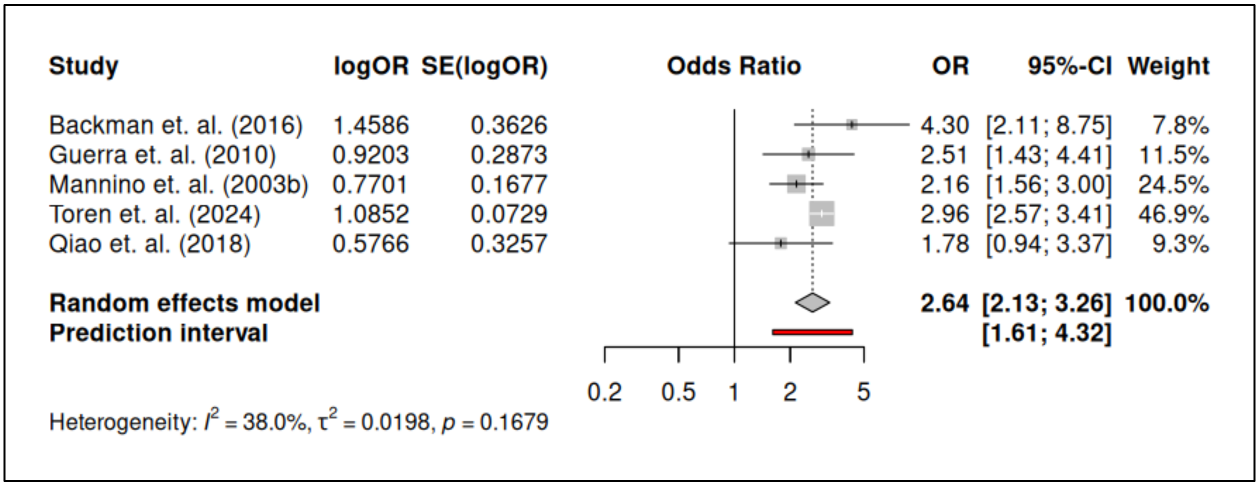

Figure 10.1.26: Pooled univariable odds ratio of history of diabetes for GOLD-RSP

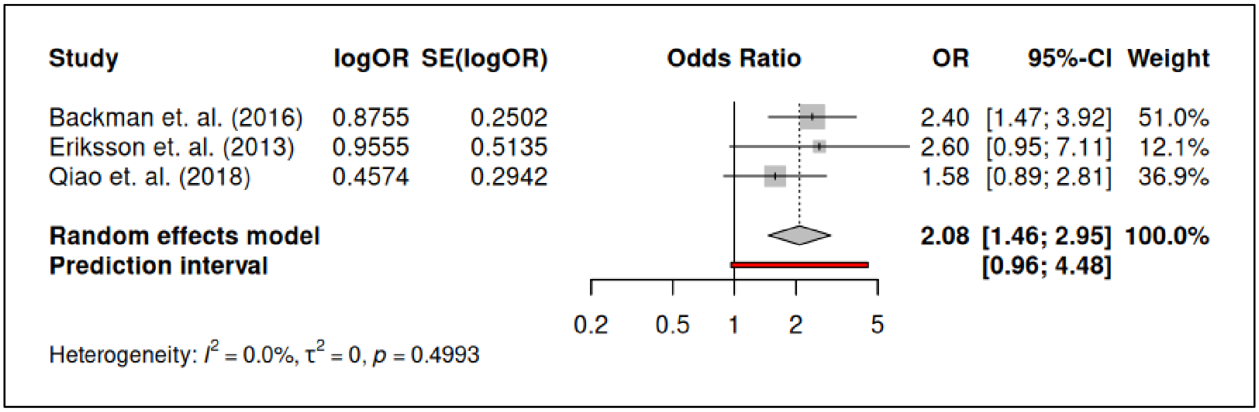

Figure 10.1.27: Pooled univariable odds ratio of history of hypertension for GOLD-RSP

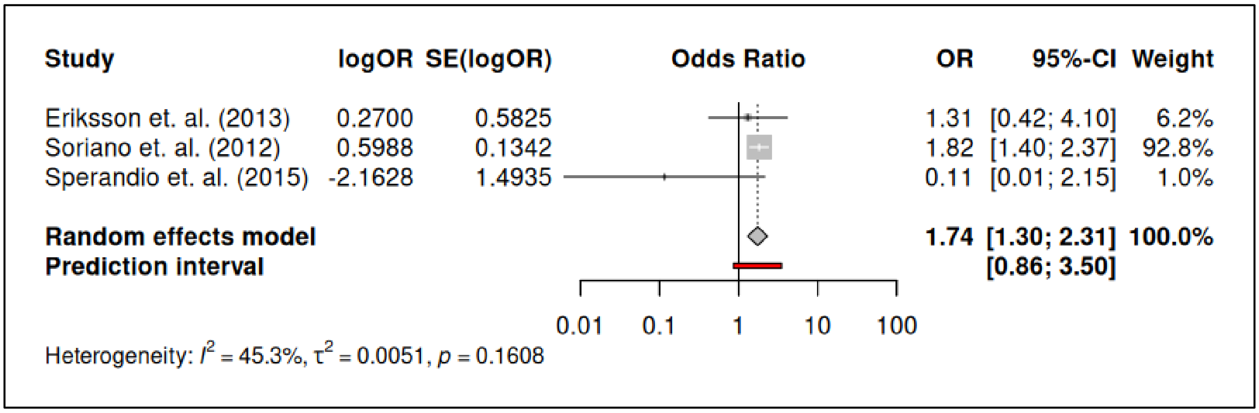

Figure 10.1.28: Pooled multivariable odds ratio of males (vs females) for GOLD-RSP

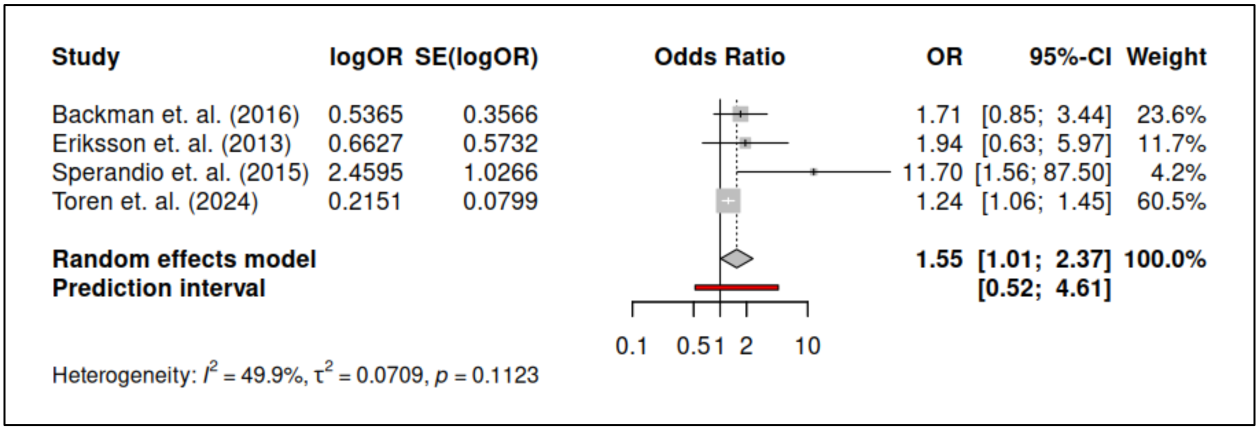

Figure 10.1.29: Pooled multivariable odds ratio of current smokers (vs non-smokers) for GOLD-RSP

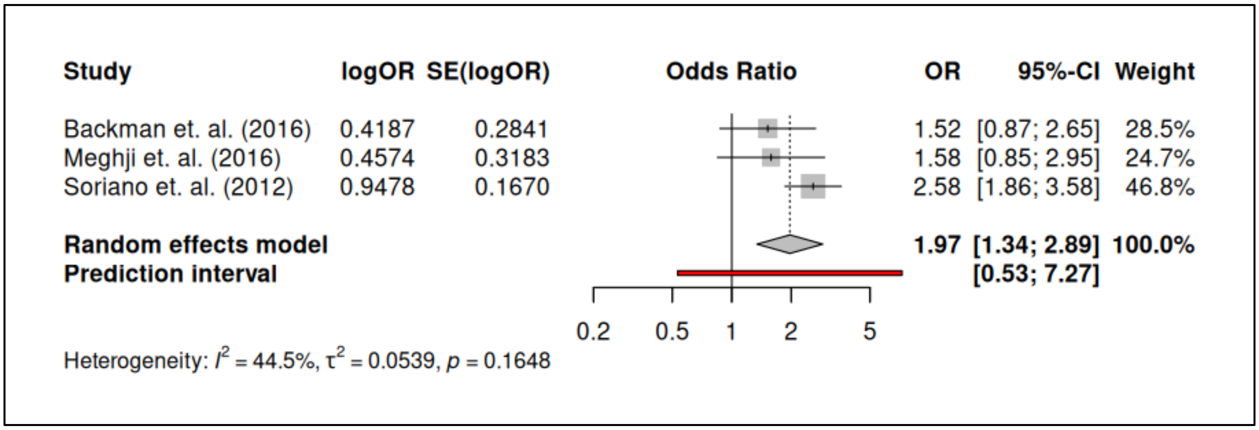

Figure 10.1.30: Pooled multivariable odds ratio of obese (≥30 kg/m²) BMI (vs normal 18.5-24.9 kg/m² BMI) for GOLD-RSP

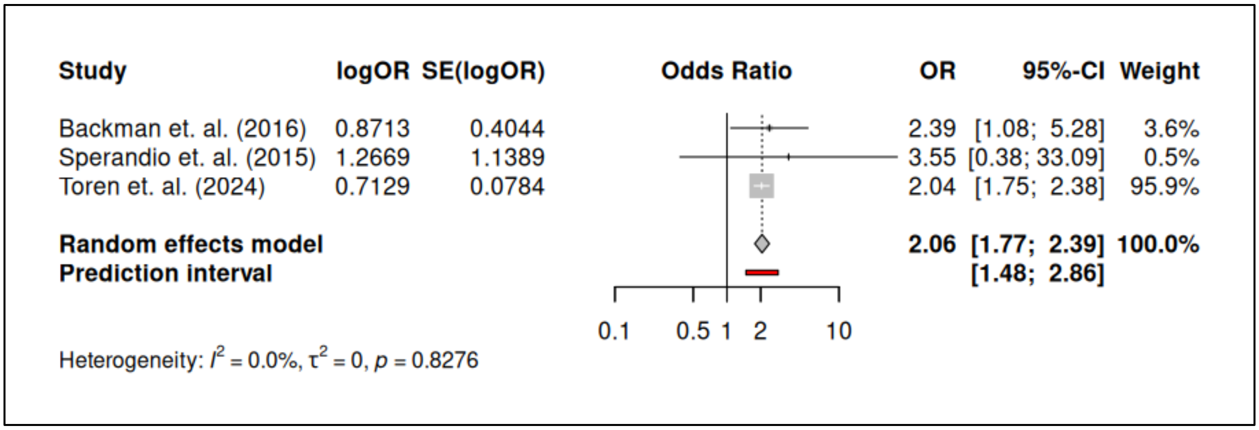

Figure 10.1.31: Pooled multivariable odds ratio of history of diabetes for GOLD-RSP

10.2 LLN studies (supplementary analysis)

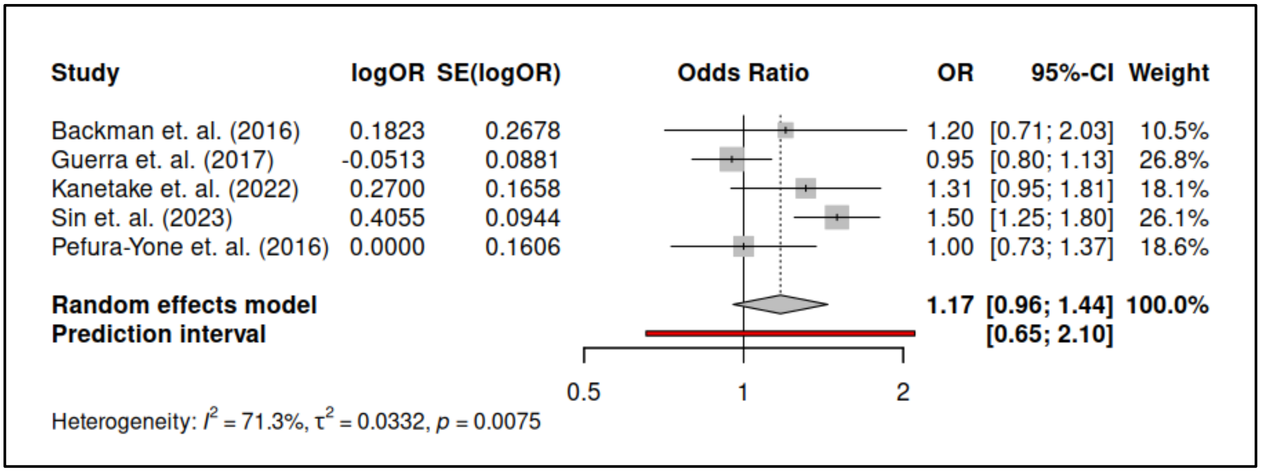

Figure 10.2.1: Pooled univariable odds ratio of males (vs females) for combined LLN-PRISm and LLN-RSP

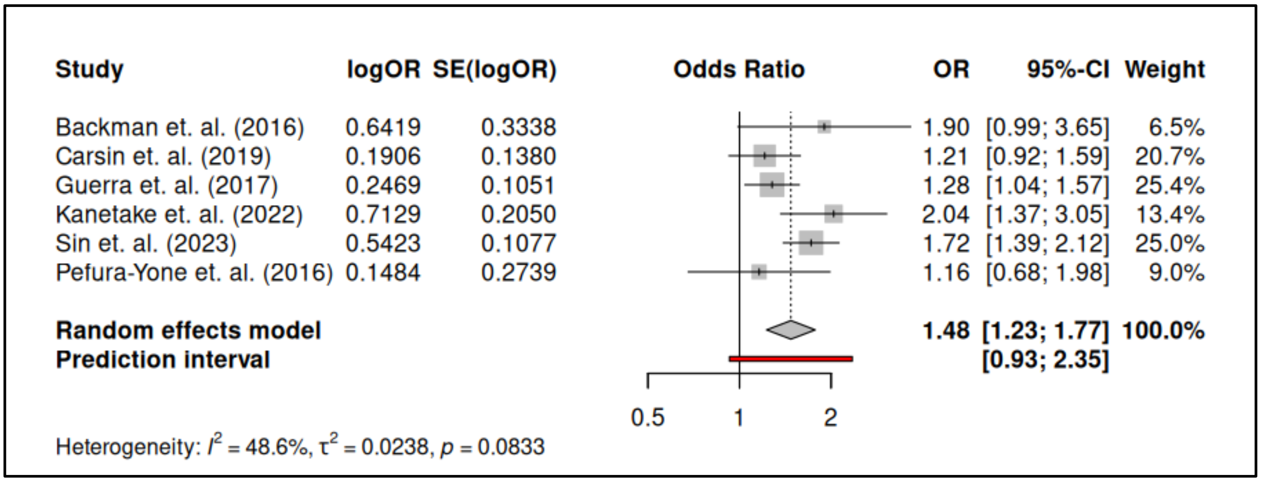

Figure 10.2.2: Pooled univariable odds ratio of current smokers (vs non-smokers) for combined LLN-PRISm and LLN-RSP

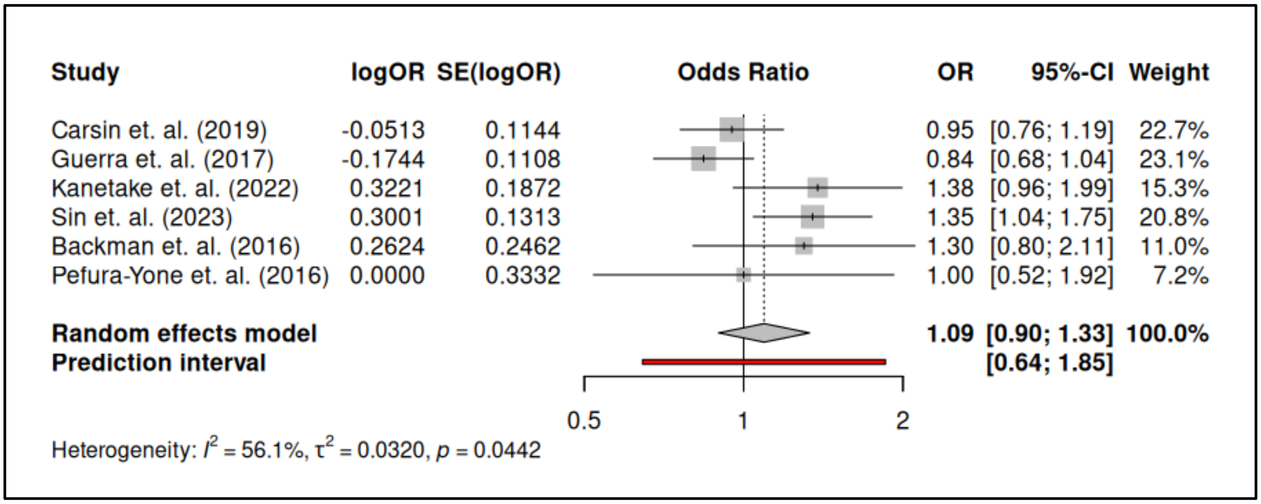

Figure 10.2.3: Pooled univariable odds ratio of former smokers (vs non-smokers) for combined LLN-PRISm and LLN-RSP

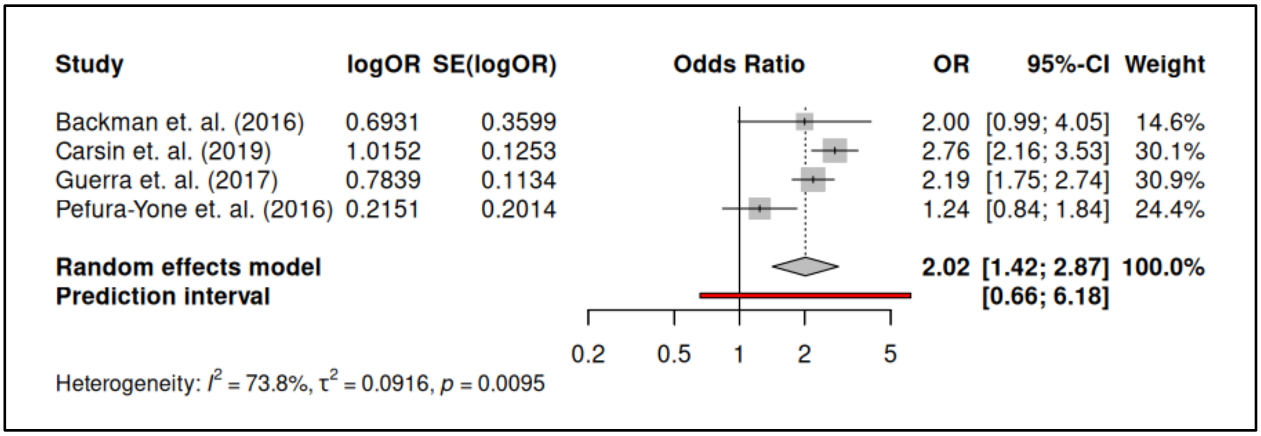

Figure 10.2.4: Pooled univariable odds ratio of obese BMI (≥30 kg/m^2) (vs normal 18.5-24.9 kg/m^2 BMI) for combined LLN-PRISm and LLN-RSP

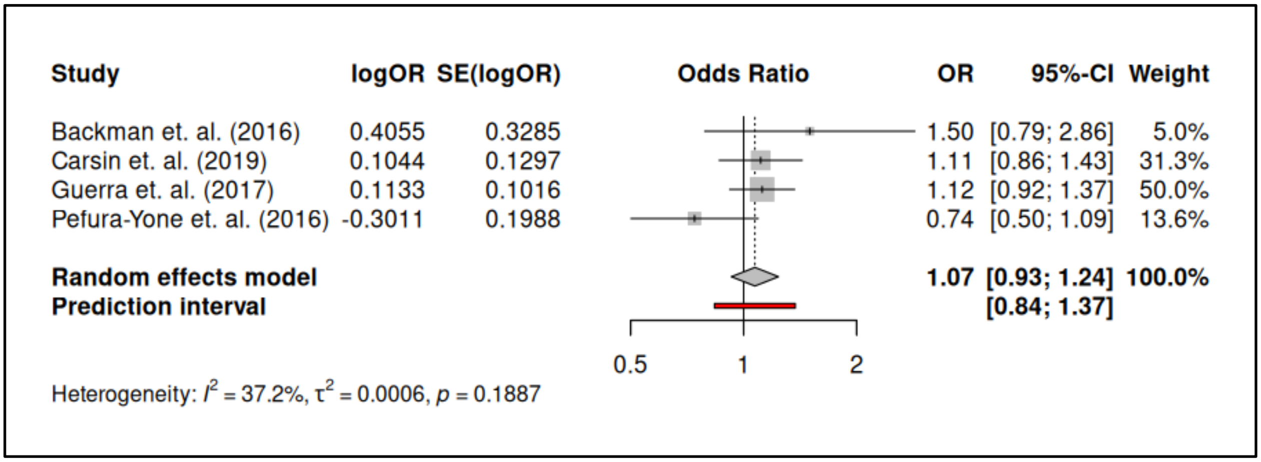

Figure 10.2.5: Pooled univariable odds ratio of overweight (25-29.9 kg/m^2) BMI (vs normal 18.5-24.9 kg/m^2 BMI) for combined LLN-PRISm and LLN-RSP

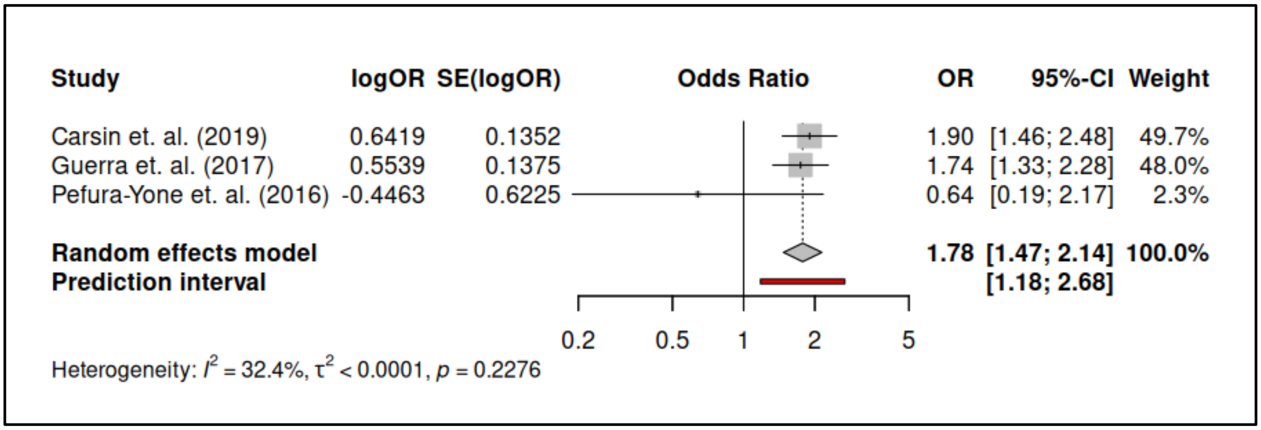

Figure 10.2.6: Pooled univariable odds ratio of history of asthma for combined LLN-PRISm and LLN-RSP

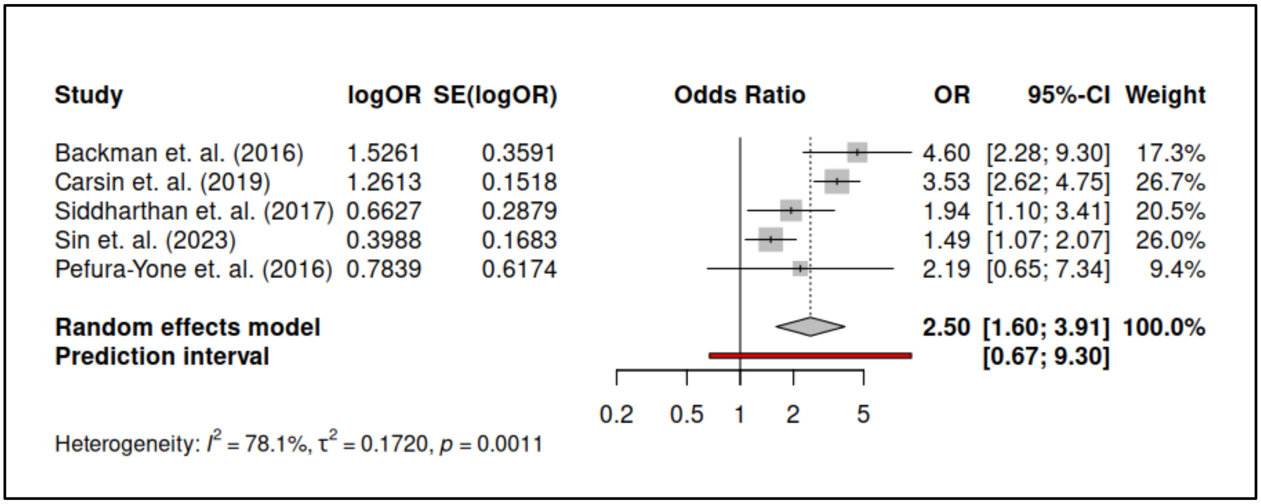

Figure 10.2.7: Pooled univariable odds ratio of history of diabetes for combined LLN-PRISm and LLN-RSP

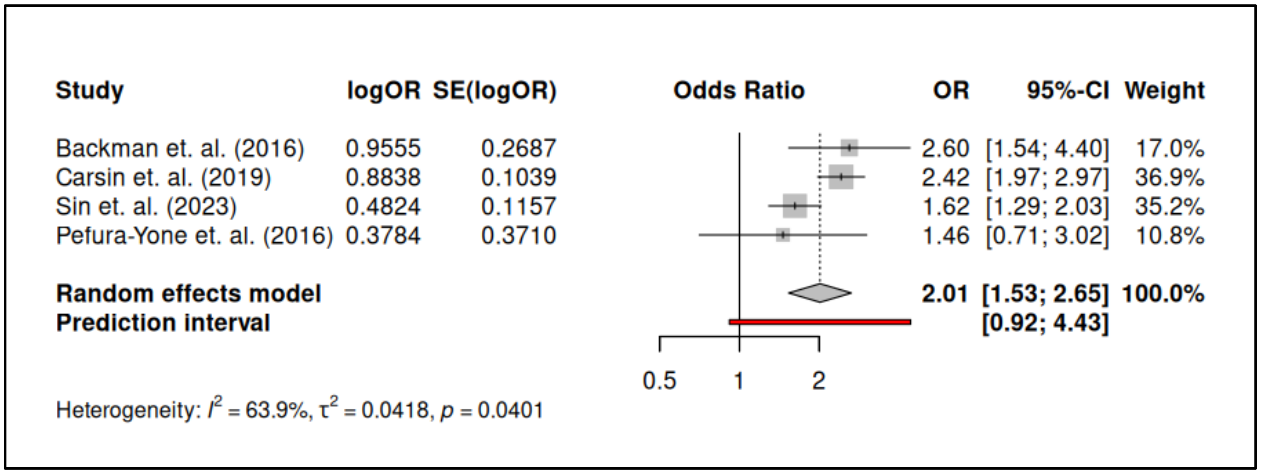

Figure 10.2.8: Pooled univariable odds ratio of history of hypertension for combined LLN-PRISm and LLN-RSP

Supplement S11:Sensitivity analysis - Pooled prevalences of GOLD-PRISm, GOLD-RSP, and combined GOLD-PRISm and GOLD-RSP in subgroups (QA score $\geq$7)

Table 11.1: Sensitivity analysis - consolidated overall prevalences from different modelling approaches (QA score $\geq$7)

Modelling approach	GOLD-PRISm			GOLD-RSP			Combined GOLD-PRISm + GOLD-RSP		
	I^2 (%)	Prevalence (%, 95% CI)	95% PI	I^2 (%)	Prevalence (%, 95% CI)	95% PI	I^2 (%)	Prevalence (%, 95% CI)	95% PI
MLMA – WHO geographical regions	**Level 2:** 95.1<br>**Level 3:** 4.8<br>**Total:** 99.9	10.90 (7.98-14.71)	3.35-30.14	**Level 2:** 74.7<br>**Level 3:** 25.0<br>**Total:** 99.8	10.65 (7.00-15.88)	2.69-33.97	**Level 2:** 86.6<br>**Level 3:** 13.3<br>**Total:** 99.9	11.44 (8.61-15.06)	3.41-32.08
MLMA – World Bank income-levels	**Level 2:** 52.9<br>**Level 3:** 46.9<br>**Total:** 99.9	12.09 (6.93-20.27)	2.98-38.13	**Level 2:** 56.8<br>**Level 3:** 42.9<br>**Total:** 99.7	13.07 (7.10-22.85)	2.82-43.79	**Level 2:** 63.5<br>**Level 3:** 36.4<br>**Total:** 99.9	13.90 (9.08-20.71)	3.84-39.52
Random-effects model	99.9	11.13 (8.4-14.62)	3.11-32.83	99.6	9.94 (7.46-13.12)	2.50-32.25	99.9	11.34 (9.22-13.87)	3.34-32.16

Note: CI – confidence interval, GOLD – Global Initiative for Obstructive Lung Disease, MLMA – multi-level meta-analysis, PI – prediction interval, PRISm – preserved ratio impaired spirometry, and RSP – restrictive spirometric pattern.

Table 11.2: Sensitivity analysis - consolidated overall prevalences from different modelling approaches (Outlier bias)

Modelling approach	GOLD-PRISm			GOLD-RSP			Combined GOLD-PRISm + GOLD-RSP		
	I^2 (%)	Prevalence (%, 95% CI)	95% PI	I^2 (%)	Prevalence (%, 95% CI)	95% PI	I^2 (%)	Prevalence (%, 95% CI)	95% PI
MLMA – WHO geographical regions	**Level 2:** 99.04<br>**Level 3:** 0<br>**Total:** 99.0	10.57 (9.37-11.91)	7.26-15.13	**Level 2:** 93.5<br>**Level 3:** 0<br>**Total:** 93.5	10.62 (9.66-11.66)	7.85-14.23	**Level 2:** 96.7<br>**Level 3:** 0<br>**Total:** 96.7	11.25 (10.48-12.08)	8.42-14.89

Note: CI – confidence interval, GOLD – Global Initiative for Obstructive Lung Disease, MLMA – multi-level meta-analysis, PI – prediction interval, PRISm – preserved ratio impaired spirometry, and RSP – restrictive spirometric pattern.

Outliers were defined as prevalence values lower than the 1st quartile and more than the 3rd quartile.

Table 11.3: Sensitivity analysis - pooled subgroup prevalences of GOLD-PRISm, GOLD-RSP, and combined GOLD-PRISm and GOLD-RSP (QA score ≥7)

Sub-group	GOLD-PRISm				GOLD-RSP				Combined GOLD-PRISm + GOLD-RSP			
	k	I^2 (%)	Prevalence (%, 95% CI)	95% PI	k	I^2 (%)	Prevalence (%, 95% CI)	95% PI	k	I^2 (%)	Prevalence (%, 95% CI)	95% PI
**Prevalence by gender (random-effects model)**												
**Male**	15[a]	99.8	10.17 (7.28-14.04)	2.24-35.84	12[a]	99.1	7.92 (6.03-10.33)	2.64-21.44	26[a]	99.7	9.43 (7.55-11.7)	2.74-27.75
**Female**	14	99.8	10.91 (8.10-14.53)	2.96-32.98	11	99.1	8.12 (6.01-10.87)	2.49-23.37	25	99.7	9.59 (7.72-11.86)	2.93-27.16
**Prevalence by smoking habit (random-effects model)**												
**Non-smoker**	13	99.8	11.44 (8.38-15.41)	3.01-34.95	7	98.2	7.69 (5.61-10.45)	2.55-20.92	20	99.8	9.95 (7.79-12.62)	2.93-28.78
**Ex-smoker**	12	99.3	10.82 (7.47-15.42)	2.30-38.48	7	98.6	7.30 (4.77-11.03)	1.66-26.89	19	99.2	9.38 (7.02-12.41)	2.29-31.35
**Current smoker**	14	99.5	12.76 (9.28-17.30)	3.12-39.88	8	93.4	7.62 (6.19-9.35)	3.75-14.88	22	99.4	10.52 (8.23-13.36)	2.94-31.37
**Prevalence by WHO geographical region (MLMA model)**												
AFRO	-		-	-	2		14.14 (8.53-22.55)	6.29-28.80*	2		13.28 (9.24-18.73)	7.65-22.06*
WPRO	8		11.58 (9.29-14.35)	8.35-15.85	4		11.67 (7.24-18.26)	5.24-23.99	12		12.49 (9.22-16.72)	7.46-20.18
AMRO	2	99.9	10.65 (8.45-13.35)	7.61-14.73	7	99.8	9.28 (5.85-14.41)	4.17-19.39	6	99.9	10.9 (7.83-14.97)	6.38-18.01
EURO	3		10.44 (8.3-13.06)	7.47-14.42*	5		8.33 (5.15-13.20)	3.68-17.74	7		9.43 (6.78-12.98)	5.50-15.71
Multiple regions	2		10.95 (8.69-13.72)	7.83-15.12*	-		-	-	2		11.45 (7.92-16.28)	6.54-19.28*
**Prevalence by World Bank income-level (MLMA model)**												
LIC	-		-		2		17.56 (9.19-30.94)	5.84-42.22*	2		17.47 (10.41-27.84)	7.26-36.40*
UMIC	3	99.9	18.28 (10.32-30.29)	6.62-41.38*	2	99.6	14.41 (7.41-26.15)	4.68-36.62*	5	99.8	17.33 (10.9-26.44)	7.44-35.35
HIC	9		9.88 (5.52-17.06)	3.42-25.33	14		8.67 (4.57-15.82)	2.78-23.91	20		9.64 (6.1-14.9)	5.05-27.95
Mixed income groups	3		9.6 (5.18-17.09)	3.26-25.09*	-		-	-			12.56 (7.31-20.73)	4.02-21.37

Note: AFRO – WHO African Region, AMRO – WHO Region of the Americas, CI – confidence interval, EURO – WHO European Region, GOLD – Global Initiative for Obstructive Lung Disease, HIC – high-income country, LIC – low-income country, MLMA – multi-level meta-analysis, PI – prediction interval, PRISm – preserved ratio impaired spirometry, RSP – restrictive spirometric pattern, and UMIC – upper-middle income country, WHO – World Health Organization and WPRO – WHO Western Pacific Region.

[a] Cestelli et. al (2025) recruited only men in their study. Both GOLD-PRISm and GOLD-RSP outcomes were reported for males. This study, however, was removed from overall prevalence calculations as it did not represent the general population.

*Prediction interval estimates may be imprecise if fewer studies are included in the meta-analysis. Caution is advised when interpreting this estimate.

Supplement S12: Supplementary analysis - Results of overall LLN-PRISm, LLN-RSP, combined LLN-PRISm and RSP, sub-group meta-analyses and meta-regression

MLMA modelling was not performed for LLN-based studies due to lack of studies reporting LLN-PRISm prevalence, and poor data distribution among World Bank income-level groups. Instead, standard random–effects modelling was performed for k≥3 studies.

Table 12.1: Pooled prevalence of LLN-PRISm, LLN-RSP, and combined LLN-PRISm and LLN-RSP

Sub-group	LLN-PRISm				LLN-RSP				Combined LLN-PRISm + LLN-RSP			
	k	I^2 (%)	Prevalence (%, 95% CI)	95% PI	k	I^2 (%)	Prevalence (%, 95% CI)	95% PI	k	I^2 (%)	Prevalence (%, 95% CI)	95% PI
**Overall prevalence (random-effects model)**												
**PRISm**	4	99.9	7.63 (2.69-19.80)	0.15-81.45	-	-	-	-	-	-	-	-
**RSP**	-	-	-	-	12	99.3	6.76 (4.91-9.25)	1.82-22.16	-	-	-	-
**Combined**	-	-	-	-	-	-	-	-	15	99.8	7.55 (5.54-10.22)	1.89-25.77
**Prevalence by gender (random-effects model)**												
**Male**	3[a]	98.2	7.49 (4.57-12.03)	0.82-44.27*	6[a]	99.5	7.89 (4.81-12.68)	1.38-34.46	8[a]	99.3	8.16 (5.59-11.76)	2.04-27.5
**Female**	2	-	-	-	5	98.1	7.30 (4.15-12.53)	1.00-38.11	7	97.4	7.23 (4.82-10.7)	1.71-25.83
**Prevalence by smoking habit (random-effects model)**												
**Non-smoker**	2	-	-	-	3	99.1	7.91 (3.13-18.6)	0.12-86.15*	5	98.3	7.52 (4.43-12.47)	1.16-35.93
**Ex-smoker**	3	78.4	9.01 (6.99-11.53)	3.14-23.24*	3	94.5	7.11 (2.64-17.74)	0.09-87.02*	6	96.2	7.90 (5.05-12.14)	1.69-30.01
**Current smoker**	3	94.1	11.84 (8.18-16.84)	2.29-43.44*	3	94.5	9.11 (3.81-20.24)	0.18-84.76*	6	97.1	10.35 (6.75-15.55)	2.32-35.93
**Prevalence by WHO geographical region (random-effects model)**												
**AFRO**	-	-	-	-	2	-	-	-	2	-	-	-
**WPRO**	2	-	-	-	1	-	-	-	3	94.8	8.17 (6.22-10.67)	2.43-24.08*
**AMRO**	1	-	-	-	5	96.9	6.83 (5.16-9.00)	2.56-17.00	6	99.8	8.42 (5.22-13.29)	1.54-35.05

**EURO**	1	-	-	-	4	99.1	4.54 (2.44-8.28)	0.47-32.57	4[b]	99.2	4.49 (2.37-8.35)	0.43-33.9
**Multiple regions**	-	-	-	-	1	-	-	-	1	-	-	-
**Prevalence by World Bank income-level (random-effects model)**												
LIC	-	-	-	-	1	-	-	-	1	-	-	-
LMIC	-	-	-	-	1	-	-	-	1	-	-	-
UMIC	-	-	-	-	2	-	-	-	2	-	-	-
HIC	4	99.9	7.63 (2.69-19.80)	0.15-81.45	7	99.5	5.98 (3.94-8.96)	1.35-22.83	10	99.8	7.32 (4.87-10.87)	1.49-29.26
Mixed income groups	-	-	-	-	1	-	-	-	1	-	-	-

Note: AFRO – WHO African Region, AMRO – WHO Region of the Americas, CI – confidence interval, EURO – WHO European Region, HIC – high-income country, LIC – low-income country, LMIC – low-middle income country, LLN – lower limit of normal, MLMA – multi-level meta-analysis, PI – prediction interval, PRISm – preserved ratio impaired spirometry, RSP – restrictive spirometric pattern, UMIC – upper-middle income country, and WPRO – WHO Western Pacific Region. Meta-analysis was only conducted if 3 or more studies were included.

[a] Cestelli et. al (2025) recruited only men in their study. Both LLN-PRISm and LLN-RSP outcomes were reported for males. This study, however, was removed from overall prevalence calculations as it did not represent the general population.

[b] Toren et. al. (2024) reported both LLN-PRISm and LLN-RSP outcomes - only LLN-PRISm outcome was included in the combined analysis.

*Prediction interval estimates may be imprecise if fewer studies are included in the meta-analysis. Caution is advised when interpreting this estimate.

.

Table 12.2: Pooled estimates for risk factors for combined LLN-PRISm and LLN-RSP

Risk factor	Reference group	Combined LLN-PRISm and LLN-RSP[a]			
		k	I^2 (%)	Pooled OR (95% CI)	95% PI
**Univariable odds ratios**					
Gender - male	female	5	71.3	1.17 (0.96-1.44)	0.65-2.10
Current smoking	Never smoking	6	48.6	**1.48 (1.23-1.77)**	0.93-2.35
Former smoking	Never smoking	6	56.1	1.09 (0.90-1.33)	0.64-1.85
Obesity (BMI $\geq$30 kg/m^2)	18.5-24.9 kg/m^2	4	73.8	**2.02 (1.42-2.87)**	0.66-6.18
Overweight (25-29.9 kg/m^2)	18.5-24.9 kg/m^2	4	37.2	1.07 (0.93-1.24)	0.84-1.37
History of asthma	No history of asthma	3	32.4	**1.78 (1.47-2.14)**	1.18-2.68
History of diabetes	No history of diabetes	5	78.1	**2.50 (1.60-3.91)**	0.67-9.30
History of hypertension	No history of hypertension	4	63.9	**2.01 (1.53-2.65)**	0.92-4.43

Note: CI – confidence interval, BMI – body mass index, LLN – lower limit of normal, OR – odds ratio, PI – prediction interval, PRISm – preserved ratio impaired spirometry, RSP – restrictive spirometric pattern

An estimate is statistically significant if its 95% CI does not contain the null value (1.00).

[a] Estimates were only reported for combined LLN-PRISm and RSP outcome because of insufficient studies reporting LLN-PRISm and LLN-RSP outcomes individually.

Table 12.3: Results of mixed effect meta-regression (LLN)

Variable	Group	Combined LLN-PRISm and LLN-RSP[a]		
		β (95% CI) (logit proportion)	p	R^2 (%)
**Univariable meta-regression**				
**WHO geographical location (ref: WPRO)**	**AFRO**	0.97 (-0.18 to 2.13)	0.10	52.76
	**AMRO**	-0.16 (-1.13 to 0.82)	0.75	
	**EURO**	-0.82 (-1.85 to 0.22)	0.12	
**World Bank income-level (ref: HIC)**	**LIC**	1.31 (-0.16 to 2.78)	0.08	12.50
	**LMIC**	1.32 (-0.68 to 3.32)	0.20	
	**UMIC**	0.08 (-1.39 to 1.55)	0.91	
**Bronchodilator use (ref: No)**		-0.37 (-1.38 to 0.64)	0.48	0.00
**Mean age**		-0.04 (-0.1 to 0.03)	0.29	1.14
**% females in study population**		-0.01 (-0.11 to 0.08)	0.79	0.00
**% current smokers in study population**		-0.04 (-0.1 to 0.01)	0.12	12.82
**Publication year**		-0.08 (-0.21 to 0.05)	0.22	2.67

Note: AFRO – WHO African Region, AMRO – WHO Region of the Americas, CI – confidence interval, EURO – WHO European Region, HIC – high-income country, LIC – low-income country, LMIC – low-middle income country, LLN – lower limit of normal, PRISm – preserved ratio impaired spirometry, RSP – restrictive spirometric pattern, UMIC – upper-middle income country, and WPRO – WHO Western Pacific Region.

[a] Estimates were only reported for combined LLN-PRISm and RSP outcome b ecause of insufficient studies reporting LLN-PRISm and LLN-RSP outcomes individually.

Supplement S13: Forest plots – LLN studies (supplementary analysis)

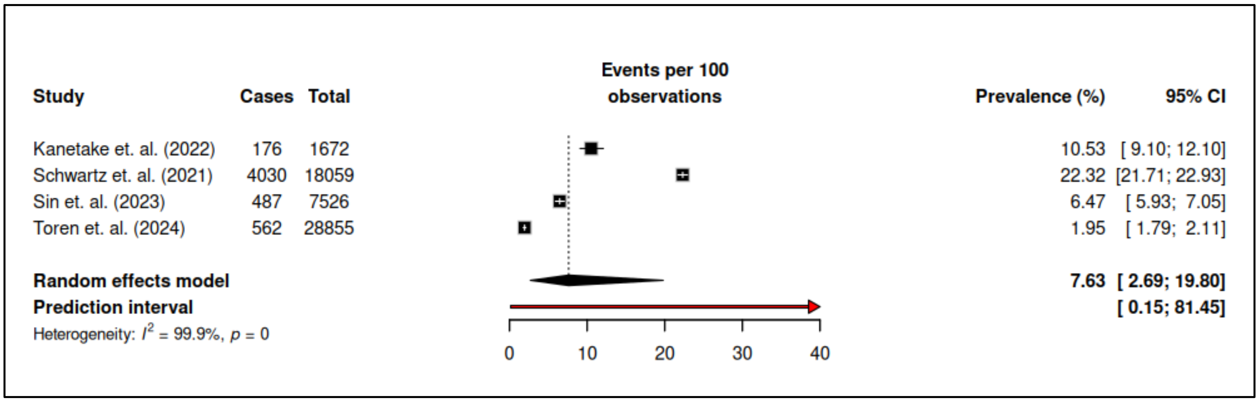

Figure 13.1: Prevalence of LLN-PRISm

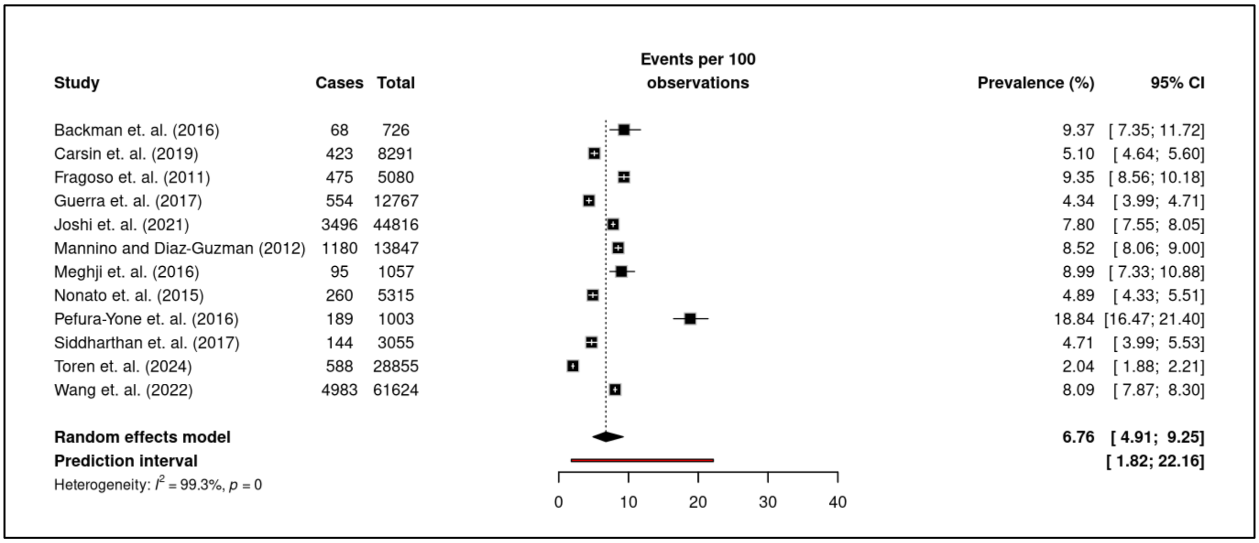

Figure 13.2: Prevalence of LLN-RSP

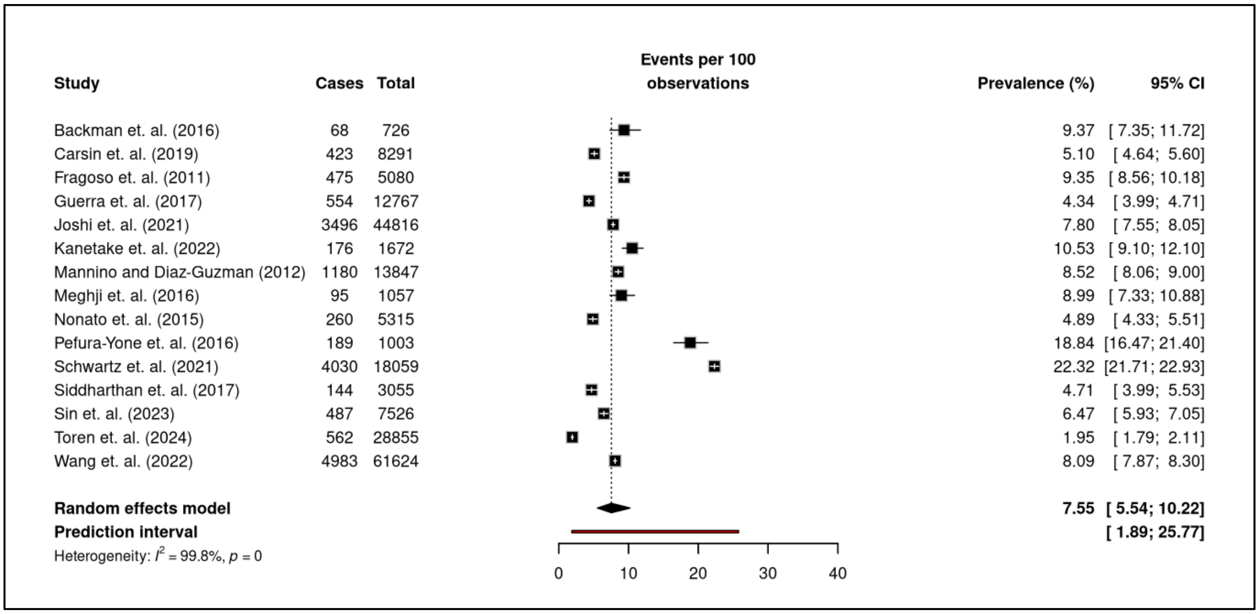

Figure 13.3: Combined prevalence of LLN-PRISm and LLN-RSP

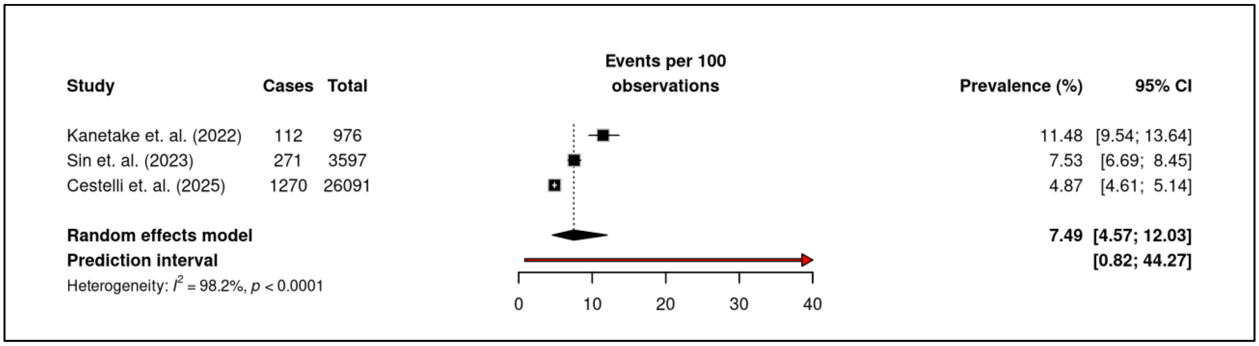

Figure 13.4: Prevalence of LLN-PRISm in males

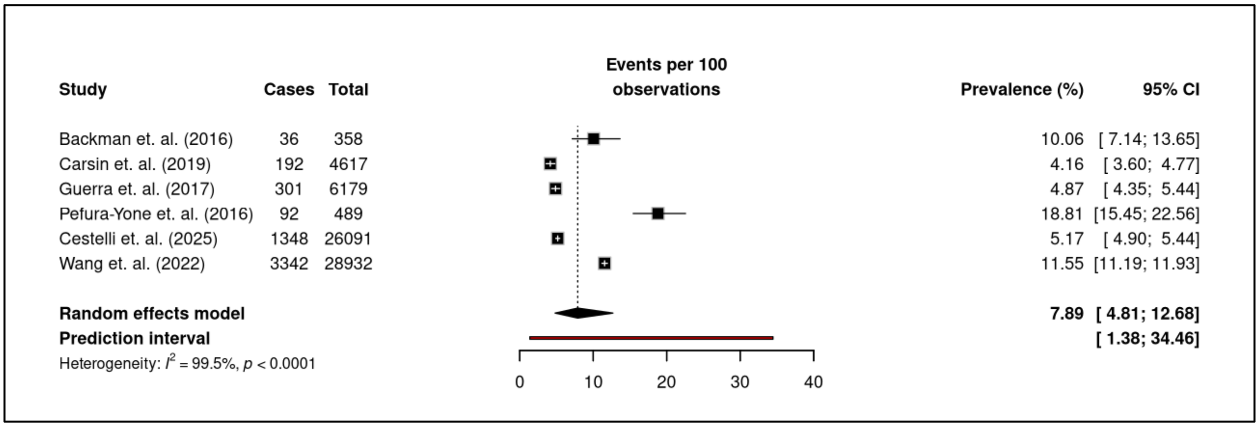

Figure 13.5: Prevalence of LLN-RSP in males

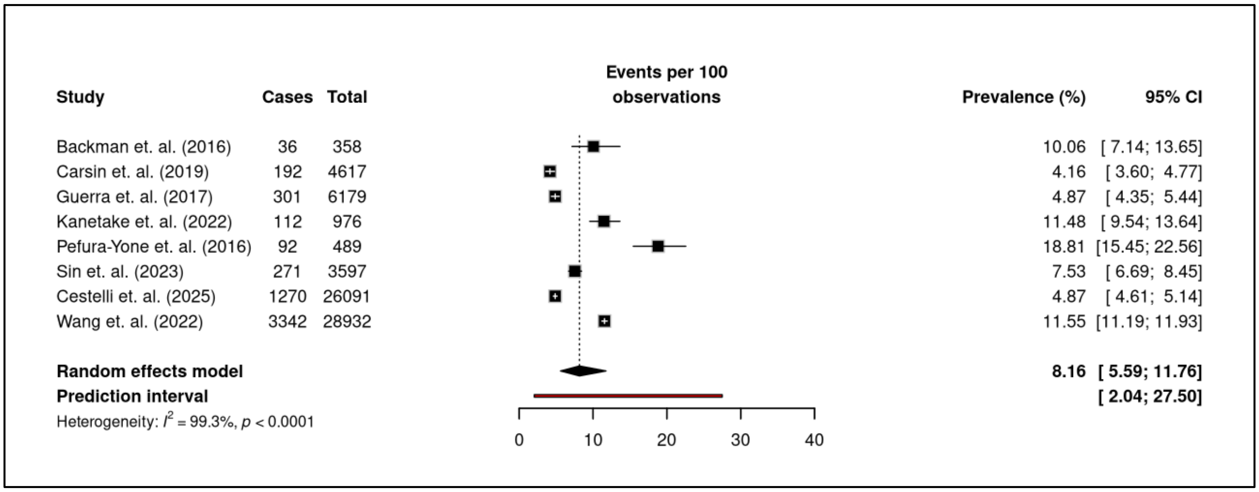

Figure 13.6: Combined prevalence of LLN-PRISm and LLN-RSP in males

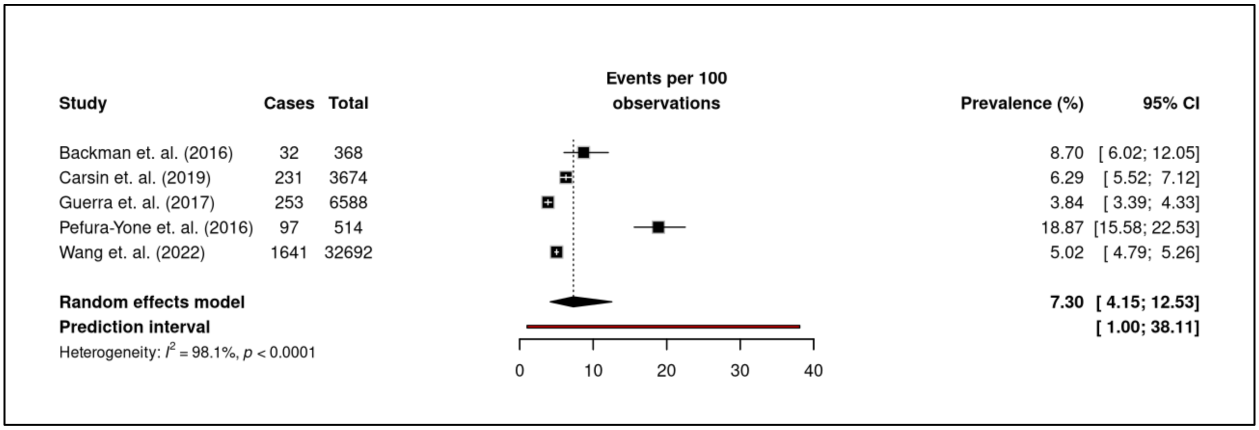

Figure 13.7: Prevalence of LLN-RSP in females

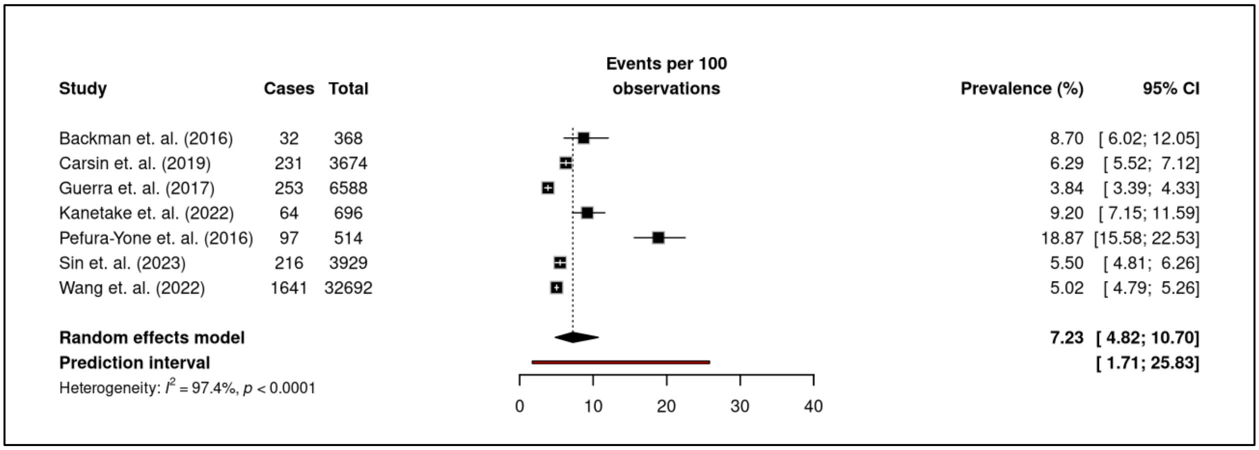

Figure 13.8: Combined prevalence of LLN-PRISm and LLN-RSP in females

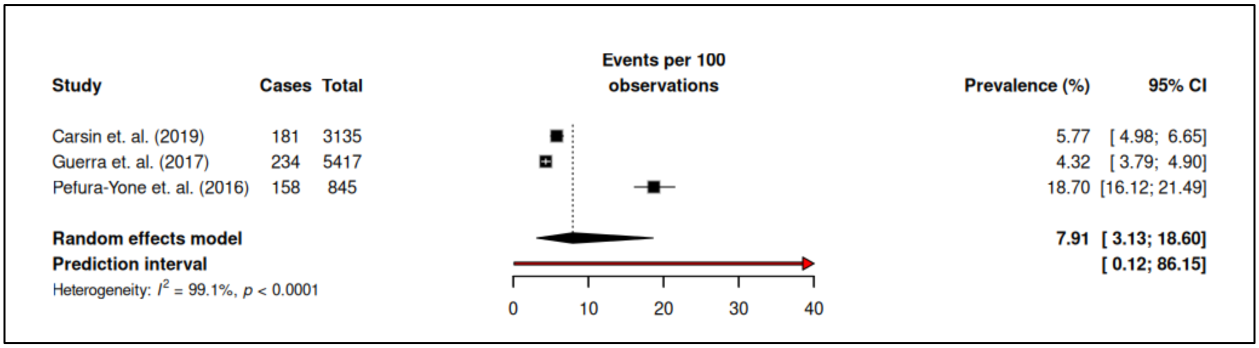

Figure 13.9: Prevalence of LLN-RSP in non-smokers

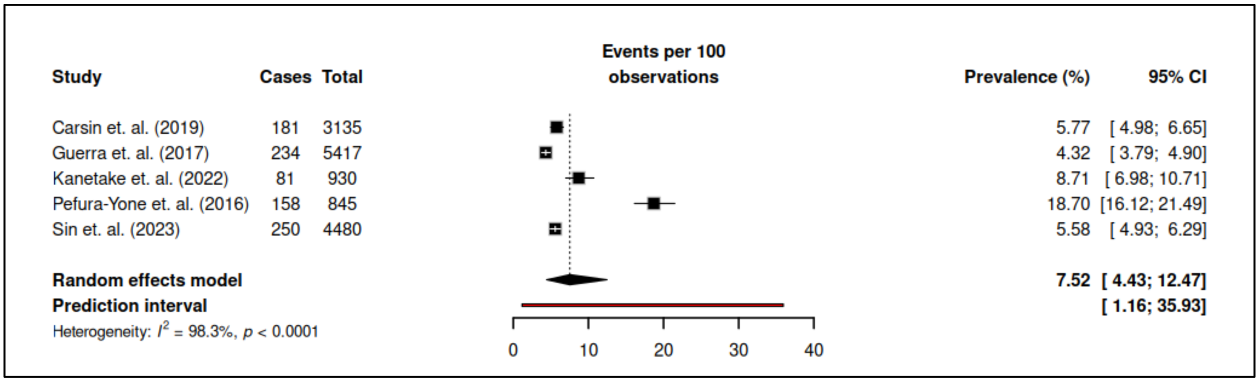

Figure 13.10: Combined prevalence of LLN-PRISm and LLN-RSP in non-smokers

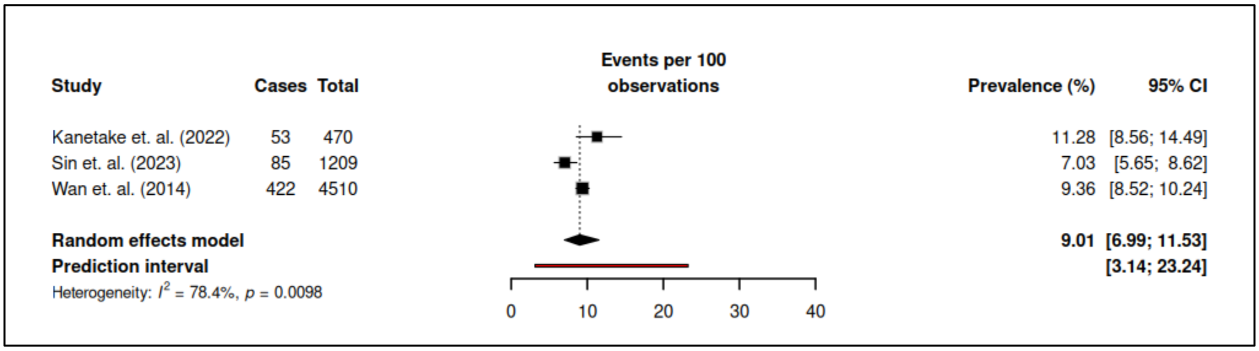

Figure 13.11: Prevalence of LLN-PRISm in ex-smokers

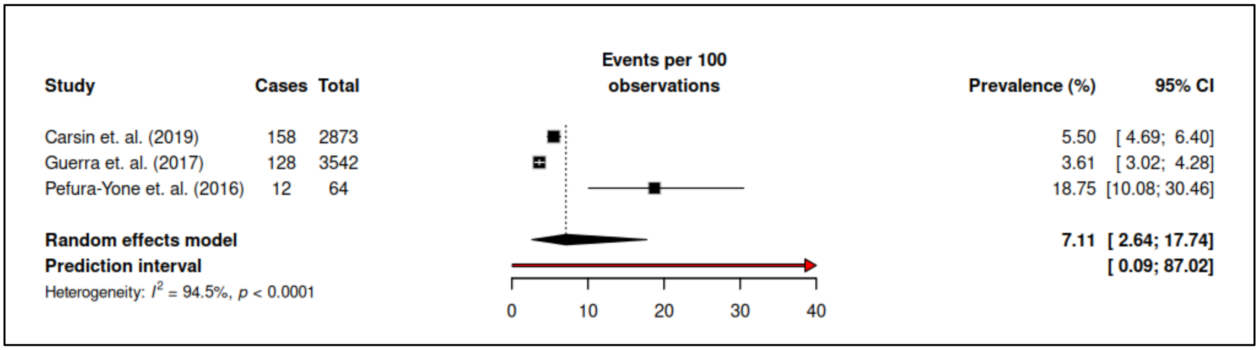

Figure 13.12: Prevalence of LLN-RSP in ex-smokers

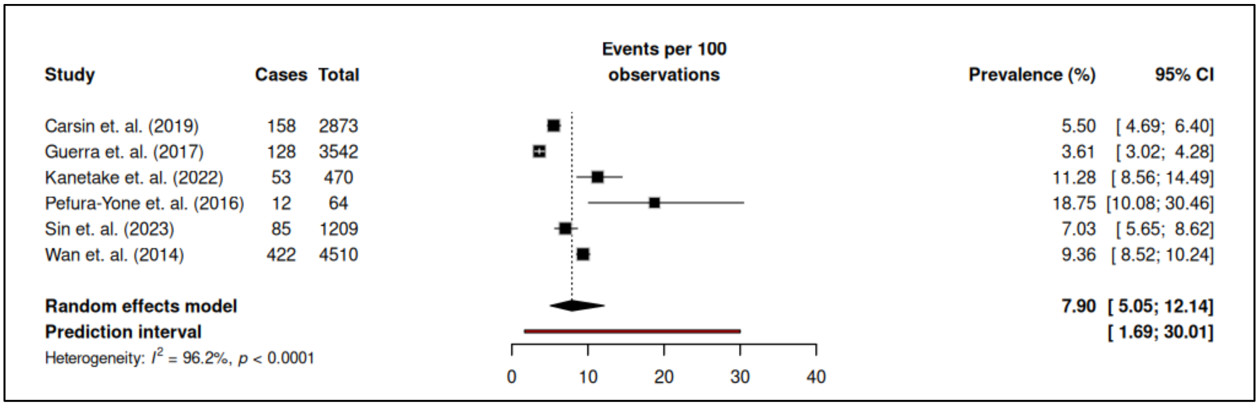

Figure 13.13: Combined prevalence of LLN-PRISm and LLN-RSP in ex-smokers

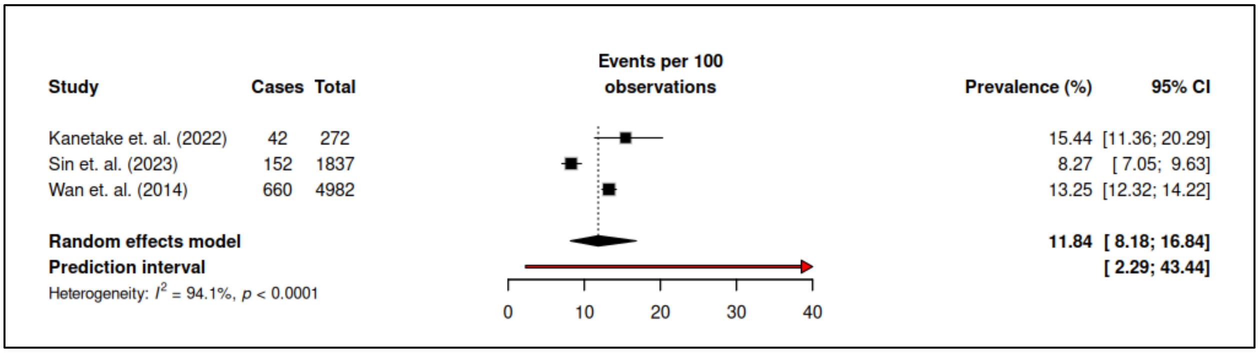

Figure 13.14: Prevalence of LLN-PRISm in current smokers

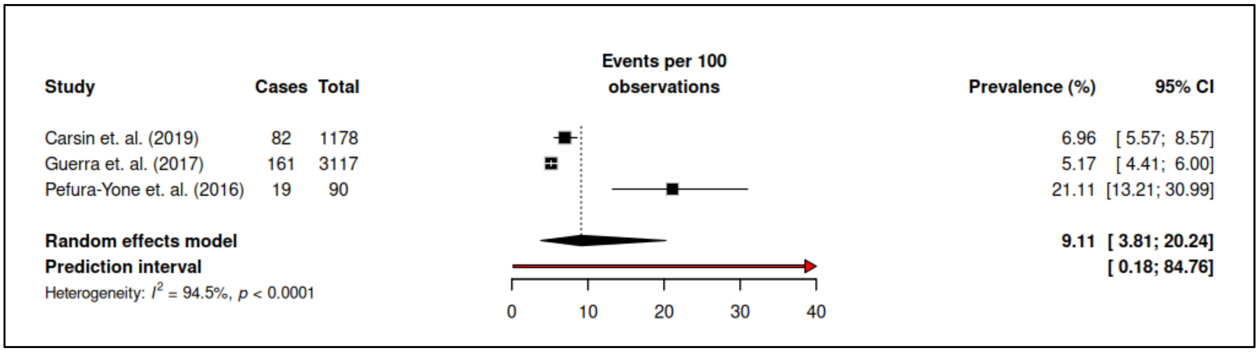

Figure 13.15: Prevalence of LLN-RSP in current smokers

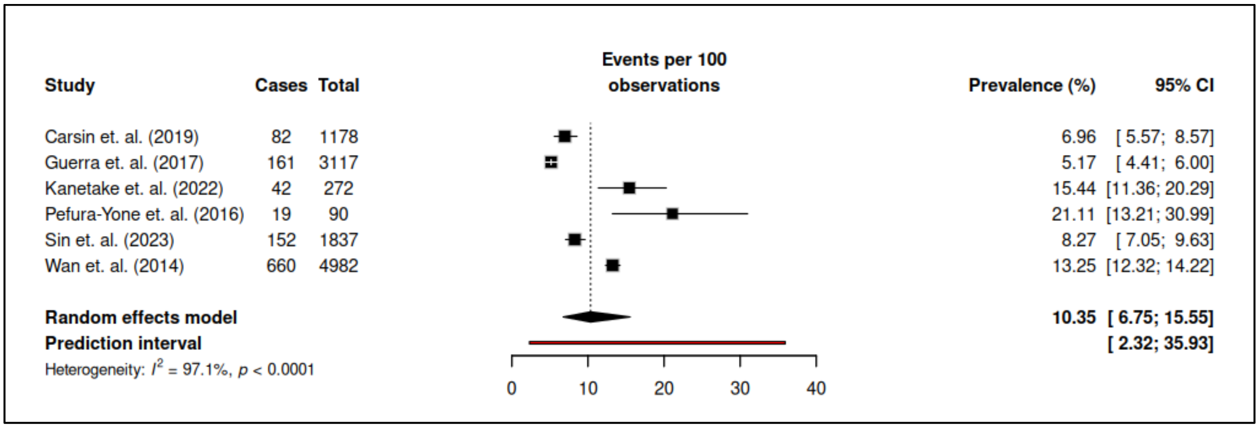

Figure 13.16: Combined prevalence of LLN-PRISm and LLN-RSP in current smokers

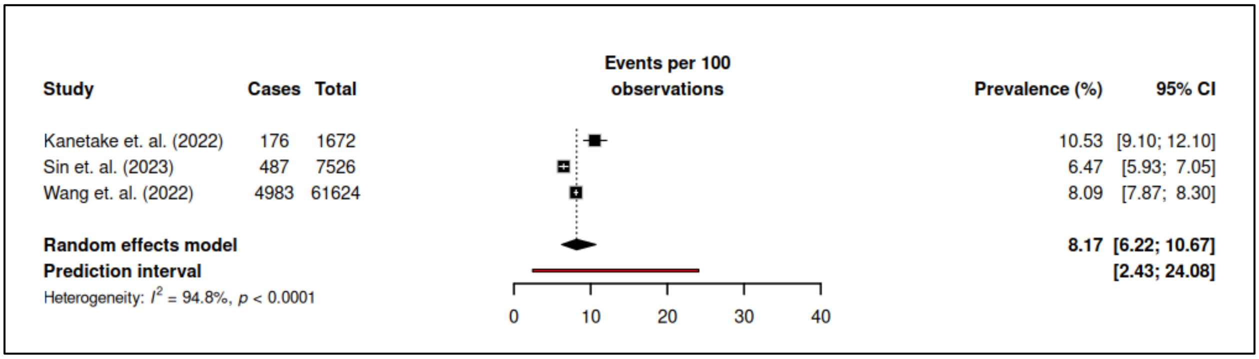

Figure 13.17: Combined prevalence of LLN-PRISm and LLN-RSP in WPRO region

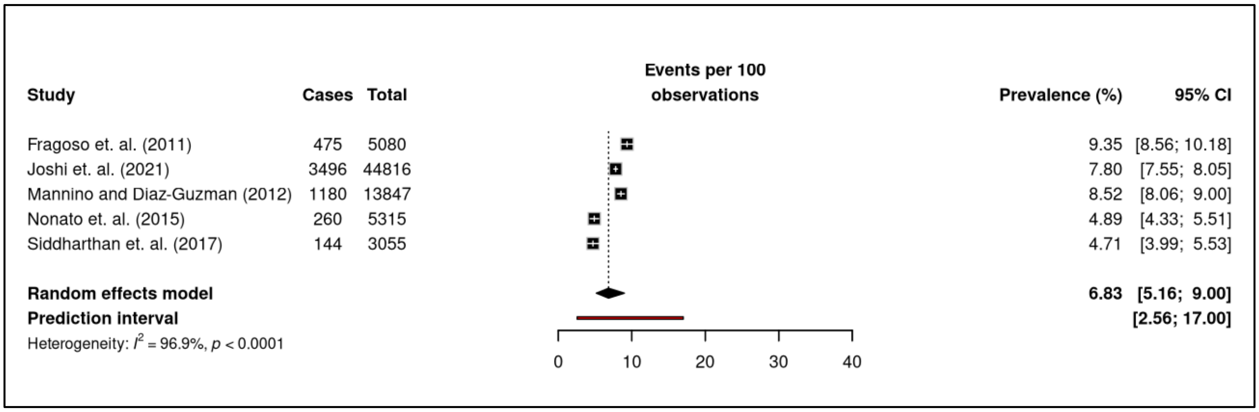

Figure 13.18: Prevalence of LLN-RSP in AMRO region

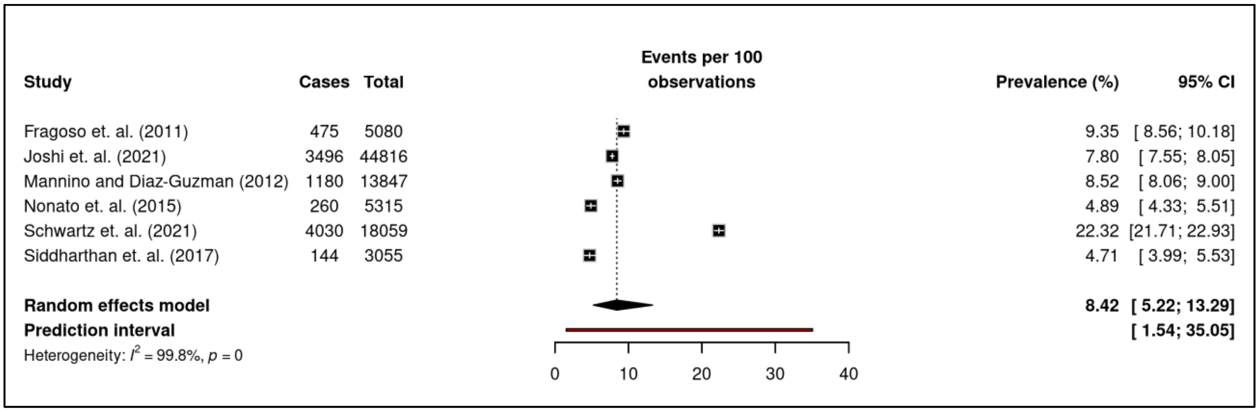

Figure 13.19: Combined prevalence of LLN-PRISm and LLN-RSP in AMRO region

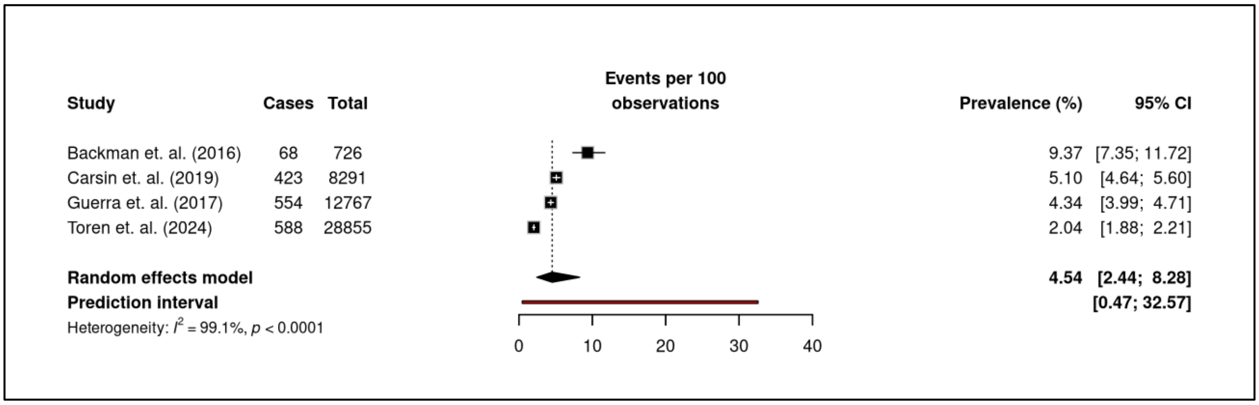

Figure 13.20: Prevalence of LLN-RSP in EURO region

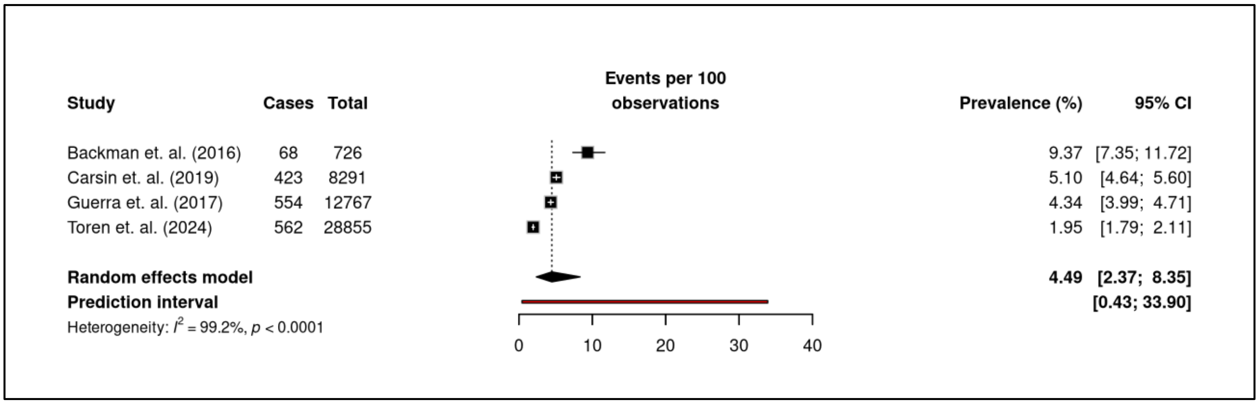

Figure 13.21: Combined prevalence of LLN-PRISm and LLN-RSP in EURO region

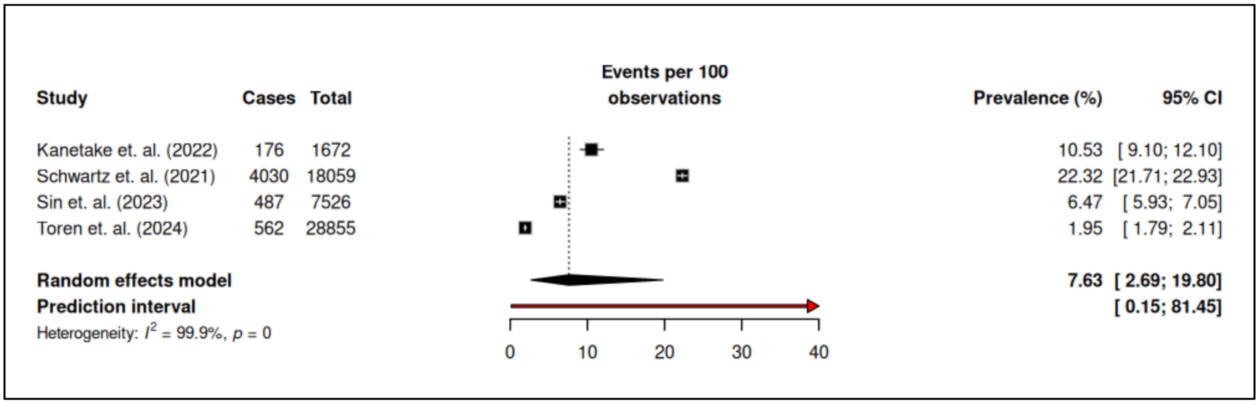

Figure 13.22: Prevalence of LLN-PRISm in high-income countries

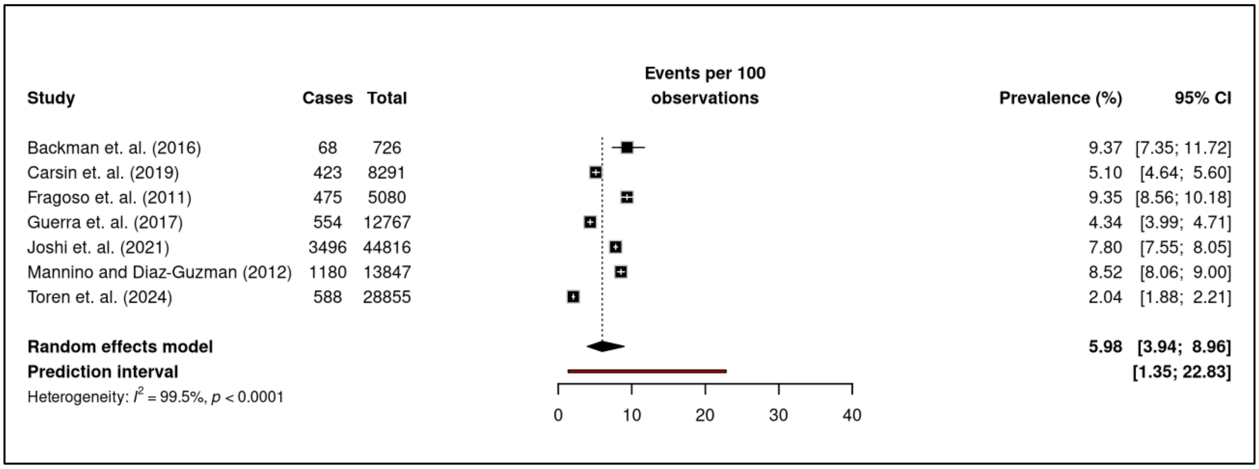

Figure 13.23: Prevalence of LLN-RSP in high-income countries

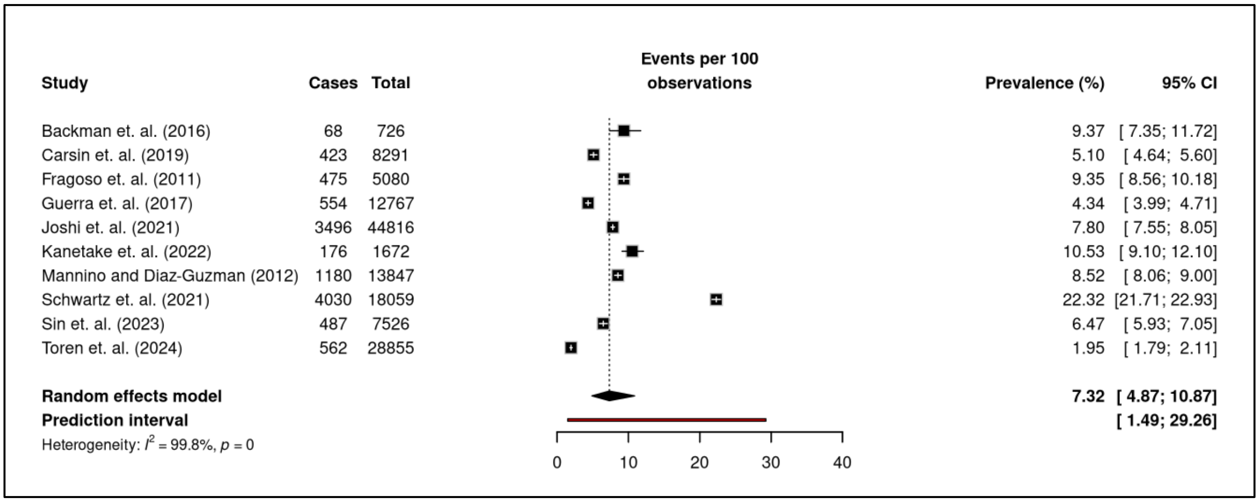

Figure 13.24: Combined prevalence of LLN-PRISm and LLN-RSP in high-income countries

Supplement S14: Tests to investigate publication bias

Publication bias was investigated visually using funnel plots and statistically using Egger's regression tests.[63] For Egger's tests, precision was fitted as a moderator in multi-level models. A p-value <0.05 indicated publication bias. The presence of publication bias was investigated for studies reporting prevalence of GOLD-PRISm, GOLD-RSP, and combined GOLD-PRISm and GOLD-RSP (multi-level models with WHO regions as clustering variables). Funnel plots and outputs from Egger's test are reported below.

14.1 Overall prevalence of GOLD-PRISm

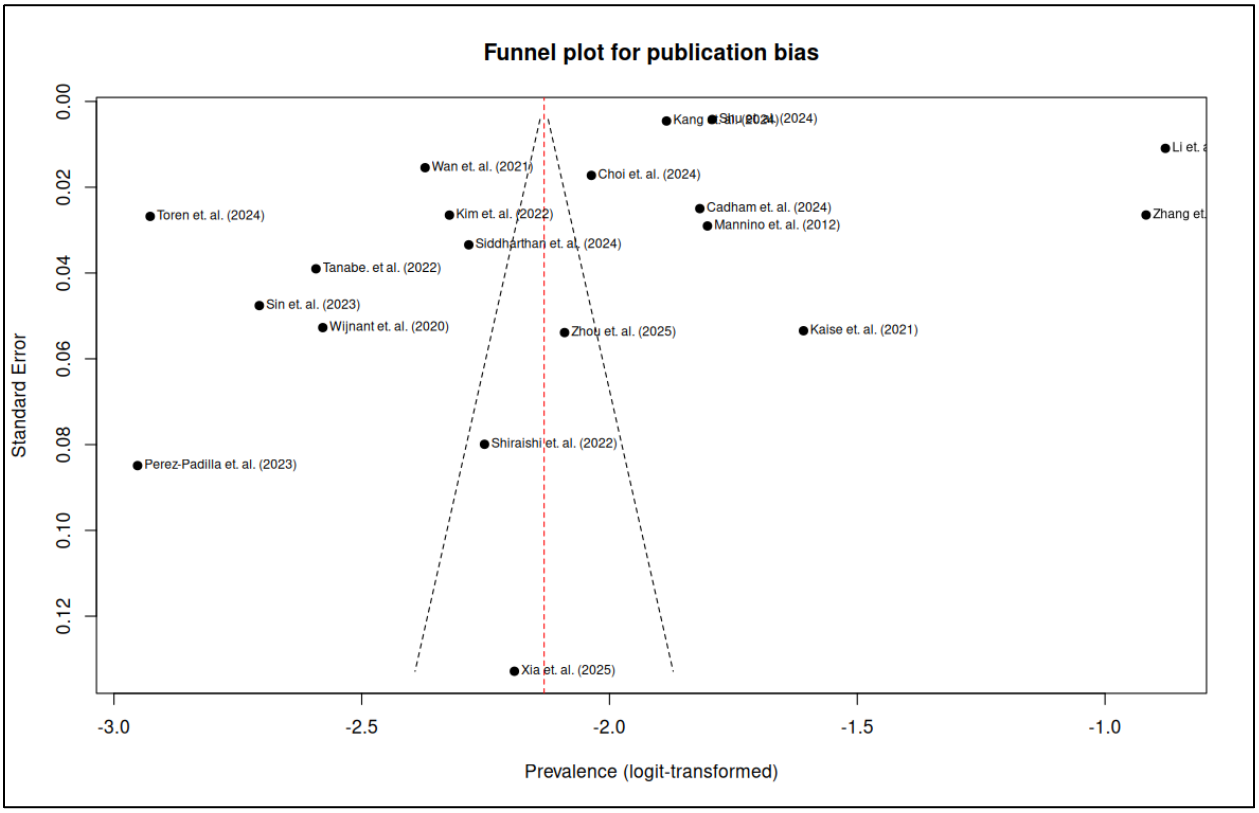

Figure 11.1: Funnel plot of studies reporting prevalence of GOLD-PRISm

Egger's regression test R output:

Model Results:

```
          estimate     se      zval    pval    ci.lb    ci.ub
intrcpt    -2.3138  0.1968  -11.7592  <.0001  -2.6995  -1.9282  ***
precision   0.0029  0.0020    1.4879  0.1368  -0.0009   0.0068
```

14.2 Overall prevalence of GOLD-RSP

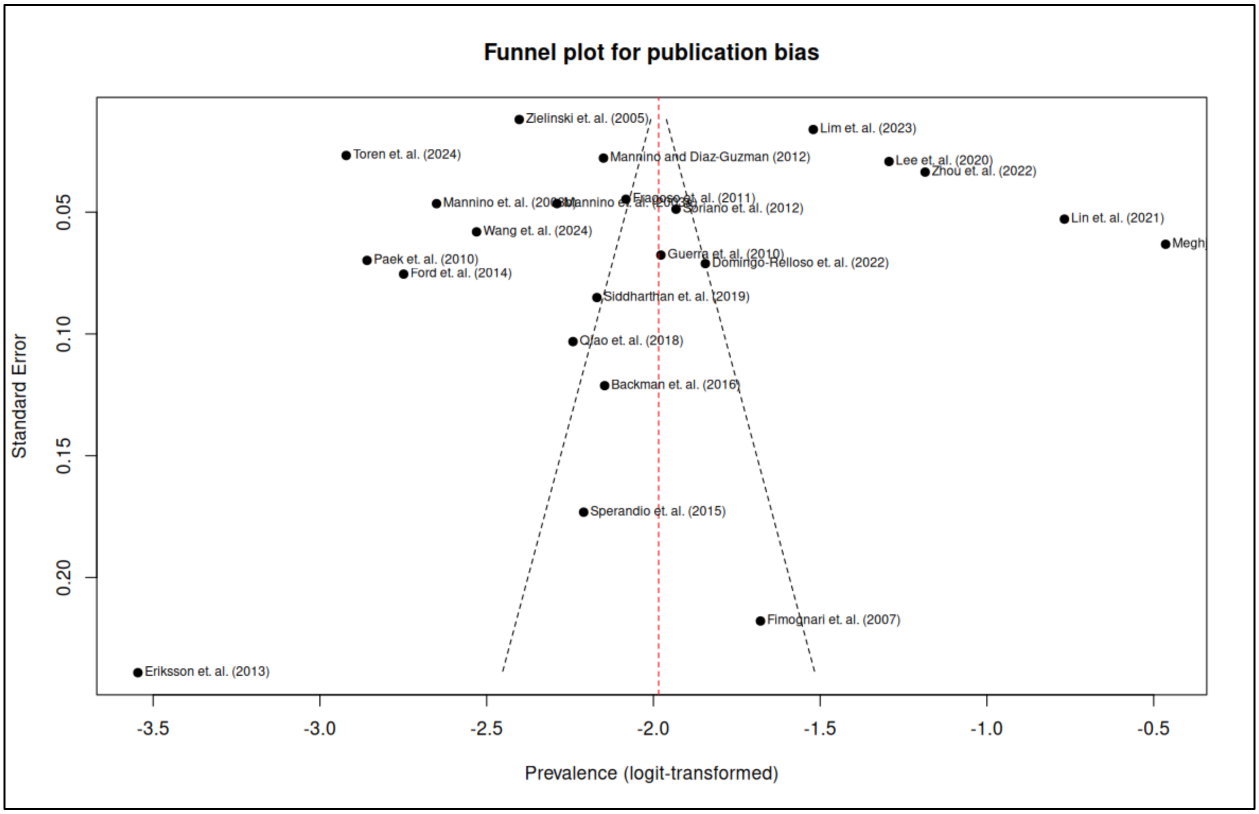

Figure 11.2: Funnel plot of studies reporting prevalence of GOLD-RSP

Egger's test output (from R):

Model Results:

	estimate	se	zval	pval	ci.lb	ci.ub	
intrcpt	-2.0537	0.2957	-6.9463	<.0001	-2.6332	-1.4742	***
precision	0.0030	0.0074	0.3992	0.6898	-0.0116	0.0176	

14.3 Overall prevalence of combined GOLD-PRISm and GOLD-RSP

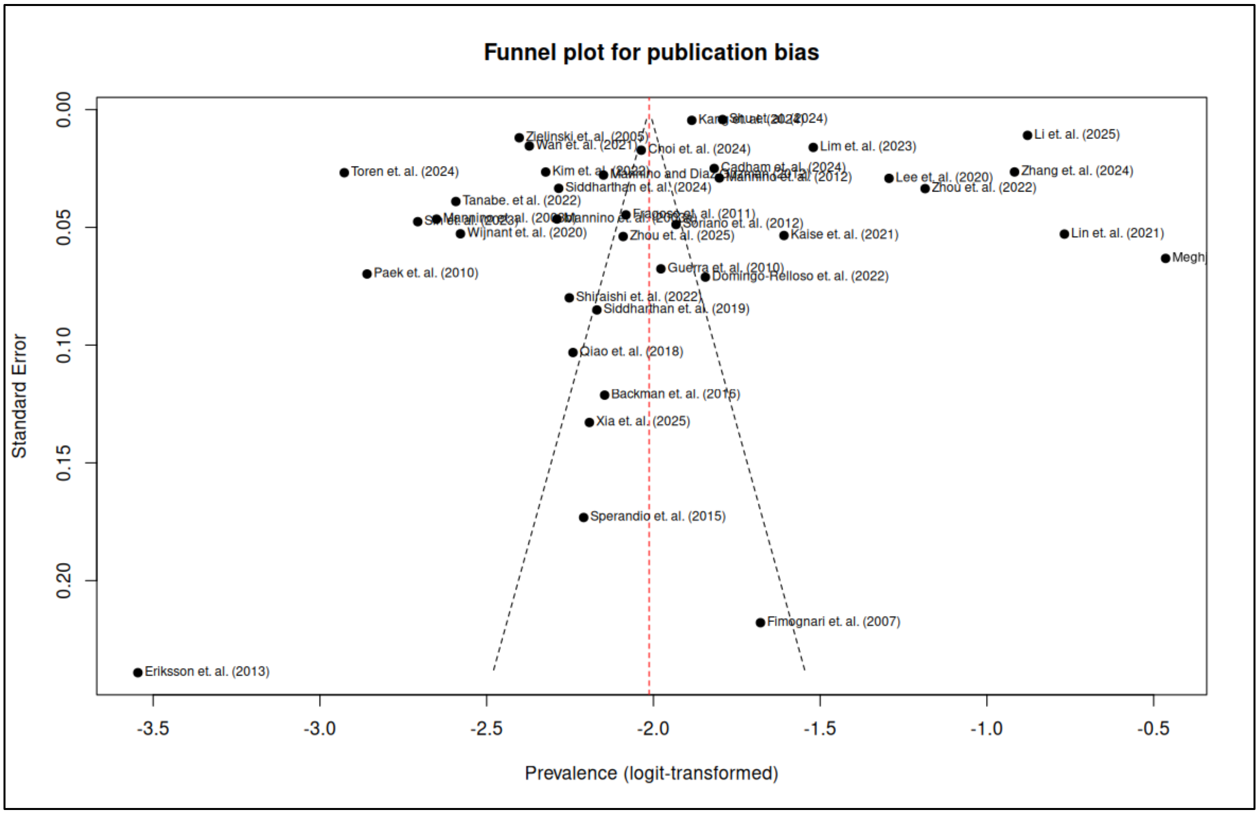

Figure 11.3: Funnel plot of studies reporting prevalence of GOLD-PRISm and GOLD-RSP

Egger's test output (from R):

	estimate	se	zval	pval	ci.lb	ci.ub	
intrcpt	-2.0832	0.1697	-12.2779	<.0001	-2.4157	-1.7506	***
precision	0.0020	0.0020	0.9950	0.3197	-0.0019	0.0058	

References

1. Backman H, Eriksson B, Hedman L, Stridsman C, Jansson S-A, Sovijärvi A, et al. Restrictive spirometric pattern in the general adult population: Methods of defining the condition and consequences on prevalence. Respiratory Medicine. 2016;120:116-23.
2. Cadham CJ, Oh H, Han MK, Mannino D, Cook S, Meza R, et al. The prevalence and mortality risks of PRISm and COPD in the United States from NHANES 2007–2012. Respiratory Research. 2024;25(1):208.
3. Carsin A-E, Fuertes E, Schaffner E, Jarvis D, Antó JM, Heinrich J, et al. Restrictive spirometry pattern is associated with low physical activity levels. A population based international study. Respiratory Medicine. 2019;146:116-23.
4. Cestelli L, Johannessen A, Gulsvik A, Stavem K, Nielsen R. Risk Factors, Morbidity, and Mortality in Association With Preserved Ratio Impaired Spirometry and Restrictive Spirometric Pattern: Clinical Relevance of Preserved Ratio Impaired Spirometry and Restrictive Spirometric Pattern. CHEST. 2025;167(2):548-60.
5. Choi H, Oak C-H, Jung M-H, Jang T-W, Nam S-J, Yoon T. Trend of prevalence and characteristics of preserved ratio impaired spirometry (PRISm): Nationwide population-based survey between 2010 and 2019. PLOS ONE. 2024;19(7):e0307302.
6. Domingo-Relloso A, Riffo-Campos AL, Powers M, Tellez-Plaza M, Haack K, Brown RH, et al. An epigenome-wide study of DNA methylation profiles and lung function among American Indians in the Strong Heart Study. Clinical Epigenetics. 2022;14(1):75.
7. Eriksson B, Lindberg A, Müllerova H, Rönmark E, Lundbäck B. Association of heart diseases with COPD and restrictive lung function – Results from a population survey. Respiratory Medicine. 2013;107(1):98-106.
8. Fimognari FL, Pasqualetti P, Moro L, Franco A, Piccirillo G, Pastorelli R, et al. The Association Between Metabolic Syndrome and Restrictive Ventilatory Dysfunction in Older Persons. The Journals of Gerontology: Series A. 2007;62(7):760-5.
9. Ford ES, Cunningham TJ, Mercado CI. Lung function and metabolic syndrome: Findings of National Health and Nutrition Examination Survey 2007–2010. Journal of Diabetes. 2014;6(6):603-13.
10. Vaz Fragoso CA, Gill TM, McAvay G, Yaggi HK, Van Ness PH, Concato J. Respiratory Impairment and Mortality in Older Persons: A Novel Spirometric Approach. Journal of Investigative Medicine. 2011;59(7):1089-95.
11. Guerra S, Carsin A-E, Keidel D, Sunyer J, Leynaert B, Janson C, et al. Health-related quality of life and risk factors associated with spirometric restriction. European Respiratory Journal. 2017;49(5):1602096.
12. Guerra S, Sherrill DL, Venker C, Ceccato CM, Halonen M, Martinez FD. Morbidity and mortality associated with the restrictive spirometric pattern: a longitudinal study. Thorax. 2010;65(6):499-504.
13. Higbee DH, Granell R, Davey Smith G, Dodd JW. Prevalence, risk factors, and clinical implications of preserved ratio impaired spirometry: a UK Biobank cohort analysis. Lancet Respir Med. 2022;10(2):149-57.
14. Joshi D, Duong M, Kirkland S, Raina P. Impact of electronic cigarette ever use on lung function in adults aged 45–85: a cross-sectional analysis from the Canadian Longitudinal Study on Aging. BMJ Open. 2021;11(10):e051519.

15. Kaaks R, Christodoulou E, Motsch E, Katzke V, Wielpütz MO, Kauczor H-U, et al. Lung function impairment in the German Lung Cancer Screening Intervention Study (LUSI): prevalence, symptoms, and associations with lung cancer risk, tumor histology and all-cause mortality. Translational Lung Cancer Research. 2022;11(9):1896-911.
16. Kaise T, Sakihara E, Tamaki K, Miyata H, Hirahara N, Kirichek O, et al. Prevalence and Characteristics of Individuals with Preserved Ratio Impaired Spirometry (PRISm) and/or Impaired Lung Function in Japan: The OCEAN Study. Int J Chron Obstruct Pulmon Dis. 2021;16:2665-75.
17. Rina K, Kazufumii T, Kaechang P, Akihito Y. Prevalence and risk factors for COPD in subjects with preserved ratio impaired spirometry. BMJ Open Respiratory Research. 2022;9(1):e001298.
18. Kang Z, Zhang J, Zhu C, Zhu Y, Jiang H, Tong Q, Dai S-M. Impaired pulmonary function increases the risk of gout: evidence from a large cohort study in the UK Biobank. BMC Medicine. 2024;22(1):606.
19. Kim J, Lee C-H, Lee HY, Kim H. Association between Comorbidities and Preserved Ratio Impaired Spirometry: Using the Korean National Health and Nutrition Examination Survey IV–VI. Respiration. 2021;101(1):25-33.
20. Kwon E, Jin T, You Y-A, Kim B. Joint effect of long-term exposure to ambient air pollution on the prevalence of chronic obstructive pulmonary disease using the Korea National Health and Nutrition Examination Survey 2010–2019. Chemosphere. 2024;358:142137.
21. Lee Y-Y, Tsao Y-C, Yang C-K, Chuang C-H, Yu W, Chen J-C, Li W-C. Association between risk factors of metabolic syndrome with lung function. European Journal of Clinical Nutrition. 2020;74(5):811-7.
22. Li M, Li Y, Chen M, Mylinh D, Cai Q, Wang B, et al. Association between Preserved Ratio Impaired Spirometry with mortality and long-term cardiovascular outcomes in Chinese adults2025.
23. Lim H-S, Kim D-K, Gil H-I, Lee M-Y, Lee H-S, Lee Y-T, et al. Association of Pulmonary Function with Osteosarcopenic Obesity in Older Adults Aged over 50 Years. Nutrients. 2023;15(13):2933.
24. Lin Y-C, Huang T-J, Yeh M-H, Lin M-S, Chen M-Y. Lung function impairment and cardiometabolic risks among rural adults: implication for an aging society. BMC Public Health. 2021;21(1):960.
25. Mannino DM, Buist AS, Petty TL, Enright PL, Redd SC. Lung function and mortality in the United States: data from the First National Health and Nutrition Examination Survey follow up study. Thorax. 2003;58(5):388-93.
26. Mannino DM, Ford ES, Redd SC. Obstructive and restrictive lung disease and functional limitation: data from the Third National Health and Nutrition Examination. Journal of Internal Medicine. 2003;254(6):540-7.
27. Mannino DM, Diaz-Guzman E. Interpreting Lung Function Data Using 80% Predicted and Fixed Thresholds Identifies Patients at Increased Risk of Mortality. CHEST. 2012;141(1):73-80.
28. Mannino DM, McBurnie MA, Tan W, Kocabas A, Anto J, Vollmer WM, Buist AS. Restricted spirometry in the Burden of Lung Disease Study. Int J Tuberc Lung Dis. 2012;16(10):1405-11.

29. Meghji J, Nadeau G, Davis KJ, Wang D, Nyirenda MJ, Gordon SB, Mortimer K. Noncommunicable Lung Disease in Sub-Saharan Africa. A Community-based Cross-Sectional Study of Adults in Urban Malawi. Am J Respir Crit Care Med. 2016;194(1):67-76.
30. Nonato NL, Nascimento OA, Padilla RP, de Oca MM, Tálamo C, Valdivia G, et al. Occurrence of respiratory symptoms in persons with restrictive ventilatory impairment compared with persons with chronic obstructive pulmonary disease:The PLATINO study. Chronic Respiratory Disease. 2015;12(3):264-73.
31. Paek Y-J, Jung K-S, Hwang Y-I, Lee K-S, Lee DR, Lee J-U. Association between low pulmonary function and metabolic risk factors in Korean adults: the Korean National Health and Nutrition Survey. Metabolism - Clinical and Experimental. 2010;59(9):1300-6.
32. Pefura-Yone EW, Balkissou AD, Kengne AP. Determinants of Restrictive Spirometric Pattern in a Sub-Saharan Urban Setting: A Cross-sectional Population-based Study. Open Respir Med J. 2016;10:86-95.
33. Perez-Padilla R, de Oca MM, Thirion-Romero I, Wehrmeister FC, Lopez MV, Valdivia G, et al. Trajectories of Spirometric Patterns, Obstructive and PRISm, in a Population-Based Cohort in Latin America. INTERNATIONAL JOURNAL OF CHRONIC OBSTRUCTIVE PULMONARY DISEASE. 2023;18:1277-85.
34. Qiao Q, Zhang X, He X, Xi S. Epidemiological survey on pulmonary functions of people over 40 years old in Laiyuan County, Hebei Province. Clinical Focus. 2022;37(7):627-30.
35. Schwartz A, Arnold N, Skinner B, Simmering J, Eberlein M, Comellas AP, Fortis S. Preserved Ratio Impaired Spirometry in a Spirometry Database. Respiratory Care. 2020;66(1):58-65.
36. Shiraishi Y, Shimada T, Tanabe N, Terada K, Sakamoto R, Maetani T, et al. The prevalence and physiological impacts of centrilobular and paraseptal emphysema on computed tomography in smokers with preserved ratio impaired spirometry. ERJ Open Research. 2022;8(2):00063-2022.
37. Shu CC, Tsai MK, Lee JH, Su TC, Wen CP. Mortality risk in patients with preserved ratio impaired spirometry: assessing the role of physical activity. QJM: An International Journal of Medicine. 2024;117(6):436-44.
38. Siddharthan T, Grigsby M, Miele CH, Bernabe-Ortiz A, Miranda JJ, Gilman RH, et al. Prevalence and risk factors of restrictive spirometry in a cohort of Peruvian adults. Int J Tuberc Lung Dis. 2017;21(9):1062-8.
39. Siddharthan T, Grigsby M, Morgan B, Kalyesubula R, Wise RA, Kirenga B, Checkley W. Prevalence of chronic respiratory disease in urban and rural Uganda. Bull World Health Organ. 2019;97(5):318-27.
40. Siddharthan T, Grealis K, Robertson NM, Lu M, Liu S, Pollard SL, et al. Assessing the prevalence and impact of preserved ratio impaired spirometry in low-income and middle-income countries: a post-hoc cross-sectional analysis. The Lancet Global Health. 2024;12(9):e1498-e505.
41. Sin S, Lee EJ, Won S, Kim WJ. Longitudinal mortality of preserved ratio impaired spirometry in a middle-aged Asian cohort. BMC Pulmonary Medicine. 2023;23(1):155.
42. Soriano JB, Miravitlles M, García-Río F, Muñoz L, Sánchez G, Sobradillo V, et al. Spirometrically-defined restrictive ventilatory defect: population variability and individual determinants. Primary Care Respiratory Journal. 2012;21(2):187-93.

43. Sperandio EF, Arantes RL, Matheus AC, Silva RP, Lauria VT, Barboza M, et al. Abstract P269: Restricted Spirometry is Associated With Cardiovascular Risk in Asymptomatic Adults Independent of the Confound Effect of the Level of Physical Activity and Fitness. Circulation. 2015;131(suppl_1):AP269-AP.
44. Tanabe N, Masuda I, Shiraishi Y, Maetani T, Hamada S, Sato A, et al. Clinical relevance of multiple confirmed preserved ratio impaired spirometry cases in adults. Respiratory Investigation. 2022;60(6):822-30.
45. Torén K, Blomberg A, Schiöler L, Malinovschi A, Backman H, Caidahl K, et al. Restrictive Spirometric Pattern and Preserved Ratio Impaired Spirometry in a Population Aged 50–64 Years. Annals of the American Thoracic Society. 2024;21(11):1524-32.
46. Tran TV, Kinney GL, Comellas A, Hoth KF, Baldomero AK, Mamary AJ, et al. Prevalence of abnormal spirometry in individuals with a smoking history and no known obstructive lung disease. Respir Med. 2023;208:107126.
47. Wan ES, Castaldi PJ, Cho MH, Hokanson JE, Regan EA, Make BJ, et al. Epidemiology, genetics, and subtyping of preserved ratio impaired spirometry (PRISm) in COPDGene. Respiratory Research. 2014;15(1):89.
48. Wan ES, Balte P, Schwartz JE, Bhatt SP, Cassano PA, Couper D, et al. Association Between Preserved Ratio Impaired Spirometry and Clinical Outcomes in US Adults. JAMA. 2021;326(22):2287-98.
49. Wang GD, A.P. M, Chen LL, Liu Q, Yan K, Weng CH, et al. Analysis of the effect of internet-of-things based portable spirometer used for pulmonary function screening in Xiamen residents. Journal of Clinical Pulmonary Medicine. 2022(2022, 27(11):):1653-8.
50. Wang C, Wang H, Cai J, Xu X. Individual and mixture analyses of the associations of phenols and phthalates with lung function among US adults. International Journal of Environmental Health Research. 2024;35(2):330-44.
51. Wijnant SRA, De Roos E, Kavousi M, Stricker BH, Terzikhan N, Lahousse L, Brusselle GG. Trajectory and mortality of preserved ratio impaired spirometry: the Rotterdam Study. European Respiratory Journal. 2020;55(1):1901217.
52. Xia J, Qiu Y, Huang L, Li W, Zou X, Wang X, et al. Prevalence, Risk Factors of Preserved Ratio Impaired Spirometry in adult in plateau: A Cross-Sectional Study. PLOS ONE. 2025;20(4):e0318546.
53. Xiao S, Ou J, Qiu W, Ye C, Li N, Chen S, et al. Risk of All-Cause Mortality in US Adults With Preserved Ratio Impaired Spirometry: An Observational Study. Int J Chron Obstruct Pulmon Dis. 2025;20:287-302.
54. Zhang Y, Peng J, Liu L, Cui H, Zang D, Wu Z, et al. Prevalence, characteristics and significant predictors for cardiovascular disease of patients with preserved ratio impaired spirometry: A 10-year prospective cohort study in China. Respiratory Medicine. 2024;222:107523.
55. Zhou M, Wang X, Yang S, Wang B, Ma J, Wang D, et al. Cross-sectional and longitudinal associations between urinary arsenic and lung function among urban Chinese adults. Science of The Total Environment. 2022;844:157028.
56. Zhou Y, Ampon MR, Abramson MJ, James AL, Maguire GP, Wood-Baker R, et al. Prevalence and characteristics of adults with preserved ratio impaired spirometry (PRISm):

Data from the BOLD Australia study. Chronic Respiratory Disease. 2025;22:14799731241312687.
57. Zieliński J, Bednarek M, Górecka D. [National Program of Early Detection and Prevention of COPD in the years 2000-2002]. Pneumonol Alergol Pol. 2005;73(2):116-21.
58. Munn Z, Moola S, Lisy K, Riitano D, Tufanaru C. Chapter 5: Systematic reviews of prevalence and incidence. In: Aromataris E, Munn Z (Editors). JBI Manual for Evidence Synthesis. JBI, 2020
59. Egger M, Smith GD, Schneider M, Minder C. Bias in meta-analysis detected by a simple, graphical test. BMJ. 1997;315(7109):629-34.
